# Single‐cell characterization of differentiation trajectories and drug resistance features in gastric cancer with peritoneal metastasis

**DOI:** 10.1002/ctm2.70054

**Published:** 2024-10-18

**Authors:** Haoxin Peng, Lei Jiang, Jiajia Yuan, Xiangrong Wu, Nan Chen, Dan Liu, Yueting Liang, Yi Xie, Keren Jia, Yanyan Li, Xujiao Feng, Jian Li, Xiaotian Zhang, Lin Shen, Yang Chen

**Affiliations:** ^1^ Department of Gastrointestinal Oncology Key Laboratory of Carcinogenesis and Translational Research (Ministry of Education, Beijing), Peking University Cancer Hospital and Institute Beijing China; ^2^ Department of Oncology Shanghai Medical College, Fudan University Shanghai China; ^3^ Department of Gastrointestinal Surgery III Key Laboratory of Carcinogenesis and Translational Research (Ministry of Education), Peking University Cancer Hospital and Institute Beijing China; ^4^ Department of Radiation Oncology Peking University Cancer Hospital and Institute Beijing China; ^5^ Department of Gastrointestinal Cancer Beijing GoBroad Hospital Beijing China

**Keywords:** drug resistance, gastric cancer peritoneal metastasis, single‐cell RNA sequencing, spatial transcriptomics

## Abstract

**Background:**

Gastric cancer patients with peritoneal metastasis (GCPM) experience a rapidly deteriorating clinical trajectory characterized by therapeutic resistance and dismal survival, particularly following the development of malignant ascites. However, the intricate dynamics within the peritoneal microenvironment (PME) during the treatment process remain largely unknown.

**Methods:**

Matched samples from primary tumours (PT), peritoneal metastases (PM), and paired pre‐treatment and post‐chemo/immunotherapy (anti‐PD‐1/PD‐L1) progression malignant ascites samples, were collected from 48 patients. These samples were subjected to single‐cell RNA sequencing (*n* = 30), multiplex immunofluorescence (*n* = 30), and spatial transcriptomics (*n* = 3). Furthermore, post hoc analyses of a phase 1 clinical trial (*n* = 20, NCT03710265) and an in‐house immunotherapy cohort (*n* = 499) were conducted to validate the findings.

**Results:**

Tracing the evolutionary trajectory of epithelial cells unveiled the terminally differentially MUC1+ cancer cells with a high epithelial‐to‐mesenchymal transition potential, and they demonstrated spatial proximity with fibroblasts and endothelial cells, correlating with poor prognosis. A significant expansion of macrophage infiltrates, which exhibited the highest proangiogenic activity, was observed in the ascites compared with PT and PM. Besides, higher C1Q+ macrophage infiltrates correlated with significantly lower GZMA+ T‐lymphocyte infiltrates in therapeutic failure cases, potentially mediated by the LGALS9‐CD45 and SPP1‐CD44 ligand–receptor interactions. In the chemoresistant group, intimate interactions between C1Q+ macrophages and fibroblasts through the complement activation pathway were found. In the group demonstrating immunoresistance, heightened TGF‐β production activity was detected in MUC1+ cancer cells, and they were skewed to interplay with C1Q+ macrophages through the GDF15‐TGF‐βR2 axis. Ultimately, post hoc analyses indicated that co‐targeting TGF‐β and PDL1 pathways may confer superior clinical benefits than sole anti‐PD‐1/PD‐L1 therapy for patients presenting with GCPM at the time of diagnosis.

**Conclusions:**

Our findings elucidated the cellular differentiation trajectories and crucial drug resistance features within PME, facilitating the exploration of effective targets for GCPM treatment.

**Highlights:**

- MUC1+ cancer cells with a high epithelial‐to‐mesenchymal transition potential and exhibiting spatial proximity to fibroblasts and endothelial cells constitute the driving force of gastric cancer peritoneal metastasis (GCPM).
- Higher C1Q+ macrophage infiltrates correlated with significantly lower GZMA+ T‐lymphocyte infiltrates within the peritoneal microenvironment in therapeutic failure cases.
- Co‐targeting TGF‐β and PDL1 pathways may confer superior clinical benefits than sole anti‐PD‐1/PD‐L1 therapy for patients presenting with GCPM at diagnosis.

## Graphical abstract

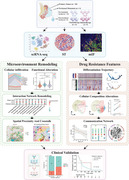

## 1 INTRODUCTION

According to the GLOBOCAN cancer statistics, gastric cancer (GC) ranked as the fifth contributor both in morbidity and mortality across cancers,^1^ with considerable disease burden in the East Asian region.^2^ Alarmingly, gastric cancer peritoneal metastasis (GCPM) affects approximately 17% of GC individuals at initial assessment.^3^ Moreover, the prognosis of GCPM patients remains dismal, with a median overall survival time of only 7 months following diagnosis, even under optimal supportive care.^4^, ^5^ Albeit the advancement in innovative therapeutic regimens like immune‐checkpoint inhibitors (ICIs) for advanced GC, the response efficacy of GCPM cases remains quite constrained.^6^, ^7^

Delving into the underlying reasons for the declining clinical course of GCPM, a limited understanding of the relevant mechanisms represents a barrier to better treatment options. Consequently, a comprehensive investigation into the onset and development of GCPM facilitates unravelling the regulating pathways and identifying novel efficacious therapeutic targets against it.

During the peritoneal metastasis from the primary tumour (PT), GC cells have to survive in the peritoneal cavity after penetrating the gastric wall and subsequently establishing tumour foci on the peritoneum.^8^ The peritoneal microenvironment (PME) is saliently intricate, comprising peritoneal exfoliated cancer cells and diverse lineages of immune cells, which experience dynamic alterations along with GCPM.^9^, ^10^ Moreover, therapeutic interventions targeting GCPM may drive the evolutionary adaptations of cells in the PME, which in turn influence the treatment efficacy.^11^, ^12^ cA few recent researches have depicted the intra‐tumoural heterogeneity and lineage variability within the peritoneal metastases.^13^ For instance, Wang et al.^14^ reported two molecular subtypes of peritoneal metastatic foci, including the mesenchymal‐ and epithelial‐like groups. The latter illustrated higher exhausted T‐lymphocyte infiltrates and higher TIM‐3 and galectin‐9 expression, suggesting potential benefits from immunotherapy. Moreover, Huang and colleagues investigated the heterogeneity of PME during GCPM and observed the cellular remodelling under therapeutic intervention. However, the sample size of ascites with available treatment information was limited (*n* = 5) and the specimens were unpaired.^13^ Consequently, the dynamic heterogeneous variations among different GCPM‐related tissues, along with the evolutionary changes of diverse cell types driven by therapies, continue to pose unresolved mysteries.

In the present study, four independent cohorts comprising matched primary tumour (PT), malignant ascites, and peritoneal metastasis (PM) samples, along with paired pre‐treatment baseline and post‐chemo/immunotherapy (anti‐PD‐1/PD‐L1) progression ascites obtained from long‐term follow‐up, were generated and underwent single‐cell RNA sequencing (scRNA‐seq) (*n* = 30), multiplex immunofluorescence (mIF) (*n* = 30), and spatial transcriptomics (ST) (*n* = 3). Moreover, post hoc analyses of phase 1 clinical trial (*n* = 20, NCT03710265) and an immunotherapy cohort (*n* = 499) within our centre were conducted to validate these findings. Overall, we aim to delineate the cellular dynamic changes among different GCPM‐related tissues and identify pivotal features that correlate with drug resistance within PME, thereby aiding in the rational design of effective targets for GCPM management in the future.

## 2 MATERIALS AND METHODS

### 2.1 Study design and specimen collection

The present study was conducted at Peking University Cancer Hospital (PKUCH), where specimens were collected from January 2021 to September 2023. To comprehensively investigate GCPM, four independent cohorts were designed: cohort 1 dynamically sampled the pre‐treatment baseline and post‐treatment progression malignant ascites after long‐term follow‐up, focusing on the therapeutic‐driven remodelling of PME; cohort 2 enrolled the paired samples from PT, ascites, and PM, simulating the GCPM process; cohort 3 and cohort 4 included PT samples from cases with or without GCPM (Figure S1).

To validate the identified drug‐resistant mechanisms revealed by scRNA‐seq, mIF, and ST, post hoc analyses on two external cohorts, including the SHR‐1701 and PKUCH Immunotherapy (PKUCHIO) cohorts, were employed. The SHR‐1701 cohort is a multicenter, first‐in‐human, and phase 1 clinical trial assessing the anti‐tumour efficacy and safety of SHR‐1701, a dual inhibitor targeting PD‐L1 and TGF‐β RII, in pre‐treated advanced solid cancers (NCT03710265).^15^ The PKUCHIO cohort comprises retrospectively collected GC cases receiving anti‐PD‐1/PD‐L1 therapy, with a median follow‐up of immune‐related progression‐free survival (irPFS) of 9.80 months (interquartile range 3.20–20.27).^16^ The therapeutic responses in both cohorts were categorized into complete response (CR), partial response (PR), stable disease (SD), and progressive disease (PD). irPFS was defined as the elapsed time from the initiation of immunotherapy to the day of disease progression, patient mortality, or the end of follow‐up.

This study was ethically approved by the PKUCH and conducted adhering to the Helsinki Declaration.^17^ Informed consent was gained from each patient.

### 2.2 Sample collection and pre‐processing for scRNA‐seq

Fresh ascites specimens were initially filtered and centrifuged at 350 g for 5 min, followed by prompt transportation on ice to the Singleron lab (Singleron Biotechnologies). Red blood cells were eliminated by incubating the samples with 2 mL GEXSCOPE red blood cell lysis buffer (Singleron) at 25°C for 10 min. Afterwards, the mixture was centrifuged at 500 × *g* for 5 min and resuspended in phosphate‐buffered saline (PBS). Trypan blue staining (Sigma) was implemented for microscopic evaluation of the sample viability.

### 2.3 scRNA‐seq construction

Single‐cell suspensions in PBS were dispensed onto a microwell chip employing the Singleron Matrix Single Cell Processing System. Barcoding Beads were extracted from the microwell chip, facilitating reverse transcription of the captured mRNA by the Barcoding Beads to generate complementary DNA (cDNA), which was subsequently amplified through Polymerase Chain Reaction. The amplified cDNA experienced fragmentation and ligation with sequencing adapters by the GEXSCOPE Single Cell RNA Library Kits (Singleron).^18^ Subsequently, individual libraries were diluted to 4 nM, pooled together, and subjected to sequencing using the Illumina Novaseq 6000 platform with 150 bp paired‐end reads.

### 2.4 Sequencing data procession and single‐cell cluster annotation

Gene expression matrices were created applying Cell Ranger software from 10× Genomics, leveraging the GRCh38 build of the human reference genome as the foundation. Analysis of the filtered gene expression matrices was performed within the Seurat package. To discern the low‐quality cells, the following quality control measures were applied: (1) cells expressing either exceeding 5000 genes or fewer than 200 genes, (2) more than 10% of unique molecular identifiers (UMI), or (3) possessing falling below 500 UMIs originating from the mitochondrial genome. Subsequent analytical procedures adhered to the standard workflow of Seurat. After normalization and automatic scaling procedures, the cellular expression matrix underwent initial summarization via principal component analysis (PCA). Subsequently, the Uniform Manifold Approximation and Projection method was employed, resulting in the formation of cell clusters exhibiting comparable features. To determine the defining characteristics of each cluster, the FindAllMarkers function was adopted to yield a definitive ranked list of marker genes based on log2 fold change in pairwise comparisons.

Cell type was initially automatically annotated employing the “singleR” R package, which facilitated impartial cell type recognition through leveraging reference transcriptomic datasets representing pure cell types.^19^ Simultaneously, canonical marker genes of each single‐cell cluster were summarized.^20^, ^21^ Eventually, cell annotation results by “singleR” algorithm underwent manual validation and correction, relying on the expression profiles of canonical marker genes and the examination of the top 30 up‐regulated genes per cluster. The purity assessment of identified cell populations was investigated via the entropy‐based ROGUE values.^22^

### 2.5 Single‐cell copy‐number variation evaluation

To recognize malignant epithelial cells (ECs), inferCNV method was exploited to compute the somatic large‐scale chromosomal copy number variation (CNV) scores of each cell in the EC cluster, along with the T‐lymphocyte cluster using the reference cells.^23^ The inferCNV algorithm utilizes a Hidden Markov Model to calculate CNV values as well as the Bayesian latent mixture model to evaluate the posterior probabilities of the alteration status for reducing the false‐positive CNV calls. Eventually, a hierarchically clustered heatmap was yielded based on CNV level per cell.

### 2.6 Trajectory inference analysis

To map the conversion of distinct cell subtypes during differentiation, pseudotime trajectory analysis was performed using Monocle2.^24^ Dimensionality reduction was conducted by the DDRTree method based on markedly differentially expressed genes (DEGs). Then the pseudotime‐based cell lineage trajectories were inferred using default parameters. Simultaneously, the BEAM test within Monocle 2 was also executed to identify the dynamic alterations of genes along each branch.

### 2.7 Cellular interaction networks interrogation

The CellChat algorithm was exploited to deduce, analyze, and depict intercellular communications among distinct cell populations.^25^ The compilation of known ligand–receptor pairs was sourced from CellChatDB, a literature‐curated repository of ligand and receptor interactions. Initially, overexpressed ligands or receptors across different cell types were identified. Subsequently, the estimation of intercellular communication probabilities was determined through the computation of all ligand–receptor interactions pertaining to individual signalling pathways. The iTALK algorithm was also employed to explore the intercellular communication networks, encompassing four distinct ligand–receptor categories: cytokines, immune checkpoints, growth factors, and others.^26^ The NicheNet method, capable of elucidating the intracellular gene regulatory effects and signal transductions, was employed to dissect which ligand–receptor crosstalk potentially influences the transcriptomic alterations in the signal receiver cell types.^27^

### 2.8 Evaluation of the functional signatures and cell signatures

The well‐known functional signatures, including angiogenesis, cytotoxic, exhausted, epithelial–to–mesenchymal transition (EMT), HLA (antigen presentation [AP]), inflammation‐promoting, proliferative, transforming growth factor‐β (TGF‐β) response (TBRS), M1_signature, and M2_signature, were employed to evaluate the functional scores of single‐cell clusters using the AddModuleScore function in Seurat (Table S1).^28^, ^29^, ^30^, ^31^ The top DEGs within various cell subclusters served as corresponding cell signatures, representing specific cell types (Table S2). The cell signatures were calculated by the single‐sample gene set enrichment analysis approach, which transforms particular gene expression profiles into the abundance of cell clusters in a single sample at the bulk level.^32^

### 2.9 Identification of DEGs and gene set variation analysis

DEGs were identified by the limma package according to the statistical threshold of log_2_(fold change) >1 and Bonferroni‐corrected *p*_adj _< .01.^33^ Pathway activity of distinct cell populations was assigned via gene set variation analysis based on the MSigDB databases, which included the biological process, cellular component, and molecular function categories as reference.^34^

### 2.10 Histopathological assessment and ST sequencing

The GC tissues were embedded in paraffin prior to RNA extraction. Each tumour section was sliced to a thickness of 10 µm and affixed onto ST microarrays for subsequent analysis. Afterwards, the tissue underwent dehydration in isopropanol for 1 min, followed by staining with hematoxylin–eosin (H&E).

Bright‐field photos were captured utilizing a whole slide scanner at a magnification of 20× resolution. H&E‐stained slides were then thoroughly examined by two experienced pathologists (Dr. Yajie Hu and Dr. Yu Sun) for pathology confirmation and manual annotation of distinct tissue regions. Subsequently, tissue sections were processed with Visium Spatial Gene Expression Reagent Kits (10× Genomics) for library construction and sequencing, adhering to the standard protocols.^35^

### 2.11 ST data processing

The raw sequencing data of ST underwent quality assessment and alignment using Spaceranger (version 1.1.0.). The ST data underwent qualitative evaluation utilizing parameters encompassing total spots and mean UMIs/genes/mitochondrial genes per spot. Genes with a measured count below 200/spot or expressed in fewer than three spots were excluded. The LogVMR function was implemented to standardize the data across spots. Subsequently, dimensionality reduction and clustering were conducted using PCA. The SCTransform function mapped distinct cell types to the ST sections based on scRNA‐seq data. Moreover, the cell signature scores within each spot were quantified by the AddModuleScore function.

### 2.12 Multiplex immunofluorescence detection

To validate the findings from scRNA‐seq and ST, the mIF approach was conducted to detect the expression levels and spatial distribution of panCK, CD3, CD68, CD31, FAP, and MUC1 in situ. Information on antibodies used to detect each molecule is listed in Table S3.

The tumour tissues were promptly fixed in formalin for 24 to 48 h after resection, followed by dehydration and embedding in paraffin. Subsequently, each paraffin block tissue was cut to a 4 µm thickness, which was melted and dehydrated at 60°C for 12 h. Subsequently, the samples underwent deparaffinization and rehydration processes. Paraffin slides were then immersed in EDTA (pH 9.0) or citrate buffer (pH 6.0), and the entire system was subjected to heat treatment for antigen retrieval. Then the slides were sequentially incubated with primary and secondary antibodies conjugated with horseradish peroxidase. Tyramine signal amplification was carried out, succeeded by antibody removal and antigen retrieval through slide heating. Cell nuclei were stained with 4′, 6‐diamidino‐2‐phenylindole.

Whole slide bright‐field and fluorescence images were captured through the Mantra quantitative pathology imaging system (PerkinElmer). The tumour core (TC) was delineated by two pathologists via the Phenochart software. Then the inForm software (PerkinElmer) was utilized to segment and annotate cells in the multispectral images, resulting in information on cellular abundance and distribution (Figure S2A), as we previously reported.^36^, ^37^

Cellular number, density, and ratio were used to evaluate the cellular abundance, while effective score (ES) and effective percentage (EP) were employed to estimate the spatial relationships among different cell types. Previous studies indicated that a distance of 20 µm is an appropriate radius for performing spatial computations of cellular components.^38^, ^39^ Consequently, the formulae for calculating ES and EP are as follows (Figure 2D):

$$\mathrm{ES}\;=\frac{\mathrm{Number}\;\mathrm{of}\;\mathrm{surrounding}\;\mathrm{cells}}{\mathrm{Number}\;\mathrm{of}\;\mathrm{central}\;\mathrm{cells}};$$

$$\mathrm{EP}\;=\frac{\;\mathrm{Number}\;\mathrm{of}\;\mathrm{center}\;\mathrm{cells}\;\mathrm{surrounded}\;\mathrm{by}\;\mathrm{surrounding}\;\mathrm{cells}}{\mathrm{Number}\;\mathrm{of}\;\mathrm{center}\;\mathrm{cells}}.$$

Generally, the higher ES or EP is, the closer the spatial distribution between two cell types is.

### 2.13 Survival analysis based on bulk RNA‐seq data

The bulk RNA‐seq data as well as matched survival information of GC patients in the TCGA‐STAD cohort were retrieved from the UCSC Xena database^40^ (acquisition date, 29 March 2023).

### 2.14 Prediction of drugs targeting major cell types implicated in treatment resistance

The DREIMT algorithm, which is enabled by a curated database integrating up to 4960 drug profiles with approximately 2600 immune gene expression signatures, was employed to predict drugs that target major cell populations involved in treatment resistance.^41^

### 2.15 Statistical analysis

For continuous variables, the Mann–Whitney *U* test and Wilcoxon *t*‐test were employed to assess differences in means between the two groups. Categorical variables were compared using either the rank sum test or the chi‐square test. Distinctions of Kaplan–Meier survival curves were evaluated by the log‐rank test. The prognostic effects of cell signatures were further investigated by the univariate Cox regression analyses. To interrogate the most appropriate cutoff point, the outcome‐driven X‐tile software was used.^42^ Graphical representations and statistical evaluations were conducted by R (version 4.0.4), SPSS (version 25.0), and X‐tile (version 3.6.1).

## 3 RESULTS

### 3.1 Clinicopathologic features of the included patients

**FIGURE 1:**
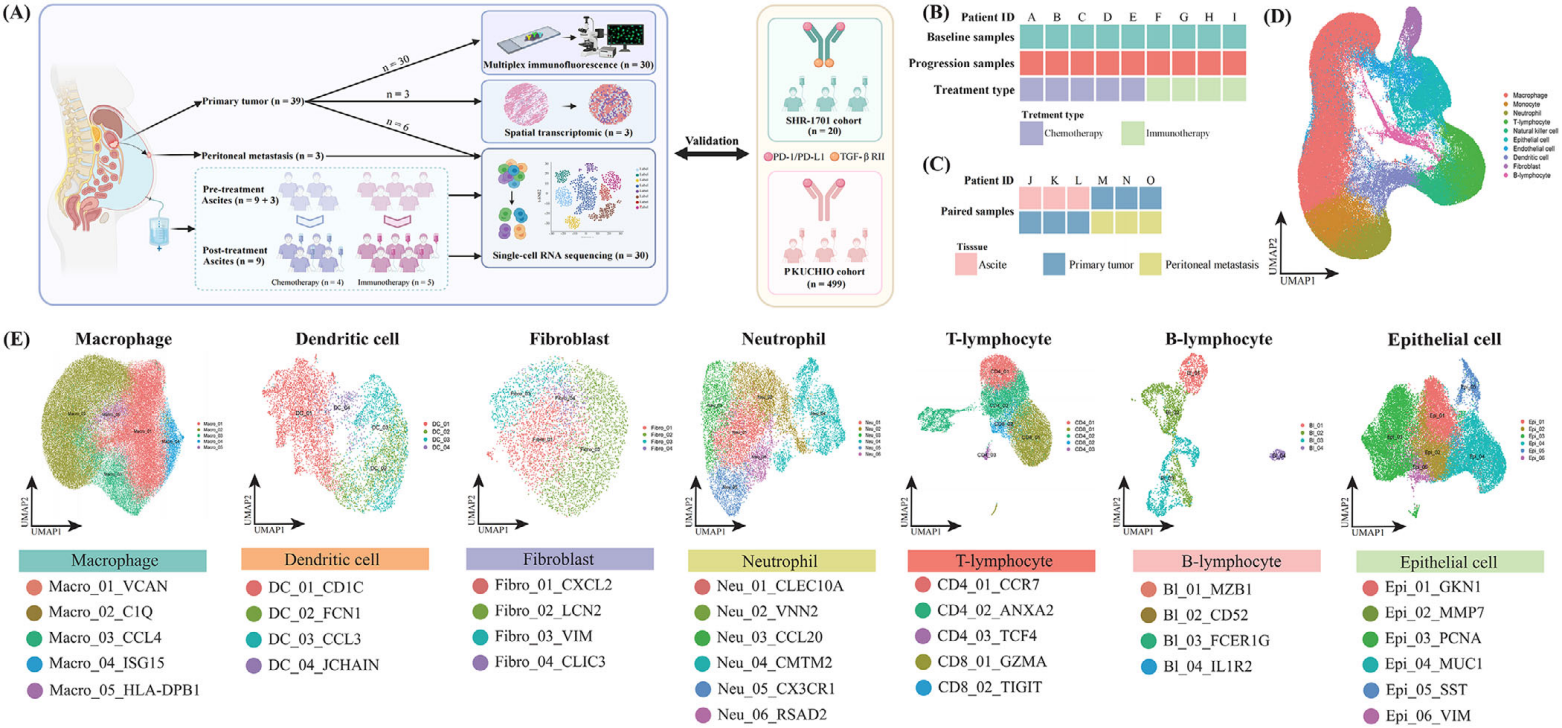
The single‐cell atlas of patients with gastric cancer peritoneal metastasis. The experimental workflow with the denoted information of numbers of samples (A), as well as treatment regimens (B) and tissue source sites (C) of samples detected in the single‐cell RNA‐sequencing approach. UMAP plots presenting the major cell populations (D). Dots indicate individual cells and different colours correspond to different cell types (E).

A total of 63 samples were collected from 48 patients, comprising thirty‐nine from PT, twenty‐one from ascites, and three from PM (Figure 1A, Figure S1). Cohort 1 encompassed 9 pairs of pre‐treatment baseline and post‐treatment progression samples of ascites with a follow‐up period ranging from 11 to 181 days, in which four patients received chemotherapy and five underwent immunotherapy (anti‐PD‐1/PD‐L1) (Figure 1B, Table S4). Cohort 2 included matched samples from PT (*n* = 6), ascites (*n* = 3), and PM (*n* = 3), simulating the process of tumour cells detaching from the PT site, disseminating into the abdominal cavity, and colonizing on the peritoneum (Figure 1C, Table S5). Cohort 3 enrolled three PT samples for the validation in ST sequencing. Cohort 4 recruited thirty PT samples, including fifteen with GCPM and fifteen without GCPM (Table S6). In aggregate, 30 samples were detected by scRNA‐seq, 3 samples were subjected to ST, and 30 samples were examined by mIF. The majority of the enrolled cases presented with diffuse‐type GC (21/48, 44%) and were male (35/48, 73%). Additionally, twenty GC cases from the SHR‐1701 cohort (Table 7) and 499 cases from the PKUCHIO cohort (Table S8) were enrolled. Moreover, 9 (9/20, 45%) and 155 patients (155/499, 31%) presented with GCPM at the time of diagnosis were identified in the SHR‐1701 and PKUCHIO cohort, respectively.

### 3.2 Cellular dynamic alterations among PT, ascites, and PM

A total of 248,046 high‐quality cells, including 150 156 cells in cohort 1 (Figure 3A–C) and 97 890 cells in cohort 2 (Figure S3D–F), were identified ultimately (Figure 1D). Cells could be grouped into ten major lineages upon automatic annotation and manual correction, including B‐lymphocytes, dendritic cells (DC), endothelial cells, ECs, fibroblasts, macrophages, monocytes, natural killer (NK) cells, neutrophils, and T‐lymphocytes (Figure 1E, Figures S4A–S and S5). The ROGUE levels of discerned single‐cell clusters within cohort 1 and cohort 2 mostly surpassed 0.7, indicating high purity (Figure S4T–U).

**FIGURE 2:**
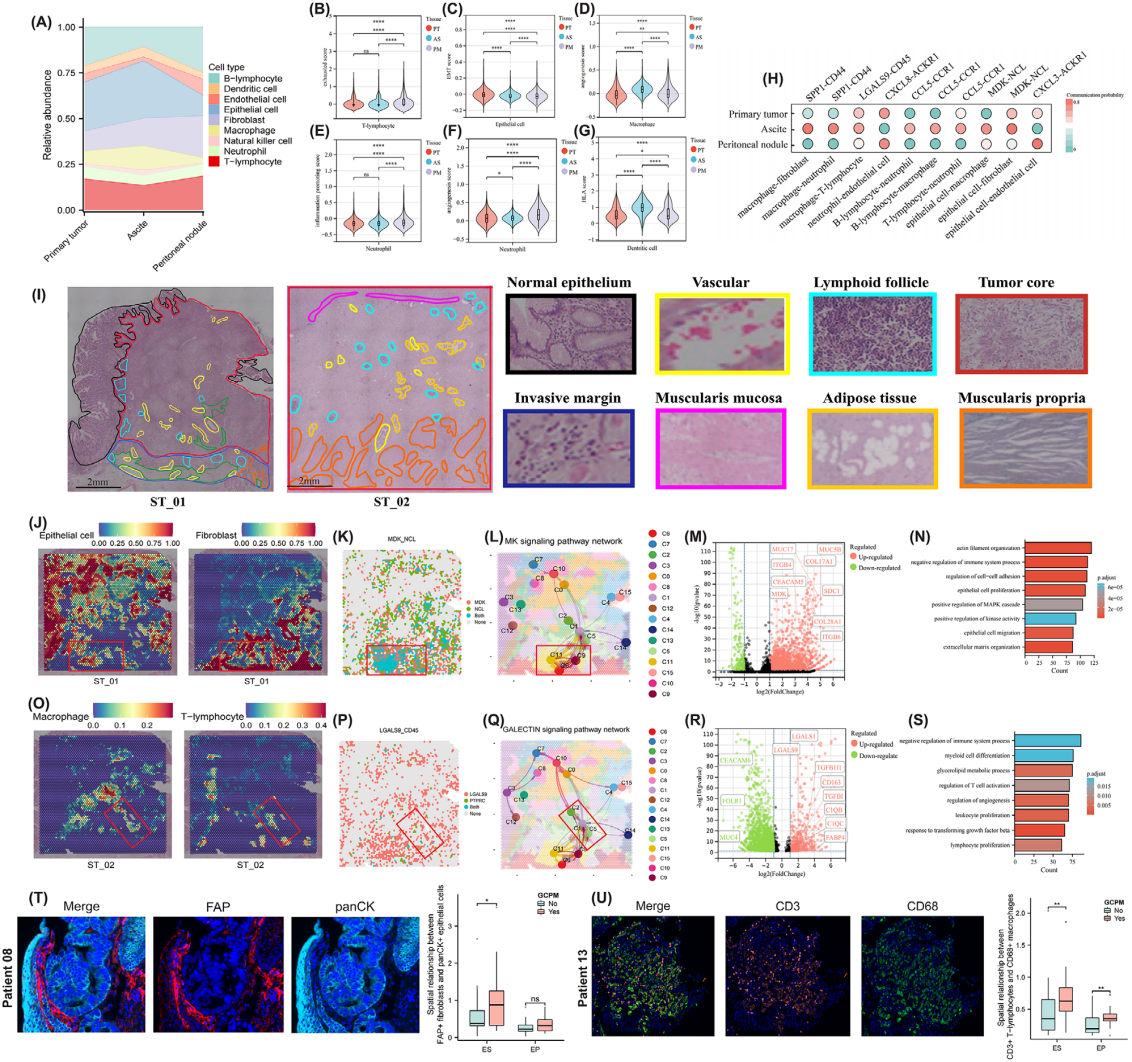
Microenvironment dynamic changes during gastric cancer peritoneal metastasis (GCPM). Cellular composition (A), functional scores (including the exhausted scores of T‐lymphocytes (B), epithelial to mesenchymal transition scores of epithelial cells (C), pro‐angiogenesis scores of macrophages (D), pro‐inflammation (E) and pro‐angiogenesis (F) scores of neutrophils, and HLA scores of dendritic cells (G), and communication network alterations in the process of GCPM (H). Different tissue regions are delineated by different colours on the H&E‐stained pathological image of GC from two cases undergoing spatial transcriptomic sequencing (I). Scale bar = 2 mm and 100 µm for the whole tissue and magnified images, respectively. Epithelial cells (ECs) and fibroblasts colocalize in the area of invasive margin (IM) (J). The potential ligand–receptor pair (K) and corresponding signalling pathway (L) mediate their crosstalk. Differentially expressed genes (DEGs) (M) and corresponding enriched pathways (N) in the IM than tumour core (TC) regions. Macrophages and T‐lymphocytes tend to colocalize in TC (O). The potential ligand–receptor pair (P) and corresponding signalling pathway (W) mediate their communication. DEGs (R) and relevant enriched biological pathways (S) in the TC than the lymphoid follicle regions. The multiplex immunofluorescence analyses indicated the spatial proximity between panCK+ ECs and FAP+ fibroblasts (T) and CD3+ T‐lymphocytes and CD68+ macrophages (U). *p < .05; ****p < .0001; ES, effective score; EP, effective percentage; ns, non‐significant.

Cell clusters displayed distinct preferences for specific tissues, with endothelial cells and fibroblasts showing increasing infiltrates, while T‐lymphocytes displayed opposing trends from PT to ascites and PM (Figure 2A). Notably, B‐lymphocyte infiltrates were significantly higher in the PT compared with ascites and PM (Figure 6A–B). Functional signatures were further employed to delineate the functional alterations of cell types. The exhausted scores of T‐lymphocytes gradually augmented from PT to PM, indicating a diminishing anti‐tumour immunity (Figure 2B). The EMT scores of ECs, representing the migratory and invasive potential, remained highest in the PT while lowest in the PM (Figure 2C). As the dominant cell types in ascites, macrophages demonstrated the highest pro‐angiogenic capacity compared with PT and PM, exhibiting a propensity to be pro‐tumour phenotype (Figure 2D). Likewise, neutrophils showed high pro‐inflammation (Figure 2E) and pro‐angiogenic (Figure 2F) activity in the ascites. Interestingly, DCs manifested higher AP scores in the ascites (Figure 2G), indicating active antigen‐specific immunity. The mIF method indicated consistent findings that significantly higher infiltrating levels of CD31+ endothelial cells and FAP+ fibroblasts existed in cases with GCPM than without (Figure S2B). Moreover, CD31+ endothelial cell infiltrates were detected to be higher in the tumour core (TC) than in the invasive margin (IM) (Figure S2C). Furthermore, higher ES between tumour cells and CD31+ endothelial cells and FAP+ fibroblasts were discerned in cases with GCPM than without, indicating closer spatial location among them (Figure S2E).

We then interrogated the discrepancies of co‐infiltrated patterns among different tissue types. We discovered that infiltrates of macrophages positively correlated with neutrophils in both PT, ascites, and PM, while a co‐infiltrated pattern between fibroblasts and endothelial cells was only discerned in the PT (Figure 6C–E). To move forward, we delved into the remodelling of cellular interaction networks via the CellChat algorithm (Figure 2H). Consistent with the co‐infiltration findings, enhanced communication probability was observed from macrophages to fibroblasts and neutrophils through SPP1‐CD44 interaction in ascites. In contrast, closer interactions between ECs and endothelial cells were discerned in PT and PM. Intriguingly, crosstalk between macrophages and T‐lymphocytes through the galectin‐9 (LGALS9)‐CD45 axis remained intimate irrespective of tissue types. Moreover, interactions between ECs and fibroblasts via midkine (MDK/MK)‐NCL ligand–receptor pair augmented in the PT and ascites compared with the PM.

### 3.3 Integrated scRNA‐seq and ST analysis revealed cellular interaction networks in the PT

To further validate the cellular interaction networks revealed by the scRNA‐seq at a spatial level, ST analysis of three PT cases from patients with GCPM at the time of diagnosis was adopted. Following the application of H&E staining and bright‐field microscopy, the slide was delineated into distinct regions, containing the normal epithelium, vascular, lymphoid follicle, TC, IM, muscularis mucosa, adipose tissue, and muscularis propria (Figure 2I). Transcriptomes from 14 804 spots in three sections were gained at a median depth of 3347 UMIs and 2094 genes per spot. To profile the spatial characteristics of major cell populations in the PT, scRNA data of an additional four PT samples were mapped to the ST slides via the SCTransform approach (Table S9, Figure S7). As expected, the spatial features closely recapitulated the histological architecture of the tissue. For instance, the TC region was enriched with ECs, while T‐lymphocytes and B lymphocytes were abundant in the lymphoid follicle. Additionally, a total of 16, 15, and 15 clusters were grouped by the UMAP analysis in the ST_01 (Figure S8A), ST_02 (Figure S8B), and ST_03 (Figure S8C) samples, respectively.

ECs and fibroblasts tended to co‐localize in the IM on the ST slides, with significant co‐expression of MDK‐NCL ligand–receptor pair observed (Figure 2J). Moreover, the MDK‐mediated pathway was also abundant in this region, corroborating the findings by the CellChat algorithm (Figure 2K,L). We further explored the biological differences between the ECs‐dominant regions with (C6) and without (C2) the co‐infiltration of fibroblasts. Notable upregulation of ECs‐related (e.g., MUC17) and fibroblast‐related (e.g., COL17A1) genes, as well as MDK, were found in C6 (Figure 2M) than C2. The DEGs between C6 and C2 were enriched into EC proliferation and migration pathways, indicating the malignant invasive potential of ECs in the IM area (Figure 2N). In brief, intimate correlations between ECs and fibroblasts in the IM of PT may contribute to GCPM.

We additionally validated the crosstalk between macrophages and T‐lymphocytes suggested by the CellChat algorithm on ST slides in the same manner. We found that they tend to co‐localize in the TC region (Figure 2O), wherein the galectin pathway was enriched (Figure 2P∼2Q). Moreover, significantly higher expression levels of galectin and TGF‐β were discerned in the co‐infiltrated region (Figure 2R∼2S). Moreover, negative regulation of anti‐tumour immunity‐related pathways was also enriched, suggesting an immunosuppressive niche. Similar analyses on the ST_02 (Figure S8D–H) and ST_03 (Figure S8I–M) cases validated these findings. The mIF approach demonstrated consistent results with ST and scRNA‐seq analyses that spatial proximity existed between panCK+ ECs and FAP+ fibroblasts (Figure 2T) and between CD3+ T‐lymphocytes and CD68+ macrophages (Figure 2U) in cases with GCPM than without.

### 3.4 Identification and tracing of the differentiation trajectory of ECs

**FIGURE 3:**
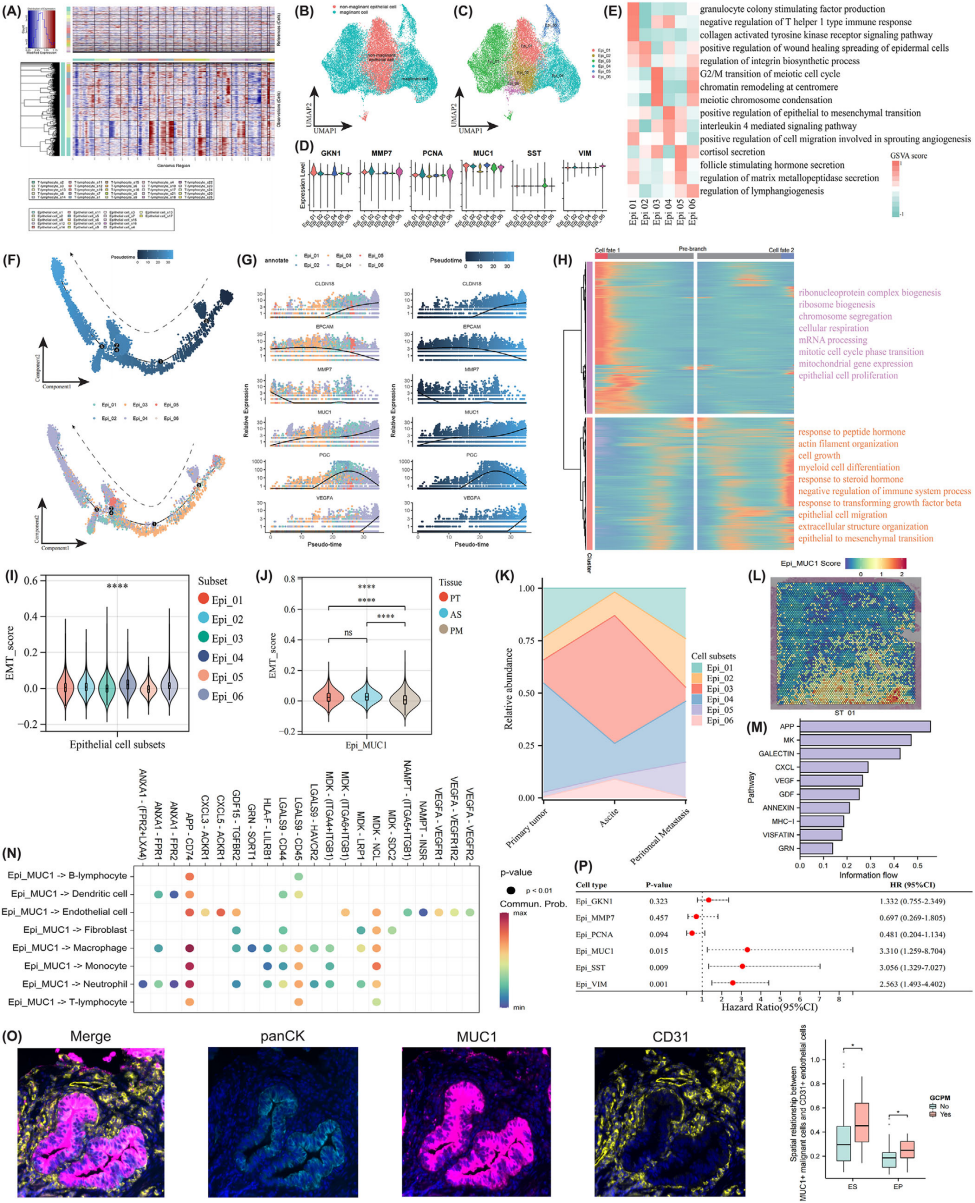
Identification of malignant epithelial cells (EC) and their differentiation trajectory. The hierarchical heatmap demonstrates the large‐scale copy number variation (CNV) profile of each EC cluster within ascites, with red and blue colours signifying high and low CNV levels, respectively. UMAP plots illustrate malignant/non‐malignant ECs (B), along with their subclusters (C). Violin plots exhibit the expression patterns of marker genes specific to each EC subtype (D). Heatmap visualizes the pathway activities of each EC subtype, as quantified using the gene set variation analysis method (E). The differentiation trajectory of ECs along pseudotime is traced, with each dot corresponding to an individual cell (F). Dynamic alterations of function‐related genes during the transitional trajectory, including CLDN18, EPCAM, MMP7, MUC1, PGC, and VEGFA (G), among which these genes could be hierarchically clustered into two groups with distinct enriched pathways (H). The epithelial‐to‐mesenchymal transition (EMT) activity among different EC subtypes (I). Discrepancies of EMT activity of MUC1+ ECs among primary tumour, ascites, and peritoneal metastasis (J). Dynamic changes in the composition of distinct EC subtypes during gastric cancer peritoneal metastasis (K). The spatial distribution and signature score of MUC1+ malignant ECs within tissue sections (L). The information flow (M) and interaction probabilities between MUC1+ malignant ECs and other cell types are mediated by ligand–receptor pairs (N). The multiplex immunofluorescence detection indicated the spatial proximity between MUC1+ ECs and CD31+ endothelial cells (O). Forest plot showing the prognostic values of each EC subcluster in the TCGA‐STAD cohort, as evaluated by the univariate Cox regression analysis (*p*). ****p < .0001.

Significantly higher CNV scores on chromosomes 7, 8, 9, and 10 on EC subgroups S1 and S2 were found, thereby assigning them malignant ECs (Figure 3A). All ECs could be grouped into six clusters, with GKN1 (Epi_01), MMP7 (Epi_02), PCNA (Epi_03), MUC1 (Epi_04), SST (Epi_05), and VIM (Epi_06) serving as the cluster‐specific genes, respectively (Figure 3B–D). Accordingly, GKN1+ ECs, MUC1+ ECs, and VIM+ ECs were regarded as cancer cells. GSVA analysis was adopted to assign pathway activity among these six EC clusters, underscoring the PCNA+ ECs with high proliferation ability, MUC1+ ECs with high EMT potential, and VIM+ ECs with enhanced lymphangiogenesis capacity (Figure 3E).

To comprehend the dynamic transitional mechanisms of ECs among different tissues, pseudotime trajectory analysis was further conducted. The trajectory was delineated to originate from Epi_03, traverse through the intermediate states of Epi_01 and Epi_05, and culminate in a terminal state of Epi_04 (Figure 3F). The expression levels of MUC1 and VEGFA gradually augmented, whereas CLDN18 and MMP7 progressively diminished along the trajectory (Figure 3G). Moreover, a total of 6856 genes showing varying expression spectrums were recognized along the ECs transition, which could be grouped into two patterns. Genes in cluster 1 and cluster 2 both highly expressed in the late‐stage while correlated with distinct pathway activities, including the EC proliferation and EMT processes, respectively (Figure 3H). We further assessed the EMT activity disparities among different EC subclusters and found that MUC1+ ECs displayed the highest values (Figure 3I). The EMT function of MUC1+ ECs remained elevated in PT and ascites (Figure 3J), whereas it attenuated in PM, wherein the ECs may experience mesenchymal‐to‐epithelial transition instead. The cellular composition of distinct EC subtypes also showed dynamic alterations (Figure 3K). Additionally, ST analysis revealed that the MUC1+ ECs tended to distribute in the IM region of PT (Figure 3L).

The communication networks from MUC1+ ECs to other cell types were examined, revealing that MK, galectin, CXCL, and VEGF pathways were the predominant ones (Figure 3M). Ligand–receptor analyses further showed that MUC1+ ECs may inhibit the activation of T‐lymphocytes via LGALS9‐CD45 axis^43^ while promoting angiogenesis through interaction with endothelial cells via CXCL3/CXCL5‐ACKR1 axes^44^ (Figure 3N). The mIF detection also indicated the spatial proximity between MUC1+ ECs and CD31+ endothelial cells (Figure 3O). Eventually, we investigated the prognostic implications of EC subclusters represented by specific gene signatures, demonstrating that higher MUC1+, SST+, and VIM+ EC infiltrates portended a dismal prognosis (Figure 3P). Taken together, ECs underwent phenotypically and functionally reconstructing, and MUC1+ cancer cells with high EMT activity played a key role in driving GCPM.

### 3.5 PME characteristics of treatment resistance cases

**FIGURE 4:**
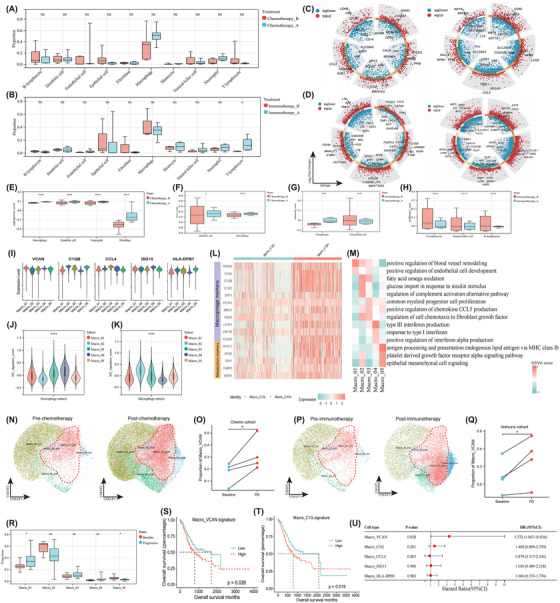
Treatment‐induced malignant ascites remodelling and macrophage subsets profiling. Cellular composition changes induced by chemotherapy (A) and immunotherapy (B). Differentially expressed genes and functional alterations, including pro‐angiogenesis, antigen presentation, exhausted and proliferation scores of different cell types in post‐treatment progression versus pre‐treatment baseline cases receiving chemotherapy (C, E, F) and immunotherapy (D, G, H). Violin plots showing the marker genes of each macrophage subtype (I). M1 (J) and M2 (K) signature scores of various macrophage subsets. Heatmap displaying the differentially expressed genes, including macrophage and metabolism‐related markers, between C1Q+ and C1Q‐ macrophages (L). Heatmap displaying the pathway activities of individual macrophage subtypes, quantified using the gene set variation analysis method (M). UMAP plots reveal the distinct cellular composition of macrophage subtypes in post‐ versus pre‐chemotherapy (N)/immunotherapy (P) samples. A significant increase in the proportion of VCAN+ macrophages in cases of resistance to chemotherapy (O) and immunotherapy (Q) was observed. Cellular composition changes of different macrophage subtypes in post‐ than pre‐treatment cases (R). Kaplan–Meier curves indicate the prognostic effects of VCAN+ macrophage (S) and C1Q+ macrophage (T) signatures. The forest plot illustrates the prognostic values of each macrophage subcluster in the TCGA‐STAD cohort, assessed via the univariate Cox regression analysis (U). *p < .05; ****p < .0001; ns, non‐significant.

We then focused on the PME remodelling based on nine pairs of pre‐treatment baseline and post‐treatment progression samples. Significant expansion of neutrophils was observed in treatment progression than baseline ascites samples (Figure S9A). In the chemotherapy cohort, a decreasing trend of EC infiltrates was observed, aligning with the therapy‐driven apoptosis and necrosis of malignant cells, whereas macrophage showed uptrend infiltration (Figure 4A). T‐lymphocyte infiltrates significantly expanded after immunotherapy (Figure 4B), indicating the reinvigoration of antitumour immunity.

Next, we parsed the DEGs and related pathway activity changes after chemo/immunotherapy. Notable up‐regulation of CXCL8 and IL1B in macrophages and NFKBIZ in neutrophils in the chemoresistant group was observed (Figure 4C), indicating the augmented proinflammation function of myeloid‐derived suppressor cells (MDSC) (Figure S9B–D). In the immunotherapy group, upregulation of response to interferon‐gamma (IFN‐γ) of T‐lymphocytes was observed (Figure 4D, Figure S9E–G). The functional signatures of distinct cell types were also evaluated, and MDSCs from progression samples exhibited higher pro‐angiogenic activities following chemotherapy (Figure 4E). Simultaneously, the AP function of DCs and macrophages also enhanced (Figure 4F), possibly attributed to the chemotherapy‐induced immunogenic cell death.^45^ In cases resistant to immunotherapy, higher exhausted scores (Figure 4G) and lower proliferative scores (Figure 4H) of T‐lymphocytes were noted. Similar phenotypes were also discerned in other immune effector cells like NK cells and B‐lymphocytes, suggesting a state of immunodepletion. The intercellular communication networks were also distinct between progression and baseline samples, with markedly strengthened interactions among macrophages, fibroblasts, and neutrophils via CCL pathways in treatment‐failure individuals (Figure S10). Briefly, enhanced pro‐inflammation/angiogenesis activities of MDSCs and T‐lymphocyte exhaustion were the driving forces of therapeutic failure.

### 3.6 Tracing the evolution of macrophages during therapeutic interventions

Subsequently, we procured data from macrophages for in‐depth sub‐clustering, generating five distinct subgroups that we designated Macro_01 to Macro_05, with VCAN, C1QB, CCL4, ISG15, and HLA‐DPB1 as respective marker gene (Figure 4I). Then we assessed the pro‐inflammatory M1 (Figure 4J) and anti‐inflammatory M2 (Figure 4K) signature scores across these subpopulations, which is a well‐known binary classification of macrophage.^46^ Intriguingly, the C1QB+ macrophages exhibited lowest M1_score and highest M2_score, thereby being deemed as tumour‐associated macrophages (TAMs). The expression profiles were also distinct between C1Q+ and C1Q− macrophages, with C1Q+ TAMs showing elevated expression levels of pro‐tumour molecules like TREM2 and VSIG4, and metabolism‐related markers like APOE and FABP5 (Figure 4L).^47^ GSVA analysis was further implemented to validate these findings, confirming that pro‐angiogenesis signalling was the hallmark of Macro_01; lipid metabolism and complement activation characterized Macro_02, pro‐inflammation activity was typically seen in Macro_03, Macro_04 was IFN‐primed, and Macro_05 exhibited high AP capacity (Figure 4M).

The ontology of macrophage differentiation was explored. Macro_05 was termed as the onset and it transited into Macro_01/ Macro_02 or Macro_04 ultimately (Figure S11A). Expression levels of metabolism‐related genes, such as APOE, FABP5, and IDH1, as well as TAM‐specific genes like SPP1, TREM2, and VCAN, gradually increased along the pseudotime transition (Figure S11B). Dynamic transcriptomic patterns could be roughly grouped into three clusters: cluster 1 contained genes highly activated in the late stage, which correlated with angiogenesis and glycerolipid metabolism; genes in clusters 2 and 3 progressively diminished along the trajectory, being enriched into innate immune response (Figure S11C).

The cellular composition of various macrophage subsets also dynamically changed. We found that VCAN+ macrophages significantly amplified in both chemo‐ (Figure 4N,O) and immunoresistant (Figure 4P,Q) cases. In contrast, HLA‐DPB1+ macrophages markedly decreased in the progression samples (Figure 4R). Additionally, higher VCAN+ (Figure 4S) and C1Q+ macrophage (Figure 4T) infiltrates predicted worse prognoses (Figure 4U). Taken together, VCAN+ macrophages with elevated pro‐angiogenic function and C1Q+ macrophages with high lipid‐metabolism activity were the key drivers contributing to therapeutic failure.

### 3.7 Characterization of T‐lymphocytes and their interactions with macrophages

**FIGURE 5:**
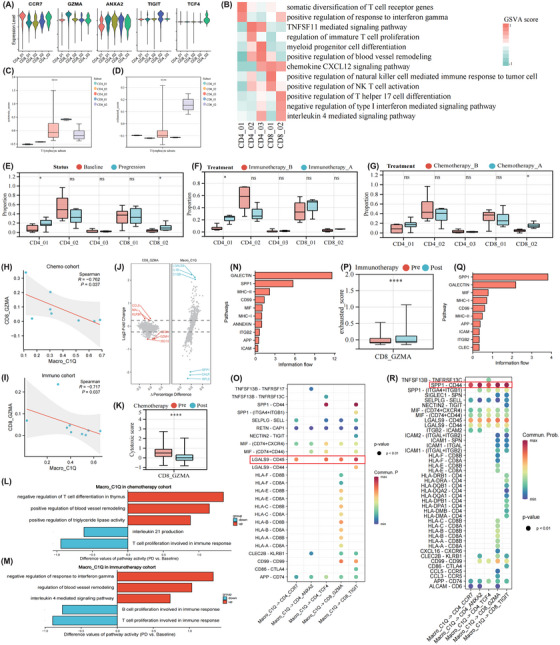
Dissecting the T‐lymphocyte in ascites microenvironment at single‐cell level. Expression profiles of canonical marker genes (A) and functional disparities (B) between different T‐lymphocyte subsets. Cytotoxic (C) and exhausted (D) scores differences between different subpopulations of T‐lymphocyte as evaluated by the single‐sample Gene Set Enrichment Analysis approach. Histogram depicting changes in cellular proportions of distinct T‐lymphocyte subsets in progression samples compared with baseline (E). Alterations in cellular proportions of various T‐lymphocyte subtypes between pre‐ and post‐immunotherapy (F)/chemotherapy (G) cases. Scatterplots demonstrating the numerical associations between C1Q+ macrophages and GZMA+ cytotoxic T‐lymphocytes (CTL) in chemotherapy (H) and immunotherapy (I) cohorts. Volcano plots highlight differentially expressed genes in C1Q+ macrophages and GZMA+ CTLs in progression compared with baseline samples receiving chemotherapy (J). The cytotoxic effect changes of GZMA+ CTLs in post‐ than pre‐chemotherapy samples (K). Gene set variation analyses underscore the function activity alterations of C1Q+ macrophages in chemo (L)/immunotherapy (M) cohorts. Differences in the overall information flow of each signal pathway with C1Q+ macrophages serving as signal transmitters and their interaction probabilities with T‐lymphocytes mediated by ligand–receptor pairs between chemotherapy (N, O) and immunotherapy cohorts (Q, R). The exhausted score changes of GZMA+ CTLs in post‐ than pre‐immunotherapy samples (*p*). *p < .05; ****p < .0001; ns, non‐significant.

To decipher the transcriptional features of T‐lymphocytes, they were re‐clustering into five diverse subsets, including CCR7+ (CD4_01), ANXA2+ (CD4_02), TCF4+ (CD4_03), GZMA+ (CD8_01), and TIGIT+ (CD8_02) T‐lymphocytes (Figure 5A). GSVA and functional signature analyses pinpointed that CCR7+, GZMA+, and TIGIT+ T‐lymphocytes were skewed to be naïve, effective memory, and exhausted states, respectively (Figure 5B–D, Figure S7A–F).^20^, ^48^ Pseudotime analysis further demonstrated that the CCR7+ subset served as the initiation of differentiation, with the GZMA+ subset as the intermediate status and eventually reaching the state represented by ANXA2+ or TIGIT+ subtypes (Figure S11D). Interestingly, the expression levels of GZMA displayed an initial elevation followed by a subsequent decline, while ZFP36L1 expression, which modulates the fate of CD8+ T‐lymphocytes, gradually enhanced (Figure S11E).^49^ Consequently, the developmental trajectory of T‐lymphocytes experienced initial activation and subsequent exhaustion (Figure S11F).

We also identified therapy‐induced cellular composition alterations of T‐lymphocytes (Figure 5E), showing the expansion of CCR7+ and TIGIT+ subtypes in the immuno‐ and (Figure 5F) chemoresistant (Figure 5G) groups, respectively. Intriguingly, significant negative associations between C1Q+ TAMs and GZMA+ cytotoxic T‐lymphocytes (CTLs) infiltration in ascites were found in both the chemo‐ (Figure 5H) and immunoresistant (Figure 5I) cohorts. Moreover, markedly down‐regulated expression of cytotoxicity‐related genes like GZMH and CD8A of GZMA+ CTLs was discerned from the chemotherapy cohort (Figure 5J). Accordingly, their cytotoxic scores (Figure 5K) decreased, while their exhaustion scores (Figure 5P) increased. Inversely, notable up‐regulation of LGALS9, the ligand of TIM‐3,^50^ and C1QB in C1Q+ TAMs was highlighted. GSVA implied the negative regulation of response to IFN‐γ and enhanced IL4‐mediated signalling activities of C1Q+ TAMs in treatment progress cases (Figure 5L,M).

To elucidate the underlying mechanisms of macrophage‐T‐lymphocyte crosstalk that contribute to therapeutic inefficacy, intercellular and intracellular interaction networks between them were profiled. CellChat algorithm speculated that the galectin, SPP1, and MHC‐II were the leading signalling pathways mediating their interplay in the chemoresistant cohort (Figure 5N), wherein LGALS9‐CD45 axis maintaining strengthened across different T‐lymphocyte subsets (Figure 5O). In the immunoresistant cohort, their crosstalk through the SPP1‐CD44 ligand–receptor pair notably intensified (Figure 5Q,R). The C1Q+ TAMs were further postulated to possess a strong regulatory capacity of GZMA+ CTLs through targeting their activation‐dependent gene ZFP36 based on the NicheNet method (Figure S12A).^51^ The cytotoxic effects of GZMA+ CTLs on MUC1+ cancer cells through GZMA‐F2RL1 axes strengthened after immunotherapy (Figure S13A–F). However, their crosstalk via TIGIT‐NECTIN2 interaction is also augmented (Figure S13G–J). Overall, GZMA+ CTL dysfunction may be attributed to the intimate interplay with C1Q+ TAMs via galectin and SPP1 pathways in the chemo‐ and immunoresistant groups, respectively.

### 3.8 Tracking the therapeutic‐driven developmental trajectory of neutrophils

Neutrophils are known for their plasticity and heterogeneity in the tumour microenvironment (TME),^52^ yet their phenotypes in the PME remained undetermined. Herein, we distinguished six distinct subpopulations of neutrophils, each characterized by unique transcriptional profiles (Figure S14A). GSVA analyses further illustrated the Neu_01 (CLEC10A+) with profound AP activity, Neu_02 (VNN+) characterized by extracellular matrix remodelling capacity, Neu_04 (CMTM2+) with glycolytic function, the IFN‐related immunostimulatory Neu_06 (RSAD2), and pro‐inflammatory Neu_03 (CCL20+) and Neu_05 (CXCR3+) (Figure S14B).

The developmental track was determined to originate from Neu_03 and Neu_05, with Neu_02 acting as the transitioning phase and subsequently shifting towards the directions of Neu_04 or Neu_06 (Figure S14C). The expression level of CTSL and innate immune response activity gradually declined along the pseudotime path. Inversely, the expression levels of CMTM2, ISG15, and CXCL8 gradually elevated (Figure S14D). Correspondingly, pathway activities in glycolipid metabolism and pro‐angiogenesis/inflammation augmented (Figure S14E). Interestingly, more CMTM2+ neutrophil infiltrates were found in the therapeutic failure cases (Figure S14F), which also predicted an unfavourable prognosis (Figure S14G–J). Consequently, CMTM2+ neutrophils could be deemed as tumour‐associated neutrophils (TANs). Neutrophils and macrophages have been reported to synergistically foster an immunosuppressive milieu in the PT.^53^ Consistently, robust and positive relationships between their infiltrates were discerned in our cohort (Figure S14K), further corroborating in the TCGA‐STAD cohort (Figure S14L,M).

Generally, cellular state and functional transitions are concomitant with metabolic reprogramming.^54^ We noticed that CMTM2+ TANs manifested high expression levels of PFKFB3 (Figure S14N) and SLC25A5 (Figure S14O), which were key enzymes in the glycolysis pathway, further validated by the GSVA findings.^55^ Eventually, we observed that the CMTM2+ TANs‐macrophages interaction pathways were distinct between the chemo‐ (Figure S14Q) and immunoresistant (Figure S13R) cases, with annexin (ANXA1‐FPR1) and CCL (CCL3‐CCR1) pathway being dominant, respectively (Figure S14S). Briefly, terminally differentiated CMTM2+ TANs with high glycolytic activity contributed to chemoresistance.

### 3.9 Distinct DC‐mediated cellular interaction networks between chemo‐ and immunotherapy cohorts

**FIGURE 6:**
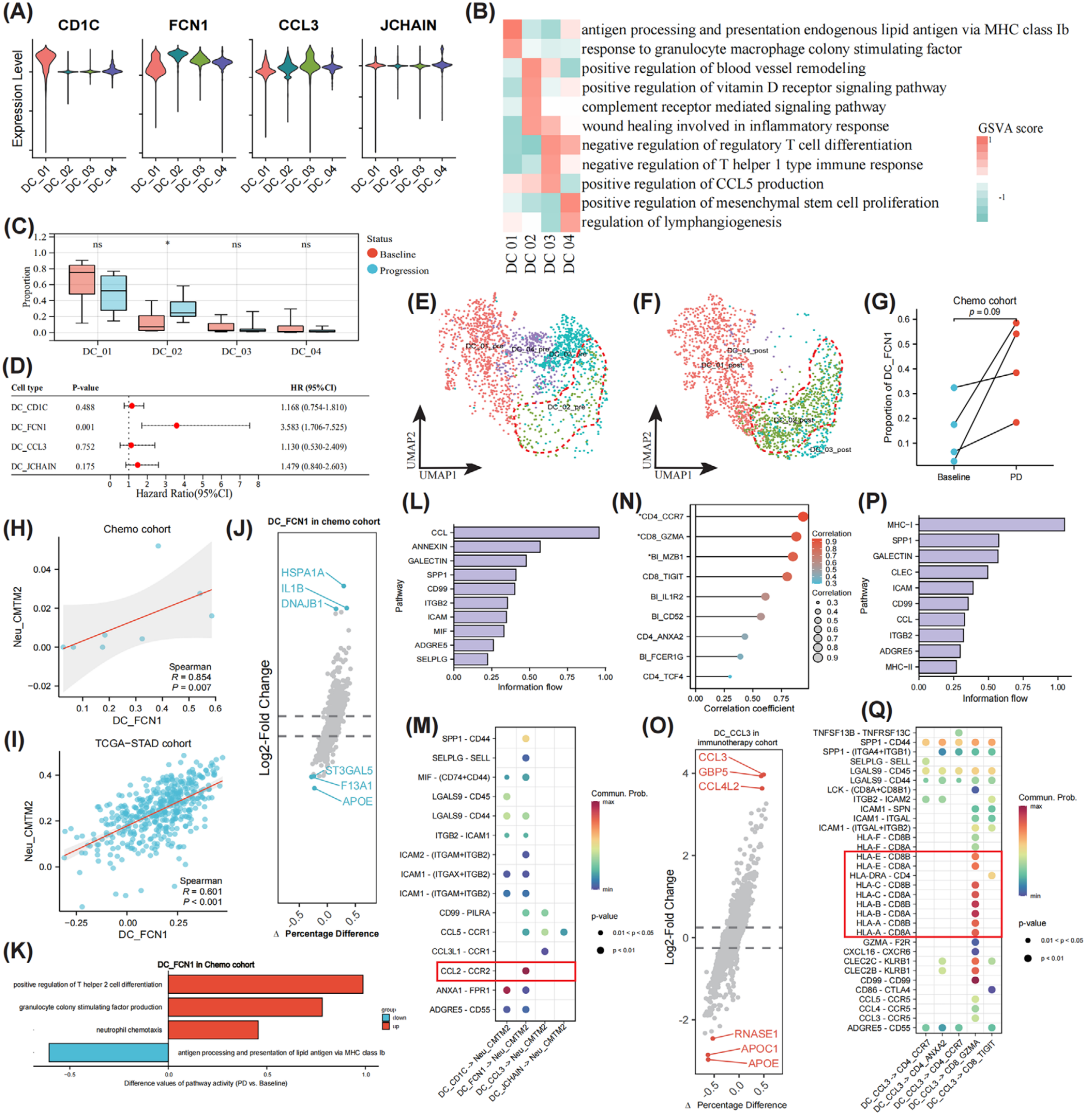
Profiling the dendritic cells (DC) in ascites microenvironment at single‐cell level. The violin plot depicts the highly expressed marker genes of each DC subset (A). Heatmap illustrates functional differences among different DC subtypes, with the intensity of colour showing gene set variation analysis scores (B). Box plot showing the proportion of changes in different DC subtypes in the progression compared with the baseline samples (C). Forest plot demonstrating the prognostic significance of different DC gene signatures in the TCGA‐STAD dataset (D). UMAP plots (E, F) and paired dot plots (G) visualize the fold change in the proportion FCN1+ DCs compared between pre‐ and post‐chemotherapy samples. Scatter plots indicate positive and strong associations between FCN1+ DCs and CMTM2+ neutrophils infiltrate in the immunotherapy (H) and TCGA‐STAD (I) cohorts. Volcano plot indicates the DEGs in FCN1+ DCs between pre‐ and post‐chemotherapy (J), along with associated functional changes (K). Summary of putative communications from different DC subsets to CMTM2+ neutrophils by the overall information flow (L) and the ligand–receptor pairs (M) in the chemotherapy cohort. Correlations between FCN1+ DCs and T‐ and B‐lymphocyte subsets in their cellular proportion in samples treated with immunotherapy (N). Volcano plot indicates the DEGs in CCL3+ DCs pre‐ and post‐immunotherapy (O). Summary of putative communications from CCL3+ DCs to different T‐lymphocyte subsets by the overall information flow (P) and the ligand–receptor pairs (Q) in the immunotherapy cohort. *p < .05; ns, non‐significant.

DCs constitute the leading regulators responsible for orchestrating antigen‐specific tumour immunity. We discovered four distinct DC subsets, characterized by high expression levels of CD1C (DC_01), FCN1 (DC_02), CCL3 (DC_03), and JCHAIN (DC_04), respectively (Figure 6A). The CD1C+, CCL3+, and JCHAIN+ DCs could be defined as type 2 conventional, inflammatory, and plasmacytoid DC upon GSVA findings, individually (Figure 6B).^56^

The differentiation route was delineated to originate from DC_01 and either maintain the initial state or transit towards DC_02/03/04 (Figure S11G). The expression level of CD1C exhibited an initial increase and subsequent decline, with genes with similar transcriptomic patterns being enriched into the AP pathway (Figure S11H). Expression intensities of chemokine genes like CCL5, CXCL8, and CXCR4, and inflammation‐related gene NFKB1A elevated gradually, and relevant genes were enriched into myeloid cell differentiation and inflammatory response (Figure S11I). Significantly, amplification of FCN1+ DC was detected in treatment resistance cases (Figure 6C), which also acted as a predictor of dismal prognosis (Figure 6D). An increasing infiltration trend of FCN1+ DCs was also observed in the chemotherapy group (Figure 6E–G).

Notably, positive associations between FCN1+ DC and CMTM2+ TAN infiltrates were identified (Figure 6H), which remained robust in the TCGA‐STAD cohort (Figure 6I). Significant upregulation of IL1β, a major proinflammatory factor, in FCN1+ DC, was highlighted in the chemoresistant cases (Figure 6J).^57^ Accordingly, FCN1+ DCs displayed enrichment of granulocyte colony‐stimulating factor production and neutrophil chemotaxis pathways (Figure 6K). Inversely, the canonical AP function of DCs attenuated. CellChat algorithm further indicated that FCN1+ DCs may secrete CCL2 to recruit CMTM2+ TANs (Figure 6L,M).

Strikingly, robust and positive correlation between CCL3+ DCs and major effector cells like CCR7+ and GZMA+ T‐lymphocytes and MZB1+ B‐lymphocytes were discovered after immunotherapy (Figure 6N). DEG analysis further indicated the markedly up‐regulated expression levels of CCL3 and GBP5, which may prime immune infiltration (Figure 6O).^58^, ^59^ CellChat algorithm additionally showed that CCL3+ DCs could present antigen to GZMA+ CTL via MHC molecules, thereby facilitating antitumour immunity (Figure 6P,Q). Taken together, FCN1+ DCs could chemoattract CMTM2+ TANs to formulate a proinflammatory PME in the chemoresistant cases synergistically. Inversely, immunotherapy may drive CCL3+ DCs to prime CTL activation via AP.

### 3.10 Intimate interplay between CXCL2+ fibroblasts and C1Q+ TAMs

The fibroblast population was categorized into four heterogeneous subsets upon their expression profiles, including the CXCL2+, LCN2+, VIM+, and CLIC3+ ones, respectively (Figure S15A). They showed potential in immunomodulatory, extracellular matrix remodelling, pro‐angiogenesis, and actin filament formation in PME, individually (Figure S15B). Monocle algorithm inferred that the LCN2+ subtype was the root of differentiation, and the CXCL2+ and VIM+ subtypes were in the end‐point state (Figure S15C). The expression levels of matrix remodelling‐related genes like MXRA5, COLIA2, and IGF1 showed a steady increase along the pseudotime trajectory (Figure S15D), with enrichment observed in the extracellular structure organization pathway (Figure S15E). Additionally, higher pro‐angiogenic activity was observed in the terminal differentiation stage, suggesting a shift towards cancer‐associated fibroblasts (CAFs).^60^ Remarkably, higher infiltrates of terminally differentiated fibroblast subsets all correlated with worse OS in GC patients (Figure S15F,G).

Reciprocal communications between fibroblasts and macrophages in the TME are crucial for the formation of an immunosuppressive milieu that restrains T‐lymphocyte activation.^61^ Intriguingly, a positive and strong relationship was only identified between C1Q+ TAMs and CXCL2+ CAFs (Figure S15H), which remained robust in the external TCGA‐STAD cohort (Figure S15I). Moreover, SAA1 and PLAG2A, which correlated with the pro‐angiogenic function of CAFs, demonstrated a pronounced elevation in the chemoresistant cases (Figure S15J).^62^, ^63^ GSVA findings further underscored the enrichment in blood vessel remodelling and complement activation pathways (Figure S15K,L). As expected, the complement pathway was dominant across all information flow mediating CXCL2+ CAFs‐C1Q+ TAMs crosstalk, particularly through the C3‐C3AR1/(ITGAM+ITGB2) axes (Figure S15M,N). NicheNet approach further unveiled that CXCL2+ CAFs displayed a potent regulatory effect towards C1Q+ TAMs, specifically targeting the pro‐angiogenic gene VEGFA (Figure S12B).

Additionally, CAFs have been shown to bolster the invasive capability of tumour cells in TME,^64^ yet the persistence of such effects in the PME remains unclear. Remarkably, robust and strong associations between VIM+ CAFs and MUC1+ cancer cell infiltration were found in both our chemotherapy cohort (Figure S15O) and TCGA‐STAD dataset (Figure S15P). Furthermore, saliently enhanced expression levels of MMP7 and SDC1, which correlated with extracellular matrix remodelling,^65^, ^66^ in MUC1+ cancer cells within the chemotherapy cohort was discovered (Figure S15Q), further supported by the GSVA findings (Figure S15R). The Cellchat algorithm further revealed the MDK‐SDC1/4 axes dominated the crosstalk between VIM+ CAFs and MUC1+ cancer cells (Figure S15S–U).

Collectively, CXCL2+ CAFs may stimulate the pro‐angiogenic capacity of C1Q+ TAMs via the complement activation pathway, thus contributing to chemoresistance. On the other hand, VIM+ CAFs may facilitate the EMT function of MUC1+ cancer cells through the MK pathway.

### 3.11 FCER1G+ B‐lymphocyte may blunt the activation of T‐lymphocytes by TGF‐β

We then concentrated on unique classes of B‐lymphocytes, resulting in four distinct subclusters: the MZB1+ subcluster (Bl_01) was featured by immunoglobulin production, the immunostimulatory CD52+ subcluster (Bl_02), the FCER1G+ (Bl_03) subtype with TGF‐β activation capacity, and the pro‐inflammatory IL1R2+ (Bl_04) subset (Figure S16A,B). Monocle algorithm postulated that the Bl_02 served as the root of trajectory, branching into either Bl_03 or Bl_01 (Figure S16C). Notably, the expression levels of immunoglobulin‐related genes, such as JCHAIN, IGHG4, and IGKC, gradually enhanced along the pseudotime route (Figure S16D). Moreover, TNFRSF17, which serves as the B‐lymphocyte maturation antigen, also exhibited marked upregulation in the terminal state.^67^ Enrichment in regulating adaptive immune response pathways via immunoglobulin or cytokine production was also discovered (Figure S16E). Furthermore, high Bl_03 and Bl_04 infiltrate correlated with a trend of unfavourable OS (Figure S16F,G).

Strikingly, strong negative associations between FCER1G+ B‐lymphocytes and GZMA+ (Figure S16H), CCR7+ (Figure S16I), and TCF4+ T‐lymphocytes (Figure S16J) in the chemoresistant cohort were uncovered. We were then interested in interrogating how FCER1G+ B‐lymphocytes may impede T‐lymphocyte infiltration. Significantly up‐regulated expression intensities of immunoglobulin‐related genes like IGKC and IGLC2 of FCER1G+ B‐lymphocytes were recognized (Figure S16K). Additionally, their pathway activities in modulating IFN‐mediated signalling attenuated, whereas in producing TGF‐β elevated (Figure S16L). Expression degrees of TGF‐β1 (Figure S16M) and IL‐10 (Figure S16N), two major immunosuppressive cytokines,^68^, ^69^ were also shown to highly express in FCER1G+ B‐lymphocytes of chemoresistant cases. Moreover, FCER1G+ B‐lymphocytes exhibited the highest TBRS scores (Figure S16O). Finally, the iTALK algorithm unveiled that FCER1G+ B‐lymphocytes may release TGF‐β1 to exert extensive immunosuppressive effects on various T‐lymphocyte subsets (Figure S16P). Overall, FCER1G+ B‐lymphocyte may blunt the activation of T‐lymphocytes via TGF‐β, potentially contributing to treatment failure.

### 3.12 Cellular interaction networks within PME in the immunoresistance cohort

**FIGURE 7:**
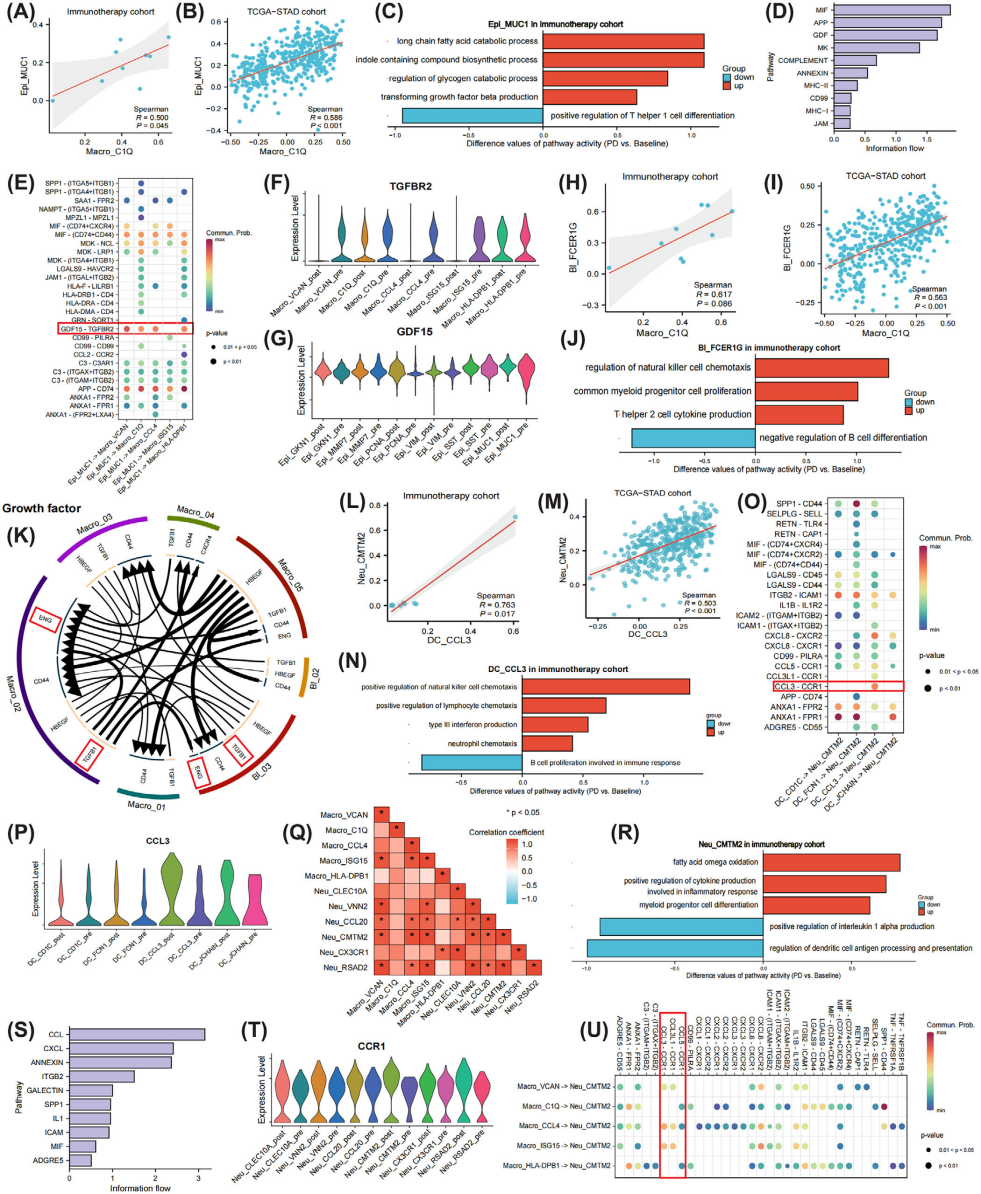
Characteristics and dynamics of key cell subsets in malignant ascites microenvironment of immunoresistant cases. Strong numerical associations between C1Q+ macrophages and MUC1+ malignant cells in immunotherapy cohort (A), validated in the TCGA‐STAD cohort (B). Functional transitions of MUC1+ malignant cells in progression than baseline samples (C). The overall signalling pathway with MUC1+ malignant cells as signal transmitters (D) and their interaction probabilities with macrophages mediated by ligand–receptor pairs (E). The expression profiles of TGFBR2 and GDF15 among different macrophage (F) and epithelial cell (G) subsets, respectively. Consistent strong infiltration relationships between C1Q+ macrophages and FCER1G+ B‐lymphocytes in the immunotherapy (H) and TCGA‐STAD (I) cohorts. Immunomodulatory function changes of FCER1G+ B‐lymphocytes in the immunotherapy cohort (J). Circos plots representing the top ligand–receptor interactions between macrophage and B‐lymphocyte in the growth factor module (K). Positive infiltration associations between CMTM2+ neutrophils and CCL3+ dendritic cells (DC) in the immunotherapy (L) and TCGA‐STAD (M) cohorts. Immune‐regulatory function alterations of CCL3+ DCs in progression than baseline samples (N). The interaction probabilities between different DC subtypes and CMTM2+ neutrophils (O). The expression profiles of CCL3 among different DC subtypes (P). Heatmap illustrates the intimate relationships among different neutrophil and macrophage subtypes (Q). Functional changes of CMTM2+ neutrophils in progression versus baseline samples (R). The overall signalling pathway with CMTM2+ neutrophils as signal receivers (S) and their interaction probabilities with macrophages (U). The expression spectrums of CCR1 among different neutrophil subtypes (T). *p < .05.

A co‐infiltrated pattern between C1Q+ TAMs and MUC1+ tumour cells was discovered (Figure 7A) and found to be consistent in the external cohort (Figure 7B). Furthermore, MUC1+ tumour cells showed enhanced pathway activities in lipid metabolism and TGF‐β production (Figure 7C). Moreover, MUC1+ tumour cells may release GDF15 to affect C1Q+ TAMs, possibly priming their polarization towards a pro‐tumour (i.e., M2) phenotype (Figure 7D–G).^70^ Higher C1Q+ TAM infiltration was also accompanied by an increasing trend of FCER1G+ B‐lymphocyte infiltrates (Figure 7H,I). Functional analysis further indicated higher activity in T helper 2 cell cytokines like IL‐10 and IL4 production of FCER1G+ B‐lymphocytes (Figure 7J). Eventually, reciprocal interactions between C1Q+ TAMs and FCER1G+ B‐lymphocytes through TGF‐β1‐ENG(CD105) axes were unveiled by the iTALK method, which has been shown to potentiate angiogenesis in various cancers (Figure 7K).^71^

A strong positive relationship between CCL3+ DCs and CMTM2+ TANs infiltration was observed (Figure 7L,M). CCL3+ DCs exhibited enhanced pathway activities in IFN production and facilitated the recruitment of lymphocytes and neutrophils after immunotherapy (Figure 7N). Intriguingly, the intimate interplay between CCL3+ DCs and CMTM2+ TANs through CCL3‐CCR1 pairs was observed (Figure 7O,P). Likewise, macrophages may also release CCL3 to recruit CMTM2+ TANs, which was postulated to promote tumour progression cooperatively (Figure 7Q–U).^72^, ^73^

**FIGURE 8:**
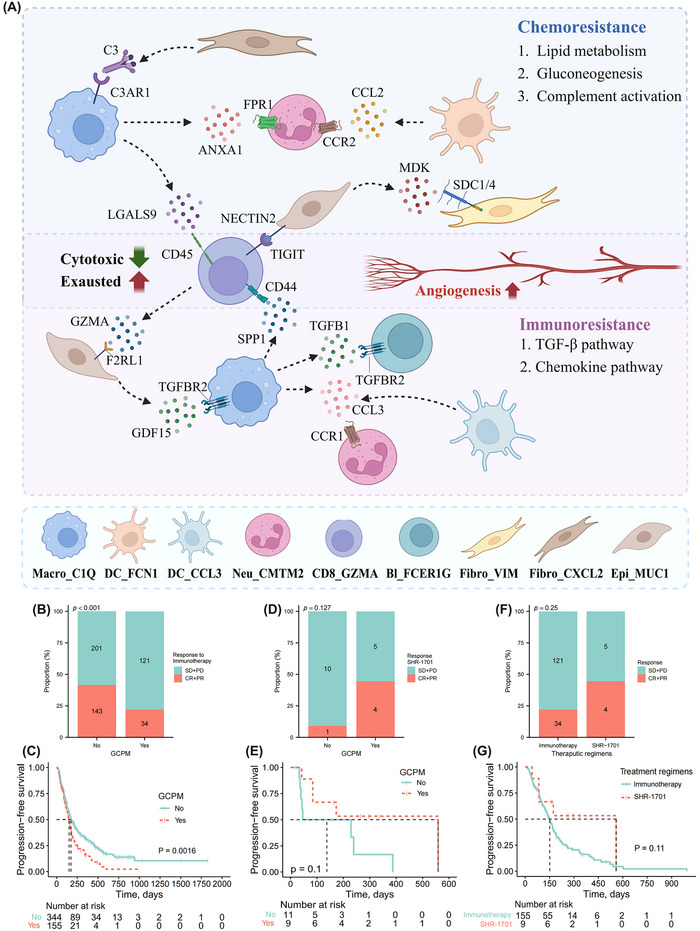
Summary of cellular dynamics within malignant ascites microenvironment in gastric cancer with chemo/immunoresistance. The common features of chemo‐ and immune‐resistance involve the attenuated cytotoxic effect of CD8+ T‐lymphocytes and augmented pro‐angiogenesis function of macrophages. Beyond these similarities, chemoresistance and immunoresistance cases exhibit distinct characteristics. In chemoresistance cases, C1Q+ macrophages exhibit higher lipid metabolism activity and may suppress the activation of GZMA+ T‐lymphocytes through LGALS9‐CD45 interactions. C1Q+ macrophages could also recruit tumour‐associated CXCL2+ fibroblasts and CMTM2+ neutrophils via complement and annexin pathways. MUC1+ malignant cells, with high glycolytic activity, may drive the exhaustion of GZMA+ T‐lymphocytes by NECTIN2‐TIGIT crosstalk. In immunoresistance cases, C1Q+ macrophages and FCER1G+ B‐lymphocytes may crosstalk through the TGF‐β pathway. C1Q+ macrophages and CCL3+ dendritic cells could both secret CCL3 to attract the tumour‐associated CMTM2+ neutrophils. The MUC1+ malignant cells may secret GDF15 to induce the immunosuppressive function of C1Q+ macrophages, which subsequently affect GZMA+ T‐lymphocyte functions by SPP1‐CD44 interaction. Overall, significant metabolic reprogramming and complement activation, and TGF‐β pathway activation are found in the chemoresistance and immunoresistance cases, respectively (A). The impact of gastric cancer peritoneal metastasis (GCPM) on the treatment response and immune‐related progression‐free survival (irPFS) in the PKUCHIO (B, C) and SHR‐1701 (D, E) cohorts. Comparison of treatment efficacy (F) and irPFS (G) of GCPM cases in the SHR‐1701 and PKUCHIO cohorts. CCL, chemokine ligand; TGF, transforming growth factor; GDF, growth differentiation factor. GCPM, gastric cancer peritoneal metastasis.

To summarize, distinct drug resistance patterns and treatment‐driven temporal dynamics of cellular interaction networks within PME were discovered. Generally, significant metabolic reprogramming and complement activation, and TGF‐β pathway activation were unveiled in the chemoresistance and immunoresistance cases, respectively (Figure 8A).

### 3.13 Validation of immunoresistant characteristics by post hoc analysis of clinical trial

Reasonably, patients with GCPM were found to be less likely to benefit from immunotherapy (21.9% vs. 41.6%, *p* < .001) (Figure 8B) and manifested significantly shorter irPFS (median irPFS: 151d vs. 174d, *p* = .002) (Figure 8C) than those without in the PKUCHIO cohort. As mentioned above, the highly activated TGF‐β pathway within PME was the major feature in the immunoresistant cohort. Then we wondered whether targeting the TGF‐β pathway may improve therapeutic efficacy in patients with GCPM. Consequently, we retrospectively extracted patients’ clinicopathologic features in the SHR‐1701 and PKUCHIO cohort and conducted post hoc analyses to investigate the associations between therapeutic effects and peritoneal metastasis status at the time of GC diagnosis. Surprisingly, a trend of higher response rate was found in patients with GCPM than those without (44.4% vs. 9.1%, *p* = .127) in the SHR‐1701 (dual anti‐PD‐L1/TGF‐βRII agent) cohort (Figure 8D). Moreover, SHR‐1701 seemed to yield irPFS benefits in GCPM cases (median irPFS: 559d vs. 46d, *p* = .119) (Figure 8E). Eventually, we interrogated whether a dual anti‐PD‐L1/TGF‐βRII regimen may yield greater benefits than sole anti‐PD‐1/PD‐L1 therapy in patients with GCPM. Intriguingly, a trend of higher response rate (44.4% vs. 21.9%, *p* = .25) (Figure 8F) and longer irPFS (median irPFS: 559d vs. 151d, *p* = .112) (Figure 8G) was observed in the SHR‐1701 cohort. Consequently, the potential of co‐targeting PD‐L1 and TGF‐β pathways within PME for improving prognosis in GCPM cases was demonstrated, surpassing the efficacy of solely blocking the PD‐1/PD‐L1 pathway.

### 3.14 Potential drugs targeting major cell populations involved in treatment resistance

Given the putative pivotal roles of C1Q+ TAMs, CMTM2+ TANs, and MUC1+ cancer cells in mediating treatment resistance, the DREIMT tool was used to prioritize the potential drugs targeting them (Figure S17A–F). After intersecting the predicted drug spectra, we found the tranilast, an anti‐allergic agent, may co‐target CMTM2+ TANs and MUC1+ ECs via modulating immune relevant pathways including TGF‐β, irrespective of chemoresistant or immunoresistant cases (Figure S17G).^74^ Moreover, chlorpromazine, one of the antipsychotic medications, was discovered to co‐target C1Q+ TAMs and MUC1+ ECs.^75^

## 4 DISCUSSION

In the present study, scRNA‐seq and ST approaches were utilized to thoroughly delineate the dynamic cellular differentiation trajectories and evolution driven by either chemotherapy or immunotherapy in GCPM cases.

Firstly, dynamic changes in cellular composition, functions, and interaction networks among different GCPM‐related sites were deciphered. Notably, we observed a decline in T‐lymphocyte infiltrates, accompanied by their increasingly exhausted state from PT to ascites and PM. Interestingly, the higher pro‐angiogenic capacity of macrophages was observed in the ascites compared with PT or PM, suggesting their crucial role in driving cancer cell metastasis within PME.^73^ Notably, the highest EMT function of ECs was discerned in the PT. ST analyses further indicated that ECs tended to distribute at the IM in the PT, wherein they co‐localized and closely interacted with fibroblasts via the MDK pathway. There is also evidence that MDK serves as a mediator facilitating the acquisition of pivotal cancer hallmarks including metastasis, representing a potential treatment target.^76^

It is widely recognized that the plasticity and heterogeneity of EC evolution contribute to cancer development and treatment inefficacy. Consequently, we then traced the differentiation route of ECs during GCPM. We observed that ECs evolved from a high‐proliferative status to a high‐metastatic status. The MUC1+ cancer cells, with the highest EMT capacity among all EC subsets, were identified as the terminally differentiated state, which also portended a dismal prognosis. Meanwhile, the abnormal glycosylation and overexpression of MUC1 have been reported in various epithelial cancers, potentially suppressing the expression of E‐cadherin while inducing snail and vimentin expressions to bolster EMT.^77^

The peritoneum is the most frequent site of metastasis and relapse in GC patients, which may not only cause bowel obstruction, the development of massive amounts of malignant ascites, and treatment resistance, invariably leading to death. During the 20th century, GCPM was deemed as treatment futility because chemotherapy and surgical treatments could rarely improve symptom management and survival. In recent years, novel treatment strategies like cytoreductive surgery (CRS) and hyperthermic intraperitoneal chemotherapy (HIPEC) have brought significant therapeutic advances for GCPM, improving median OS for around 5 months.^78^, ^79^

Moreover, the therapeutic effects of CRS seem to be better when combined with HIPEC than without. A recent meta‐analysis incorporating 620 patients demonstrated that the median OS improved by 4 months in cases treated by the combination of CRS and HIPEC than CRS alone.^80^ However, a recent phase III randomized controlled trial reported that CRS plus HIPEC did not improve OS than CRS alone.^81^ Despite the encouraging treatment improvements, CRS and HIPEC have not yet been established as the standard clinical therapeutic protocols for GCPM, mainly attributed to the controversial findings among studies and the lack of high‐quality studies. Moreover, the therapeutic potential of immunotherapy, either alone or in combination with CRS and HIPEC, for treating GCPM remains to be fully explored.^82^

Current treatment options for patients with GCPM are limited and the therapeutic efficacy remains far from satisfactory, primarily due to the intricate and heterogeneous PME. Leveraging the advantages of paired and long‐term follow‐up ascites samples, we could comprehensively delineate the therapeutic‐driven cellular remodelling within PME. As the dominant cell type within PME, macrophages played an indispensable role in mediating therapeutic resistance. First, a significant expansion of VCAN+ macrophages with highly pro‐angiogenic function was found in both chemo‐ and immunoresistance cohorts. Meanwhile, such angiogenesis‐related VCAN+ macrophage subset was also identified in breast cancer and melanoma.^83^ Secondly, the terminally differentiated C1Q+ TAMs with elevated lipid metabolism activity played a pivotal role in facilitating treatment failure. C1Q+ TAMs may hinder the infiltration of GZMA+ CTLs, the crucial cytotoxic cell type, into the PME. Concurrently, C1Q+ TAMs may blunt the activation and induce the exhaustion of GZMA+ CTLs by LGALS9‐CD45 and SPP1‐CD44 pathways in chemo‐ and immunoresistance groups, respectively, potentially through targeting the key transcription factor ZFP36 in T‐lymphocyte lineage commitment. Importantly, LGALS9 is the ligand of TIM‐3, an inhibitory receptor that is expressed along with T‐cell exhaustion, and their interaction can modulate T‐cell differentiation.^43^ Overexpression of LGALS9 has been reported in distinct cancer types, and targeting LGALS9 may restore antitumour immunity.^50^ Besides, the SPP1‐CD44 axis is the widely recognized crosstalk pathway between TAMs and cancer cells or T cells, mainly involved in immune distortion and escape.^36^, ^84^ Consequently, targeting LGALS9 and SPP1 within the microenvironment represents promising targets for remodelling anti‐tumour immunity.

The complement system is a pivotal element of the innate immune response, and its inappropriate activation significantly impacts tumorigenic inflammation.^85^ More importantly, C1Q+ TAMs have emerged as a unique macrophage subset that orchestrates the immunosuppressive TME to drive tumour progression.^86^ Accordingly, we found that CXCL2+ CAFs may secrete complement C3 to interact with C1Q+ TAMs, mainly targeting the pro‐angiogenic gene VEGFA to maintain their M2‐like phenotype in chemoresistant tumours. Likewise, our team recently reported that C1Q+ TAMs within PME may induce PD‐L1‐mediated immune escape of cancer cells.^87^ Zhang et al. also found that C1Q+ TAMs with significant fatty acid metabolic reprogramming could hinder the anti‐tumour activity of CTLs in malignant pleural effusion.^88^

Targeting the key components within the complement system, such as the C1 and the potent anaphylatoxins C5a, has demonstrated anticancer prospects. For instance, researchers found that combined radiotherapy and C1‐inhibitor therapy improved the efficacy of suppressing glioblastoma growth.^89^ Ding and colleagues reported that C5aR1 antagonist reduced the abundance of MDSCs in colon tumours, whereas increasing CD8+ T‐cell infiltrates, thereby significantly inhibiting tumour development.^90^ Furthermore, the ongoing clinical trial evaluating the combination of anti‐PD‐L1 antibody Durvalumab and Avdoralimab, the antagonist of C5aR1 on MDSCs, holds promise for advanced solid tumour therapy (NCT03665129). Overall, more studies are needed to understand the intricate crosstalk between the complement system and cancer. Balancing the “benign” and “harmful” complement activation and exploiting them represent substantial prospects in cancer treatment.

Neutrophils constituted the primary cellular component in ascites and represented the inflammatory levels within PME.^91^ CMTM2+ TANs were regarded as the terminally differentiated state induced by chemo‐/immunotherapy. They underwent significant metabolic remodelling with high glycolytic activity, demonstrated a trend of expansion in the chemoresistance cohort, and correlated with worse prognosis. The metabolic switch of TANs into favouring glycolysis and fatty acid metabolism has been shown to facilitate the formation of an immunosuppressive milieu and contribute to drug resistance.^54^ Intriguingly, we found that C1Q+ TAMs may recruit CMTM2+ TANs via the chemokine pathway, potentially promoting tumour progression synergistically.

In the immunoresistant cohort, significant activation of TGF‐β pathways was noticed. Specifically, the MUC1+ cancer cells exhibited high TGF‐β production activity and were inclined to co‐infiltrate with C1Q+ TAMs. Besides, they may communicate through the GDF15‐TGF‐βR2 axis, and the activation of the TGF‐β pathway has been reported to prime macrophages polarizing towards the M2 phenotype.^92^ Additionally, reciprocal interactions between C1Q+ TAMs and FCER1G+ B‐lymphocytes via the TGF‐β pathway were found, further leading to the inhibitory effects on T‐lymphocytes. Considering such unique characteristics within PME, we were then interested in investigating whether dually blocking TGF‐β and PD‐L1 pathways may deliver greater clinical benefits than solely targeting the PD1/PD‐L1 pathway in patients with GCPM at the time of diagnosis. Surprisingly, through post hoc analyses of two in‐house cohorts, a trend of higher response rate and longer irPFS was observed, preliminarily supporting our hypothesis.

Indeed, the TGF‐β pathway has been widely acknowledged for its intensive involvement in tumorigenesis and progression. Therapeutic agents that target the TGF‐β pathway, either alone or in combination with other tumour‐promoting pathways such as PD‐L1^93^ and VEGF^94^ within TME have illustrated encouraging efficacy, albeit most remained at pre‐clinical and phase 1 trial status. Likewise, immune profiling of PM underscored the mesenchymal subtype with a considerable expression of TGF‐β, thereby raising the question of whether targeting the TGF‐β pathway in PME may restore anti‐tumour immunity and better manage GCPM. However, relevant studies were scarce, necessitating further interrogation.

To sum up, chemoresistant and immunoresistant cohorts shared common features including the presence of exhausted CTLs and pro‐angiogenic macrophages within PME. Except for these similarities, distinct characteristics such as significant complement system activation in chemoresistance cases and TGF‐β pathway activation in immunoresistance cases were found. Given that sampling ascites by paracentesis presents a markedly simpler and safer alternative to tissue biopsy, and PME more closely resembles the characteristics within TME than peripheral blood for advanced GC patients, our study pointed towards dynamic monitoring tumour state by ascites aspiration rather than tissue biopsy for tailoring therapeutic regimens.

On the bright side, chemotherapy and immunotherapy conferred several advantages within PME. Firstly, higher AP capacity of major antigen‐presenting cells was found, possibly resulting from chemotherapy‐induced immunogenic cell death. Secondly, the cytotoxic effects of GZMA+ CTLs on MUC1+ cancer cells augmented after immunotherapy, albeit concurrently accompanied by their increasing exhaustion. Consequently, harnessing and managing the cell subsets with tumoricidal capabilities while simultaneously eliminating the subsets exhibiting tumour‐promoting abilities represent prospective research directions.

Several advantages of our study are worth highlighting. First, the collection of paired pre‐treatment baseline and post‐treatment progression ascites samples was challenging considering the complexity of treatment lines and adherence to follow‐up of GCPM patients. Consequently, our study provided valuable insights into the resistant features within PME of GCPM cases, identifying several potential targets for tailoring novel treatment strategies in the future. Secondly, the dynamic cellular differentiation trajectories during GCPM were comprehensively unveiled in single‐cell, protein, and spatial levels. Our findings supported the speculation that tumour cells and their surrounding milieu undergo co‐evolution within metastatic lesions, reflected by extensive alterations in cell compositions, functional states, and communication networks in metastases compared with their matched PTs. Third, the immunoresistant features identified through scRNA‐seq analyses were preliminarily validated by post hoc analyses from a phase 1 clinical trial and an in‐house retrospective cohort, providing a valuable reference for developing immunotherapy combination strategies.

Meanwhile, several limitations existed in our study. First, the sample size of cohort 1 was relatively small, especially for the matched PT and PM samples. Second, the discrimination of various cell types and cellular interaction networks inference remained in the exploratory and hypothetical stage, relying primarily on transcriptomic data. As such, future experimental validation in both in vitro and in vivo settings is imperative. Third, the sample size of the SHR‐1701 cohort was limited. Moreover, the nature of post‐hoc analysis design may yield inevitable biases. Additionally, we could not obtain the paired dynamic PT samples during pharmacotherapy because patients with malignant ascites were quite ill, and the acquisition of PT was clinically unjustifiable.

In conclusion, our study delineated distinct cellular differentiation trajectories as well as intricate interaction networks among different GCPM‐related tissues and identified crucial features associated with drug resistance. These findings may facilitate the exploration of effective targets for improved GCPM treatment.

## AUTHOR CONTRIBUTIONS

Haoxin Peng, Lei Jiang, Jiajia Yuan, Xiangrong Wu, Nan Chen, Dan Liu, Yueting Liang, Yi Xie, Keren Jia, Yanyan Li, Xujiao Feng, Jian Li, Xiaotian Zhang, Lin Shen, Yang Chen *Conception and design*: Haoxin Peng, Lei Jiang, Lin Shen, and Yang Chen. *Administrative support*: Lin Shen and Yang Chen. *Provision of study materials or patients*: All authors. *Data analysis and interpretation*: Haoxin Peng, Lei Jiang, Xiangrong Wu, Lin Shen, and Yang Chen. *Manuscript writing*: Haoxin Peng, Lei Jiang, and Yang Chen. *Final approval of manuscript*: All authors.

## CONFLICT OF INTEREST STATEMENT

The authors declare no conflict of interest.

## ETHICS STATEMENT AND CONSENT TO PARTICIPATE

Informed consent of included patients was gained, and this research was approved by the PKUCH Ethics Committee according to the Declaration of Helsinki. All authors provided their consent to publication.

## Supporting information



Supporting Information

Supporting Information

Supporting Information

Supporting Information

Supporting Information

Supporting Information

Supporting Information

Supporting Information

Supporting Information

Supporting Information

## Data Availability

Data used to support the findings of this study are available from the corresponding authors upon reasonable request.
