# Supplementary material for: Single‐cell characterization of differentiation trajectories and drug resistance features in gastric cancer with peritoneal metastasis
Source: Clin Transl Med. 2024 Oct 18;14(10):e70054. doi: 10.1002/ctm2.70054 (PMC11488346; doi:10.1002/ctm2.70054)
Supplement: Supplementary file 1 — Supporting Information [file CTM2-14-e70054-s007.docx]

**Supplementary table 1.** Gene signatures for assessment of cellular function by single-sample enrichment analysis.

| **Function** | **Gene sets** |
| --- | --- |
| Cytotoxic | NKG7, PRF1, IFNG, GNLY, GZMK, GZMA, GZMB |
| Inflammation-promoting | CCL5, CD19, CD8B, CXCL10, CXCL13, CXCL9, GNLY GZMB, IFNG, IL12A, IL12B, IRF1, PRF1, STAT1, TBX21 |
| HLA (antigen presentation) | HLA-E, HLA-DPB2, HLA-C, HLA-J, HLA-DQB1, HLA-DQB2, HLA-DQA2, HLA-DQA1 HLA-DOA, HLA-DPB1, HLA-DRA, HLA-DRB6, HLA-L, HLA-F, HLA-G, HLA-DMB HLA-DMA, HLA-DOB, HLA-DRB1, HLA-H, HLA-B, HLA-DRB5, HLA-A, HLA-DPA1 |
| Angiogenesis | CCND2, CCNE1, CD44, CXCR4, E2F3, EDN1, EZH2, FGF18 FGFR1, FYN, HEY1, ITGAV, JAG1, JAG2, MMP9, NOTCH1 PDGFA, PTK2, SPP1, STC1, TNFAIP6, TYMP, VAV2, VCAN, VEGFA |
| Proliferative | MCM5, MCM2, MCM4, MCM6, MKI67 |
| Exhausted | LAG3, TIGIT, PCCD1, HAVCR2, CTLA4, LAYN, ENTPD1 |
| M1_signature | CD86, KYNU, FCGR1A, IRF5, CD40, CXCL11, IDO1, IL23A, IL12A, NOS2, CCR7, IL1A, CXCL9, IL6, CD80, CXCL10, TNF, IRF1, CCL5, IL1B |
| M2_signature | CD276, FN1, MRC1, CCL13, CCL18, LYVE1, PDCD1LG2, MMP9, TGFB2, ARG2 |
| Epithelial-to-mesenchymal transition | ABI3BP, ACTA2, ADAM12, ANPEP, APLP1, AREG, BASP1, BDNF, BGN, BMP1, CADM1, CALD1, CALU, CAP2, CAPG, CD44, CD59, CDH11, CDH2, CDH6, COL11A1, COL12A1, COL16A1, COL1A1, COL1A2, COL3A1, COL4A1, COL4A2, COL5A1, COL5A2, COL5A3, COL6A2, COL6A3, COL7A1, COL8A2, COMP, COPA, CRLF1, CTGF, CTHRC1, CXCL1, CXCL12, CXCL6, CYR61, DAB2, DCN, DKK1, DPYSL3, DST, ECM1, ECM2, EDIL3, EFEMP2, ELN, EMP3, ENO2, FAP, FAS, FBLN1, FBLN2, FBLN5, FBN1, FBN2, FERMT2, FGF2, FLNA, FMOD, FN1, FOXC2, FSTL1, FSTL3, FUCA1, FZD8, GADD45A, GADD45B, GAS1, GEM, GJA1, GLIPR1, GLT25D1, GPC1, GPX7, GREM1, HTRA1, ID2, IGFBP2, IGFBP3, IGFBP4, IL15, IL32, IL6, IL8, INHBA, ITGA2, ITGA5, ITGAV, ITGB1, ITGB3, ITGB5, JUN, LAMA1, LAMA2, LAMA3, LAMC1, LAMC2, LEPRE1, LGALS1, LOX, LOXL1, LOXL2, LRP1, LRRC15, LUM, MAGEE1, MATN2, MATN3, MCM7, MEST, MFAP5, MGP, MMP1, MMP14, MMP2, MMP3, MSX1, MXRA5, MYL9, MYLK, NID2, NNMT, NOTCH2, NT5E, NTM, OXTR, PCOLCE, PCOLCE2, PDGFRB, PDLIM4, PFN2, PLAUR, PLOD1, PLOD2, PLOD3, PMEPA1, PMP22, POSTN, PPIB, PRRX1, PRSS2, PTHLH, PTX3, PVR, QSOX1, RGS4, RHOB, SAT1, SCG2, SDC1, SDC4, SERPINE1, SERPINE2, SERPINH1, SFRP1, SFRP4, SGCB, SGCD, SGCG, SLC6A8, SLIT2, SLIT3, SNAI2, SNTB1, SPARC, SPOCK1, SPP1, TAGLN, TFPI2, TGFB1, TGFBI, TGFBR3, TGM2, THBS1, THBS2, THY1, TIMP1, TIMP3, TNC, TNFAIP3, TNFRSF11B, TNFRSF12A, TPM1, TPM2, TPM4, VCAM1, VCAN, VEGFA, VEGFC, VIM, WIPF1, WNT5A |
| TGF-β response | ABTB2, ACSBG1, ADCK2, ADRB2, AGTR2, AGXT2L1, AHI1, ALOX5AP, AMIGO2, ANGEL2, ANGPTL4, ANK1, ARFGAP1, ARHGAP12, ARID5B, ARL6IP2, ATF7IP, AVPI1, BET1L, BHLHB2, BHMT, BMPR2, BRDT, C13orf15, C18orf25, C3orf28, C3orf52, C6orf145, C6orf148, CCDC93, CD163, CD1E, CD28, CDKN1A, CDKN2AIP, CEBPD, CENPF, CITED2, CKMT2, COL1A1, COL4A1, COL4A2, COL8A2, CTGF, CUBN, CYBB, DDIT4, DNAJC7, DOPEY1, EDN1, ELK3, ETS2, FAT4, FGB, FHL3, FILIP1L, FLJ10357, FLT4, FLVCR2, FNDC3B, FSTL3, FZR1, GADD45B, GRB10, HLX, HMOX1, HNMT, HRH1, ID1, IL11, IL5, IRS1, JAG1, JMJD3, JUN, JUNB, LARP6, LBH, LEMD3, LMCD1, MAP3K4, MAS1, MGC14376, MLXIP, MTMR1, MYBL1, MYC, MYH11, NA, NCOR2, NDST1, NEDD9, NP, NPAS1, NR2F2, OLIG2, PAIP2B, PASK, PCTK2, PDGFA, PDLIM4, PFKFB3, PHLDB1, PKIA, PLK3, PNPLA4, PPP1R13L, PSCD1, PTH, PVRIG, RAB11FIP4, RAI2, RARA, RASL10A, RBMS1, RHOB, RNASE4, SERPINE1, SERTAD2, SGK, SKIL, SLC16A3, SLC17A3, SMAD7, SMOX, SMTN, SMURF1, SNAI1, SPHK1, SPP1, SPSB1, SSBP3, SYCP1, TBC1D2B, TBL1Y, TBPL1, TFEB, THPO, TMEPAI, TNFAIP8, TNFRSF12A, TPM1, TUBB4, TUFT1, UTP14A, VEGFA, YIPF5, ZEB1, ZFP36L1, ZNF318, ZNF395, ZNF44 |
