# Supplementary material for: Single‐cell characterization of differentiation trajectories and drug resistance features in gastric cancer with peritoneal metastasis
Source: Clin Transl Med. 2024 Oct 18;14(10):e70054. doi: 10.1002/ctm2.70054 (PMC11488346; doi:10.1002/ctm2.70054)
Supplement: Supplementary file 2 — Supporting Information [file CTM2-14-e70054-s002.docx]

**Supplementary table 2.** Gene signatures for different cell subclusters based on single-cell RNA sequencing.

| Cell subclusters | Gene signatures |
| --- | --- |
| DC_01 | HLA-DQA1, HLA-DPB1, NAPSB, CD1C, PKIB, HLA-DQA2, HLA-DRA, CD74, HLA-DPA1, CLEC10A |
| DC_02 | FCN1, APOBEC3A, VCAN, SLC39A8, MCEMP1, IL1R, C5AR1, THBS1, CCL5, NAMPT |
| DC_03 | CCL20, CXCL1, CTSL, RNASE1, SOD2, CTSD, CCL4, FCGR3A, FN1, CCL4L2 |
| DC_04 | IGKC, FCMR, TMEM156, PIM2, IGLC3, JCHAIN, CD69, CXCR4, LTB, BIRC3 |
| Neu_01 | VSIG4, HSP90B1, HLA-DRB5, HLA-DPB1, HLA-DPA1, CCR2, NPM1, SDF2L1, RETN, CLEC10A |
| Neu_02 | IL1R2, S100A9, S100A8, S100A12, VCAN, VNN2, CYP1B1, LYZ, CXCL8, SELL |
| Neu_03 | CTSL, THBS1, CD163, FABP5, SERPINB2, SLC39A8, CCL20, CCL2, PLIN2, PPIB |
| Neu_04 | CMTM2, KRT23, CEACAM1, DUSP1, MMP9, DUSP5, IL1R2, TIMP1, PIM2, VNN1 |
| Neu_05 | CX3CR1, EMP3, HLA-DRB5, FCGR3A, FBP1, L1TD1, TUBA1A, TIMP1, HLA-DPA1, SLC2A6 |
| Neu_06 | IFIT3, ISG15, MX1, RSAD2, CXCL10, CCL2, VAMP5, GBP1, CCL8, TNFSF10 |
| Bl_01 | MZB1, DERL3, XBP1, SDF2L1, SEC11C, LMAN1, ITM2C, CCND2, SSR4, SUB1 |
| Bl_02 | CD52, TRBC2, DUT, HOPX, TRAC, CCL5, CD2, STMN1, HMGB2, TMPO |
| Bl_03 | FCER1G, FCGR3A, FTL, IFI27, AIF1, MS4A4A, TYROBP, CCL3, CCL4, CCL2 |
| Bl_04 | IL1R2, SELENBP1, BCL2L15, SERPINB2, CCR7, THBS1, SELL, LGALS3, TNFAIP6, GYPC |
| Macro_01 | S100A12, VCAN, FCN1, IL1R2, CD52, APOBEC3A, ALOX5AP, RETN, LYZ, TIMP1 |
| Macro_02 | C1QB, C1QC, C1QA, IDH1, GPNMB, APOC1, APOE, FOLR2, LYVE1, SPP1 |
| Macro_03 | CCL4, CCL4L2, CCL20, CXCL8, CXCL3, CXCL1, CCL3, IL1B, CXCL2, IL6 |
| Macro_04 | CXCL10, CCL8, ISG15, MX1, TNFSF10, IFIT1, IFIT2, GBP1, GBP5, ISG20 |
| Macro_05 | HLA-DPB1, CD74, HLA-DRB1, HLA-DPA1, HLA-DRA, HLA-DRB5, HLA-DQA1, HLA-DQB1, HLA-DQA2, FCGR2B |
| Fibro_01 | CCDC80, COL3A1, SELENOP, LUM, CXCL6, CXCL1, IL6, CXCL10, CXCL2, CYR61 |
| Fibro_02 | LCN2, TSPAN8, S100P, EPCAM, SPINT2, PERP, GPRC5A, KRT18, CD55, C19orf33 |
| Fibro_03 | COL1A1, SPARC, COL1A2, COL3A1, DCN, COL5A1, VIM, POSTN, APOA1, CRABP2 |
| Fibro_04 | CLIC3, S100A3, COL5A1, THY1, CD248, FBLN2, PTGDS, IGF1, LDHB, MXRA8 |
| CD4_01 | CD3D, CCR7, MAL, FBXO32, RPLP0, PLAC8, IL6ST, SELL, IL7R, SELENOM |
| CD4_02 | CD3D, IFI27, ANXA2, DERL3, TPSAB1, SEC11C, MANF, SSR4, XBP1, FKBP11 |
| CD4_03 | CD3D, CD74, HLA-DRA, TCF4, ID3, ACP5, SELL, SPIB, CCDC50, CCR7 |
| CD8_01 | CCL5, NKG7, GZMA, GZMK, GZMH, CD8A, GZMB, KLRD1, GNLY, CD8B |
| CD8_02 | IL2RA, TIGIT, IL32, CTLA4, RGS1, FOXP3, SAT1, GBP5, SELL, ISG20 |
| Epi_01 | GKN1, GKN2, SCGB2A1, IGLC3, JCHAIN, C6orf58, GHRL, REG1A, MSMB, LIPF |
| Epi_02 | KRT17, ZG16B, MMP7, AGR2, LYZ, SCGB3A1 |
| Epi_03 | TUBA1B, H2AFZ, HMGA1, S100A10, KRT18, TUBB, PTTG1, HMGB2, CXCL3, MKI67 |
| Epi_04 | MUC1, C15orf48, IL1R2, DUOX2, MUC2, MUC4, CEACAM6, LGALS1, EMP3, MUC13 |
| Epi_05 | GCG, IL20RA, TTR, TM4SF4, OLFML3, DEPP1, SST, RBP4, HEPACAM2, ANXA13 |
| Epi_06 | VIM, CCL21, EMP3, LGALS1, MGP, TUBA1A, CCL2, SERPING1, PRG4, PDPN |

DC, dendritic cell; Neu, neutrophil; Bl, B-lymphocyte; Macro, macrophage; Fibro, fibroblast; Epi, epithelial cell.
