# Supplementary material for: Single‐cell characterization of differentiation trajectories and drug resistance features in gastric cancer with peritoneal metastasis
Source: Clin Transl Med. 2024 Oct 18;14(10):e70054. doi: 10.1002/ctm2.70054 (PMC11488346; doi:10.1002/ctm2.70054)
Supplement: Supplementary file 3 — Supporting Information [file CTM2-14-e70054-s005.docx]

**Supplementary table 3.** Sources and identifiers of antibodies used for multiplex immunofluorescence.

| **Antibody** | **Manufacturer** | **Identifier** | **Concentration** |
| --- | --- | --- | --- |
| FAP | abcam | ab207178 | 500X |
| CD31 | CST | CST3528 | 200X |
| CD68 | Zsbio | ZM0060 | 500X |
| CD3 | abcam | ab135372 | 300X |
| MUC1 | Genentech | GT208307 | 1X |
| PANCK | CST | 4545 | 500X |
