# Supplementary material for: Single‐cell characterization of differentiation trajectories and drug resistance features in gastric cancer with peritoneal metastasis
Source: Clin Transl Med. 2024 Oct 18;14(10):e70054. doi: 10.1002/ctm2.70054 (PMC11488346; doi:10.1002/ctm2.70054)
Supplement: Supplementary file 4 — Supporting Information [file CTM2-14-e70054-s008.docx]

**Supplementary table 4.** Baseline characteristics of the included cases in cohort 1.

| **Patient ID** | **Age** | **Gender** | **Lauren type** | **Treatment type** | **Treatment regimens** | **Follow-up days** |
| --- | --- | --- | --- | --- | --- | --- |
| 1 | 55 | Male | Intestinal | Chemotherapy | DDP + S-1 + TNF | 11 |
| 2 | 60 | Male | Mixed | Chemotherapy | SOX | 41 |
| 3 | 52 | Male | Intestinal | Chemotherapy | POS | 39 |
| 4 | 46 | Male | Diffuse | Chemotherapy | PTX | 59 |
| 5 | 42 | Male | Diffuse | Immunotherapy | PTX + S-1 + Camrelizumab + Apatinib | 181 |
| 6 | 61 | Female | Diffuse | Immunotherapy | Toripalimab + Sofantinib | 23 |
| 7 | 27 | Male | Mixed | Immunotherapy | Toripalimab + Sofantinib | 20 |
| 8 | 42 | Male | Intestinal | Immunotherapy | PTX + S-1 + PEM + Pembrolizumab | 64 |
| 9 | 41 | Female | Diffuse | Immunotherapy | SOX + Toripalimab | 58 |

DDP, cisplatin; PEM, pemetrexed; POS, paclitaxel + oxaliplatin + S-1; PTX, paclitaxel; S-1, Tegafur + Gimeracil + Oteracil; SOX, oxaliplatin + S-1; TNF, tumour necrosis factor.
