# Supplementary material for: Single‐cell characterization of differentiation trajectories and drug resistance features in gastric cancer with peritoneal metastasis
Source: Clin Transl Med. 2024 Oct 18;14(10):e70054. doi: 10.1002/ctm2.70054 (PMC11488346; doi:10.1002/ctm2.70054)
Supplement: Supplementary file 5 — Supporting Information [file CTM2-14-e70054-s003.docx]

**Supplementary table 5.** Baseline characteristics of the included cases in cohort 2 and cohort 3.

| **Patient ID** | **Age** | **Gender** | **Lauren type** | **Tissue type** |
| --- | --- | --- | --- | --- |
| 1 | 81 | Female | Mixed | PT and PM |
| 2 | 48 | Female | Diffuse | PT and PM |
| 3 | 44 | Female | Intestinal | PT and PM |
| 4 | 37 | Female | Diffuse | PT and ascites |
| 5 | 74 | Male | NA | PT and ascites |
| 6 | 55 | Female | Diffuse | PT and ascites |
| 7 | 52 | Male | Intestinal | PT |
| 8 | 35 | Male | Diffuse | PT |
| 9 | 72 | Female | Mixed | PT |

PT, primary tumor; PM, peritoneal metastasis.
