# Supplementary material for: Single‐cell characterization of differentiation trajectories and drug resistance features in gastric cancer with peritoneal metastasis
Source: Clin Transl Med. 2024 Oct 18;14(10):e70054. doi: 10.1002/ctm2.70054 (PMC11488346; doi:10.1002/ctm2.70054)
Supplement: Supplementary file 6 — Supporting Information [file CTM2-14-e70054-s006.docx]

**Supplementary table 6.** Baseline characteristics of the included cases in cohort 4.

| **ID** | **Age** | **Sex** | **Stage** | **Lauren type** | **HER2** | **GCPM** |
| --- | --- | --- | --- | --- | --- | --- |
| 1 | 45 | Male | IV | Intestinal | Positive | No |
| 2 | 36 | Male | III | Diffuse | Positive | No |
| 3 | 69 | Male | IV | Intestinal | Positive | No |
| 4 | 62 | Male | IV | Intestinal | Positive | No |
| 5 | 51 | Male | III | Mixed | Negative | No |
| 6 | 91 | Male | IIIA | Intestinal | Positive | No |
| 7 | 72 | Male | III | Intestinal | Negative | No |
| 8 | 66 | Male | IIIB | Intestinal | Positive | No |
| 9 | 58 | Male | III | Intestinal | Positive | No |
| 10 | 66 | Male | IIIc | Intestinal | Positive | No |
| 11 | 67 | Male | IV | Intestinal | Positive | No |
| 12 | 48 | Male | IV | Intestinal | Positive | No |
| 13 | 83 | Female | IV | Intestinal | Positive | No |
| 14 | 73 | Female | IV | Intestinal | Positive | No |
| 15 | 59 | Female | IV | Diffuse | Positive | No |
| 16 | 60 | Female | IV | Mixed | Positive | No |
| 17 | 58 | Male | IV | Intestinal | Positive | Yes |
| 18 | 52 | Male | IV | Diffuse | Positive | Yes |
| 19 | 63 | Male | IV | Mixed | Positive | Yes |
| 20 | 81 | Male | IV | Intestinal | Positive | Yes |
| 21 | 53 | Male | IV | Intestinal | Positive | Yes |
| 22 | 63 | Male | IV | Mixed | Positive | Yes |
| 23 | 54 | Male | IV | Mixed | Positive | Yes |
| 24 | 63 | Male | IV | Intestinal | Positive | Yes |
| 25 | 66 | Male | IV | Intestinal | Positive | Yes |
| 26 | 60 | Male | IV | Intestinal | Positive | Yes |
| 27 | 78 | Male | IV | Intestinal | Positive | Yes |
| 28 | 63 | Male | IV | Intestinal | Positive | Yes |
| 29 | 28 | Male | IV | Intestinal | Positive | Yes |
| 30 | 56 | Female | IV | Diffuse | Positive | Yes |

GCPM, gastric cancer peritoneal metastasis.
