# Supplementary material for: Single‐cell characterization of differentiation trajectories and drug resistance features in gastric cancer with peritoneal metastasis
Source: Clin Transl Med. 2024 Oct 18;14(10):e70054. doi: 10.1002/ctm2.70054 (PMC11488346; doi:10.1002/ctm2.70054)
Supplement: Supplementary file 7 — Supporting Information [file CTM2-14-e70054-s009.docx]

**Supplementary table 7.** Clinicopathologic characteristics of the included patients in the SHR-1701 cohort.

| **ID** | **Gender** | **Age** | **Lauren type** | **GCPM** | **PFS** | **PFS status** | **Response** |
| --- | --- | --- | --- | --- | --- | --- | --- |
| 1 | Male | 76 | Intestinal | No | 387 | Progression | PD |
| 2 | Male | 45 | Mixed | No | 229 | Progression | PD |
| 3 | Male | 75 | Intestinal | No | 46 | Progression | PD |
| 4 | Male | 69 | Intestinal | No | 39 | Progression | PD |
| 5 | Male | 63 | Mixed | No | 239 | Progression | SD |
| 6 | Female | 52 | Intestinal | No | 36 | Progression | PD |
| 7 | Female | 53 | Mixed | No | 33 | Progression | PD |
| 8 | Male | 70 | Intestinal | No | 43 | Progression | PD |
| 9 | Male | 61 | NA | No | 125 | Progression-free | PD |
| 10 | Male | 59 | Mixed | No | 21 | Progression-free | SD |
| 11 | Female | 77 | NA | No | 155 | Progression-free | PR |
| 12 | Female | 44 | Mixed | Yes | 84 | Progression | PD |
| 13 | Male | 70 | Intestinal | Yes | 173 | Progression | SD |
| 14 | Male | 58 | NA | Yes | 559 | Progression | PR |
| 15 | Male | 58 | Mixed | Yes | 42 | Progression | PD |
| 16 | Male | 65 | Intestinal | Yes | 336 | Progression-free | SD |
| 17 | Female | 47 | Mixed | Yes | 164 | Progression-free | PR |
| 18 | Female | 58 | Intestinal | Yes | 297 | Progression-free | PR |
| 19 | Male | 65 | Intestinal | Yes | 84 | Progression | SD |
| 20 | Male | 70 | Intestinal | Yes | 264 | Progression-free | PR |

GCPM, gastric cancer patients with peritoneal metastasis; PFS, progression-free survival; PD, progressive disease; PR, partial response; SD, stable disease. NA, non-available.
