# Supplementary material for: Single‐cell characterization of differentiation trajectories and drug resistance features in gastric cancer with peritoneal metastasis
Source: Clin Transl Med. 2024 Oct 18;14(10):e70054. doi: 10.1002/ctm2.70054 (PMC11488346; doi:10.1002/ctm2.70054)
Supplement: Supplementary file 8 — Supporting Information [file CTM2-14-e70054-s010.docx]

**Supplementary table 8.** Baseline characteristics of the included cases in the PKUCHIO cohort.

| **Patient ID** | **Gender** | **Age** | **PFS** | **Peritoneal Metastasis** | **cTNM stage** | **Differentiation** | **Lauren type** | **Response** |
| --- | --- | --- | --- | --- | --- | --- | --- | --- |
| 1 | Female | ≥65 | 34 | No | IV | Low | Intestinal | CR/PR |
| 2 | Male | <65 | 40 | No | IV | Low | Mixed | CR/PR |
| 3 | Male | <65 | 41 | No | IV | NA | Diffuse | CR/PR |
| 4 | Male | ≥65 | 42 | No | IV | Moderate | Intestinal | CR/PR |
| 5 | Male | <65 | 42 | Yes | IV | Moderate | Intestinal | CR/PR |
| 6 | Male | <65 | 42 | Yes | IV | Low | NA | CR/PR |
| 7 | Male | ≥65 | 46 | No | IV | Moderate | Intestinal | CR/PR |
| 8 | Male | ≥65 | 52 | No | IV | Low | Intestinal | CR/PR |
| 9 | Male | <65 | 58 | No | IV | Low | Mixed | CR/PR |
| 10 | Male | ≥65 | 59 | No | IV | Low | Intestinal | CR/PR |
| 11 | Female | <65 | 62 | Yes | IV | Moderate | Intestinal | CR/PR |
| 12 | Male | ≥65 | 62 | No | IV | Moderate | Intestinal | CR/PR |
| 13 | Male | ≥65 | 64 | No | IV | Low | Intestinal | CR/PR |
| 14 | Male | ≥65 | 68 | No | IV | Low | Intestinal | CR/PR |
| 15 | Male | ≥65 | 70 | No | NA | Moderate | Intestinal | CR/PR |
| 16 | Male | ≥65 | 84 | No | IV | Low | Diffuse | CR/PR |
| 17 | Male | <65 | 84 | No | IV | Low | Intestinal | CR/PR |
| 18 | Male | <65 | 85 | Yes | IV | Low | Diffuse | CR/PR |
| 19 | Male | ≥65 | 86 | No | IV | Low | Intestinal | CR/PR |
| 20 | Male | <65 | 86 | No | IV | Low | Intestinal | CR/PR |
| 21 | Male | <65 | 89 | No | IV | Low | Diffuse | CR/PR |
| 22 | Male | <65 | 92 | No | IV | Low | Mixed | CR/PR |
| 23 | Female | <65 | 96 | No | IV | Low | Diffuse | CR/PR |
| 24 | Male | <65 | 99 | No | IV | Low | Intestinal | CR/PR |
| 25 | Female | <65 | 99 | No | III | Low | Diffuse | CR/PR |
| 26 | Female | ≥65 | 100 | Yes | IV | Low | Intestinal | CR/PR |
| 27 | Male | <65 | 101 | No | IV | Moderate | Intestinal | CR/PR |
| 28 | Male | ≥65 | 101 | Yes | IV | Low | Diffuse | CR/PR |
| 29 | Female | <65 | 102 | No | IV | Moderate | Intestinal | CR/PR |
| 30 | Male | <65 | 103 | No | IV | Low | Intestinal | CR/PR |
| 31 | Male | <65 | 104 | Yes | IV | Low | Intestinal | CR/PR |
| 32 | Male | ≥65 | 104 | Yes | IV | Low | Intestinal | CR/PR |
| 33 | Male | ≥65 | 109 | No | IV | Low | Diffuse | CR/PR |
| 34 | Female | <65 | 109 | No | IV | Low | Diffuse | CR/PR |
| 35 | Female | <65 | 112 | Yes | IV | Low | Diffuse | CR/PR |
| 36 | Male | <65 | 112 | No | IV | Low | Intestinal | CR/PR |
| 37 | Male | ≥65 | 113 | No | IV | Low | Intestinal | CR/PR |
| 38 | Male | ≥65 | 120 | No | IV | Moderate | Intestinal | CR/PR |
| 39 | Female | <65 | 123 | Yes | IV | Low | Mixed | CR/PR |
| 40 | Male | <65 | 125 | No | IV | Low | Mixed | CR/PR |
| 41 | Male | <65 | 125 | No | IV | Low | Intestinal | CR/PR |
| 42 | Female | <65 | 126 | No | IV | NA | Intestinal | CR/PR |
| 43 | Female | <65 | 127 | No | IV | Low | Mixed | CR/PR |
| 44 | Female | <65 | 127 | No | IV | Low | Diffuse | CR/PR |
| 45 | Male | <65 | 128 | No | IV | Moderate | Intestinal | CR/PR |
| 46 | Male | ≥65 | 131 | Yes | IV | Low | Intestinal | CR/PR |
| 47 | Male | ≥65 | 135 | Yes | IV | Moderate | Intestinal | CR/PR |
| 48 | Male | ≥65 | 137 | No | III | Moderate | Intestinal | CR/PR |
| 49 | Male | <65 | 139 | Yes | IV | Low | Mixed | CR/PR |
| 50 | Female | <65 | 141 | Yes | IV | Low | Diffuse | CR/PR |
| 51 | Female | <65 | 143 | No | IV | Low | Diffuse | CR/PR |
| 52 | Male | <65 | 147 | No | IV | Moderate | NA | CR/PR |
| 53 | Male | ≥65 | 148 | No | IV | Moderate | Intestinal | CR/PR |
| 54 | Female | <65 | 149 | Yes | IV | Low | Mixed | CR/PR |
| 55 | Male | <65 | 151 | No | IV | Moderate | NA | CR/PR |
| 56 | Male | <65 | 152 | Yes | IV | Low | Diffuse | CR/PR |
| 57 | Female | ≥65 | 153 | No | NA | Low | Diffuse | CR/PR |
| 58 | Male | ≥65 | 153 | No | IV | Low | Diffuse | CR/PR |
| 59 | Male | ≥65 | 153 | No | IV | Low | Mixed | CR/PR |
| 60 | Female | <65 | 154 | No | IV | Low | Diffuse | CR/PR |
| 61 | Male | ≥65 | 154 | No | IV | Low | Intestinal | CR/PR |
| 62 | Male | ≥65 | 158 | No | IV | Low | Mixed | CR/PR |
| 63 | Male | <65 | 163 | Yes | IV | Low | Mixed | CR/PR |
| 64 | Female | <65 | 166 | No | IV | Low | Mixed | CR/PR |
| 65 | Female | <65 | 167 | Yes | IV | Low | Intestinal | CR/PR |
| 66 | Male | <65 | 167 | No | IV | Low | Intestinal | CR/PR |
| 67 | Male | ≥65 | 168 | No | IV | NA | NA | CR/PR |
| 68 | Male | ≥65 | 169 | No | IV | Moderate | Intestinal | CR/PR |
| 69 | Male | <65 | 169 | No | IV | Moderate | Intestinal | CR/PR |
| 70 | Male | ≥65 | 171 | No | IV | Low | Mixed | CR/PR |
| 71 | Male | ≥65 | 172 | No | IV | Low | Diffuse | CR/PR |
| 72 | Male | <65 | 173 | No | IV | Low | Diffuse | CR/PR |
| 73 | Male | <65 | 176 | No | IV | NA | Diffuse | CR/PR |
| 74 | Male | <65 | 190 | No | IV | NA | Intestinal | CR/PR |
| 75 | Male | ≥65 | 192 | Yes | IV | Low | Intestinal | CR/PR |
| 76 | Male | <65 | 194 | Yes | IV | Low | Intestinal | CR/PR |
| 77 | Male | ≥65 | 194 | No | IV | Moderate | Intestinal | CR/PR |
| 78 | Male | ≥65 | 195 | No | IV | Low | Intestinal | CR/PR |
| 79 | Male | <65 | 204 | No | IV | Low | Intestinal | CR/PR |
| 80 | Female | <65 | 209 | No | IV | Low | Diffuse | CR/PR |
| 81 | Male | ≥65 | 210 | No | IV | Moderate | Intestinal | CR/PR |
| 82 | Female | ≥65 | 210 | No | IV | Moderate | Intestinal | CR/PR |
| 83 | Male | ≥65 | 213 | No | IV | Moderate | Intestinal | CR/PR |
| 84 | Female | ≥65 | 214 | Yes | IV | Low | Diffuse | CR/PR |
| 85 | Male | ≥65 | 216 | Yes | IV | Moderate | Mixed | CR/PR |
| 86 | Female | <65 | 217 | No | IV | Low | Intestinal | CR/PR |
| 87 | Male | <65 | 218 | No | IV | Moderate | Intestinal | CR/PR |
| 88 | Male | ≥65 | 219 | No | IV | Low | Intestinal | CR/PR |
| 89 | Male | ≥65 | 223 | No | IV | Low | Mixed | CR/PR |
| 90 | Female | <65 | 225 | No | IV | Low | Diffuse | CR/PR |
| 91 | Male | <65 | 225 | No | IV | Low | Intestinal | CR/PR |
| 92 | Male | ≥65 | 226 | No | IV | Moderate | Intestinal | CR/PR |
| 93 | Male | <65 | 231 | Yes | IV | Low | Diffuse | CR/PR |
| 94 | Male | ≥65 | 233 | No | IV | Low | Mixed | CR/PR |
| 95 | Male | ≥65 | 238 | No | IV | Low | Intestinal | CR/PR |
| 96 | Male | ≥65 | 240 | No | IV | Low | Intestinal | CR/PR |
| 97 | Female | <65 | 253 | No | IV | Moderate | Intestinal | CR/PR |
| 98 | Male | ≥65 | 258 | No | IV | Low | Mixed | CR/PR |
| 99 | Male | ≥65 | 259 | Yes | IV | Low | Mixed | CR/PR |
| 100 | Female | ≥65 | 260 | Yes | IV | Low | Mixed | CR/PR |
| 101 | Male | ≥65 | 263 | Yes | IV | Low | Mixed | CR/PR |
| 102 | Male | ≥65 | 266 | No | III | Low | Diffuse | CR/PR |
| 103 | Female | <65 | 269 | No | IV | Low | NA | CR/PR |
| 104 | Male | <65 | 272 | No | IV | Moderate | Intestinal | CR/PR |
| 105 | Male | <65 | 278 | No | IV | Moderate | Intestinal | CR/PR |
| 106 | Female | <65 | 279 | No | IV | Low | Mixed | CR/PR |
| 107 | Male | ≥65 | 280 | No | IV | Low | Intestinal | CR/PR |
| 108 | Male | <65 | 288 | Yes | IV | Moderate | Intestinal | CR/PR |
| 109 | Male | <65 | 294 | No | IV | Moderate | Intestinal | CR/PR |
| 110 | Female | ≥65 | 296 | No | IV | Low | Intestinal | CR/PR |
| 111 | Male | <65 | 296 | No | IV | Moderate | Intestinal | CR/PR |
| 112 | Female | <65 | 297 | No | IV | Low | Diffuse | CR/PR |
| 113 | Male | ≥65 | 297 | No | IV | Low | Mixed | CR/PR |
| 114 | Female | ≥65 | 307 | No | IV | Moderate | Intestinal | CR/PR |
| 115 | Male | ≥65 | 332 | Yes | IV | Moderate | Intestinal | CR/PR |
| 116 | Female | ≥65 | 335 | Yes | IV | Low | Intestinal | CR/PR |
| 117 | Male | <65 | 336 | No | IV | Moderate | Intestinal | CR/PR |
| 118 | Male | <65 | 337 | No | IV | Low | Mixed | CR/PR |
| 119 | Male | ≥65 | 340 | No | IV | Low | Diffuse | CR/PR |
| 120 | Male | ≥65 | 342 | No | IV | Low | Diffuse | CR/PR |
| 121 | Male | ≥65 | 343 | No | IV | Low | Mixed | CR/PR |
| 122 | Female | ≥65 | 343 | Yes | IV | Low | Mixed | CR/PR |
| 123 | Female | ≥65 | 346 | No | IV | Low | Intestinal | CR/PR |
| 124 | Male | ≥65 | 353 | No | IV | Low | Diffuse | CR/PR |
| 125 | Male | ≥65 | 358 | No | IV | Low | Intestinal | CR/PR |
| 126 | Male | <65 | 366 | No | IV | Moderate | Intestinal | CR/PR |
| 127 | Female | ≥65 | 368 | No | IV | Low | Diffuse | CR/PR |
| 128 | Male | <65 | 379 | No | IV | Low | Mixed | CR/PR |
| 129 | Male | <65 | 385 | No | IV | Moderate | Intestinal | CR/PR |
| 130 | Male | ≥65 | 410 | No | IV | Moderate | Intestinal | CR/PR |
| 131 | Male | ≥65 | 413 | No | IV | Moderate | NA | CR/PR |
| 132 | Male | <65 | 417 | No | IV | Low | Diffuse | CR/PR |
| 133 | Male | <65 | 420 | No | IV | Moderate | Intestinal | CR/PR |
| 134 | Female | ≥65 | 422 | Yes | IV | Low | Mixed | CR/PR |
| 135 | Female | ≥65 | 436 | No | IV | Moderate | Intestinal | CR/PR |
| 136 | Male | <65 | 443 | No | IV | Low | Mixed | CR/PR |
| 137 | Male | ≥65 | 450 | No | IV | Low | Mixed | CR/PR |
| 138 | Male | ≥65 | 450 | No | IV | Low | Mixed | CR/PR |
| 139 | Male | ≥65 | 451 | No | IV | Moderate | Intestinal | CR/PR |
| 140 | Male | ≥65 | 452 | No | IV | Low | Mixed | CR/PR |
| 141 | Male | ≥65 | 460 | No | IV | Moderate | Intestinal | CR/PR |
| 142 | Male | <65 | 463 | No | IV | Low | Diffuse | CR/PR |
| 143 | Female | ≥65 | 468 | No | IV | Low | Mixed | CR/PR |
| 144 | Male | <65 | 470 | No | IV | Moderate | Diffuse | CR/PR |
| 145 | Male | ≥65 | 475 | No | IV | Low | Intestinal | CR/PR |
| 146 | Male | ≥65 | 483 | No | IV | Low | Mixed | CR/PR |
| 147 | Female | <65 | 496 | Yes | II | Moderate | Intestinal | CR/PR |
| 148 | Female | ≥65 | 497 | No | IV | Low | Diffuse | CR/PR |
| 149 | Male | <65 | 498 | No | IV | Moderate | Intestinal | CR/PR |
| 150 | Female | <65 | 513 | No | IV | Low | Mixed | CR/PR |
| 151 | Male | ≥65 | 515 | No | III | Moderate | Intestinal | CR/PR |
| 152 | Male | ≥65 | 516 | No | IV | Low | Intestinal | CR/PR |
| 153 | Male | ≥65 | 521 | Yes | IV | Low | Intestinal | CR/PR |
| 154 | Male | <65 | 526 | No | NA | Moderate | Intestinal | CR/PR |
| 155 | Female | ≥65 | 547 | No | IV | Low | Diffuse | CR/PR |
| 156 | Male | ≥65 | 555 | No | IV | Low | NA | CR/PR |
| 157 | Male | <65 | 564 | No | IV | Low | Diffuse | CR/PR |
| 158 | Male | <65 | 594 | No | III | Low | Mixed | CR/PR |
| 159 | Male | <65 | 595 | No | IV | Moderate | Intestinal | CR/PR |
| 160 | Male | <65 | 596 | No | IV | Low | Intestinal | CR/PR |
| 161 | Male | <65 | 602 | Yes | IV | Low | Mixed | CR/PR |
| 162 | Female | ≥65 | 608 | No | IV | Low | Mixed | CR/PR |
| 163 | Male | <65 | 678 | No | IV | Low | Diffuse | CR/PR |
| 164 | Male | ≥65 | 697 | No | IV | Low | Mixed | CR/PR |
| 165 | Male | <65 | 702 | No | IV | Low | Intestinal | CR/PR |
| 166 | Male | ≥65 | 730 | No | IV | Low | NA | CR/PR |
| 167 | Male | ≥65 | 734 | No | IV | Low | Mixed | CR/PR |
| 168 | Male | ≥65 | 765 | No | IV | Low | Mixed | CR/PR |
| 169 | Male | <65 | 790 | No | IV | Moderate | Intestinal | CR/PR |
| 170 | Female | <65 | 819 | No | IV | Low | Diffuse | CR/PR |
| 171 | Male | ≥65 | 860 | No | IV | Moderate | Intestinal | CR/PR |
| 172 | Female | <65 | 867 | No | IV | Moderate | Intestinal | CR/PR |
| 173 | Male | <65 | 878 | No | IV | Low | Mixed | CR/PR |
| 174 | Male | <65 | 895 | No | IV | Low | Intestinal | CR/PR |
| 175 | Male | <65 | 913 | No | IV | Low | Mixed | CR/PR |
| 176 | Male | ≥65 | 943 | No | IV | Low | Diffuse | CR/PR |
| 177 | Female | ≥65 | 1502 | No | IV | Low | NA | CR/PR |
| 178 | Male | ≥65 | 8 | Yes | IV | Low | Diffuse | SD/PD |
| 179 | Male | ≥65 | 10 | No | IV | Low | Mixed | SD/PD |
| 180 | Male | ≥65 | 13 | Yes | IV | Moderate | Intestinal | SD/PD |
| 181 | Female | <65 | 14 | No | IV | Low | Diffuse | SD/PD |
| 182 | Male | <65 | 14 | No | IV | Low | Diffuse | SD/PD |
| 183 | Female | <65 | 15 | No | IV | Low | Diffuse | SD/PD |
| 184 | Female | <65 | 18 | Yes | IV | Low | Intestinal | SD/PD |
| 185 | Male | ≥65 | 19 | No | IV | NA | Intestinal | SD/PD |
| 186 | Male | <65 | 20 | No | IV | Moderate | Intestinal | SD/PD |
| 187 | Female | <65 | 22 | No | IV | Moderate | Intestinal | SD/PD |
| 188 | Female | <65 | 23 | Yes | IV | Low | Diffuse | SD/PD |
| 189 | Male | ≥65 | 23 | No | IV | Low | Diffuse | SD/PD |
| 190 | Female | <65 | 25 | Yes | IV | Low | Mixed | SD/PD |
| 191 | Male | <65 | 25 | No | IV | Low | Diffuse | SD/PD |
| 192 | Male | ≥65 | 26 | Yes | IV | Low | Diffuse | SD/PD |
| 193 | Male | <65 | 26 | Yes | IV | Moderate | Intestinal | SD/PD |
| 194 | Female | <65 | 26 | No | IV | Low | Mixed | SD/PD |
| 195 | Female | <65 | 27 | Yes | IV | Low | Diffuse | SD/PD |
| 196 | Male | <65 | 29 | No | IV | Moderate | Intestinal | SD/PD |
| 197 | Female | <65 | 29 | Yes | IV | Moderate | Diffuse | SD/PD |
| 198 | Male | ≥65 | 29 | Yes | IV | Low | Intestinal | SD/PD |
| 199 | Female | <65 | 30 | No | IV | Low | Mixed | SD/PD |
| 200 | Male | ≥65 | 30 | Yes | IV | Low | Diffuse | SD/PD |
| 201 | Male | <65 | 31 | No | IV | Low | Mixed | SD/PD |
| 202 | Male | <65 | 32 | No | IV | Low | Intestinal | SD/PD |
| 203 | Male | <65 | 32 | No | IV | Low | Mixed | SD/PD |
| 204 | Male | <65 | 32 | Yes | IV | Low | Mixed | SD/PD |
| 205 | Male | ≥65 | 33 | No | III | Low | Mixed | SD/PD |
| 206 | Male | <65 | 33 | No | IV | Low | Mixed | SD/PD |
| 207 | Male | ≥65 | 33 | No | IV | Low | Intestinal | SD/PD |
| 208 | Male | ≥65 | 34 | No | IV | Low | Mixed | SD/PD |
| 209 | Male | <65 | 34 | Yes | IV | Moderate | Intestinal | SD/PD |
| 210 | Female | <65 | 35 | No | IV | Low | Intestinal | SD/PD |
| 211 | Male | <65 | 35 | No | IV | Moderate | Intestinal | SD/PD |
| 212 | Male | <65 | 36 | No | IV | Low | Mixed | SD/PD |
| 213 | Female | <65 | 36 | No | IV | Low | Diffuse | SD/PD |
| 214 | Male | <65 | 37 | Yes | IV | Low | Diffuse | SD/PD |
| 215 | Male | ≥65 | 37 | Yes | IV | Low | Diffuse | SD/PD |
| 216 | Female | ≥65 | 37 | Yes | IV | Low | Diffuse | SD/PD |
| 217 | Female | <65 | 37 | No | IV | Low | Mixed | SD/PD |
| 218 | Male | <65 | 37 | Yes | IV | Low | Diffuse | SD/PD |
| 219 | Male | <65 | 38 | No | IV | Low | Diffuse | SD/PD |
| 220 | Male | ≥65 | 38 | No | IV | Moderate | Intestinal | SD/PD |
| 221 | Male | ≥65 | 38 | No | IV | Moderate | Intestinal | SD/PD |
| 222 | Male | <65 | 38 | Yes | IV | Low | Mixed | SD/PD |
| 223 | Male | <65 | 38 | Yes | IV | Moderate | Intestinal | SD/PD |
| 224 | Female | ≥65 | 39 | No | IV | Low | Mixed | SD/PD |
| 225 | Male | ≥65 | 39 | No | IV | Low | Mixed | SD/PD |
| 226 | Female | ≥65 | 39 | Yes | IV | Low | Diffuse | SD/PD |
| 227 | Male | ≥65 | 40 | No | IV | Low | NA | SD/PD |
| 228 | Male | <65 | 40 | No | IV | Low | Mixed | SD/PD |
| 229 | Female | <65 | 41 | No | IV | Low | Mixed | SD/PD |
| 230 | Male | <65 | 41 | Yes | IV | Low | Mixed | SD/PD |
| 231 | Female | <65 | 42 | No | III | Low | Diffuse | SD/PD |
| 232 | Female | <65 | 42 | Yes | IV | Low | Diffuse | SD/PD |
| 233 | Female | <65 | 42 | No | IV | Low | Diffuse | SD/PD |
| 234 | Male | <65 | 42 | Yes | IV | Moderate | Intestinal | SD/PD |
| 235 | Male | <65 | 42 | No | IV | Moderate | Intestinal | SD/PD |
| 236 | Male | ≥65 | 42 | Yes | IV | Low | Mixed | SD/PD |
| 237 | Male | ≥65 | 42 | No | IV | Low | Intestinal | SD/PD |
| 238 | Male | ≥65 | 42 | No | IV | Low | Intestinal | SD/PD |
| 239 | Male | <65 | 43 | No | IV | Moderate | Mixed | SD/PD |
| 240 | Male | ≥65 | 43 | No | IV | NA | Diffuse | SD/PD |
| 241 | Male | ≥65 | 43 | No | IV | Low | Intestinal | SD/PD |
| 242 | Male | <65 | 44 | No | IV | Low | Diffuse | SD/PD |
| 243 | Female | <65 | 44 | Yes | IV | Low | Diffuse | SD/PD |
| 244 | Male | <65 | 44 | No | II | Low | Diffuse | SD/PD |
| 245 | Male | <65 | 44 | No | IV | NA | NA | SD/PD |
| 246 | Male | <65 | 44 | Yes | IV | Low | Mixed | SD/PD |
| 247 | Male | <65 | 45 | Yes | IV | Low | Diffuse | SD/PD |
| 248 | Male | <65 | 45 | Yes | IV | Low | Mixed | SD/PD |
| 249 | Male | ≥65 | 46 | No | IV | Low | Diffuse | SD/PD |
| 250 | Female | <65 | 46 | Yes | IV | Low | Diffuse | SD/PD |
| 251 | Male | ≥65 | 46 | No | IV | Low | Intestinal | SD/PD |
| 252 | Male | <65 | 46 | No | NA | Moderate | Intestinal | SD/PD |
| 253 | Male | ≥65 | 47 | No | IV | Moderate | Intestinal | SD/PD |
| 254 | Female | ≥65 | 47 | No | IV | Low | NA | SD/PD |
| 255 | Female | <65 | 48 | Yes | IV | NA | Diffuse | SD/PD |
| 256 | Male | <65 | 48 | No | IV | Low | Diffuse | SD/PD |
| 257 | Male | ≥65 | 49 | No | IV | NA | Diffuse | SD/PD |
| 258 | Male | ≥65 | 49 | No | IV | Low | Intestinal | SD/PD |
| 259 | Female | <65 | 49 | No | IV | Low | Diffuse | SD/PD |
| 260 | Male | <65 | 50 | No | IV | Low | Intestinal | SD/PD |
| 261 | Female | <65 | 50 | Yes | IV | Low | Diffuse | SD/PD |
| 262 | Female | <65 | 50 | Yes | IV | Low | Diffuse | SD/PD |
| 263 | Female | <65 | 50 | Yes | IV | Low | Mixed | SD/PD |
| 264 | Female | <65 | 51 | Yes | IV | Low | Diffuse | SD/PD |
| 265 | Male | <65 | 51 | No | IV | Low | Intestinal | SD/PD |
| 266 | Male | <65 | 52 | No | IV | Low | Diffuse | SD/PD |
| 267 | Male | <65 | 52 | No | IV | Moderate | NA | SD/PD |
| 268 | Female | <65 | 52 | No | IV | Low | NA | SD/PD |
| 269 | Male | <65 | 53 | No | IV | Moderate | Intestinal | SD/PD |
| 270 | Female | <65 | 53 | Yes | IV | Low | Diffuse | SD/PD |
| 271 | Female | <65 | 53 | No | IV | Low | Mixed | SD/PD |
| 272 | Male | ≥65 | 54 | Yes | IV | Moderate | Intestinal | SD/PD |
| 273 | Female | <65 | 55 | Yes | IV | Low | Diffuse | SD/PD |
| 274 | Male | <65 | 55 | No | IV | Low | Intestinal | SD/PD |
| 275 | Male | <65 | 56 | No | IV | Low | Diffuse | SD/PD |
| 276 | Female | <65 | 56 | No | III | Low | Diffuse | SD/PD |
| 277 | Male | ≥65 | 56 | No | IV | Moderate | Intestinal | SD/PD |
| 278 | Female | <65 | 56 | Yes | IV | Low | Diffuse | SD/PD |
| 279 | Female | <65 | 57 | No | IV | Moderate | Intestinal | SD/PD |
| 280 | Male | ≥65 | 57 | Yes | IV | Moderate | Intestinal | SD/PD |
| 281 | Female | <65 | 58 | No | IV | Moderate | Mixed | SD/PD |
| 282 | Female | <65 | 58 | No | IV | Low | Diffuse | SD/PD |
| 283 | Male | ≥65 | 59 | No | IV | Low | Intestinal | SD/PD |
| 284 | Male | ≥65 | 60 | No | IV | Low | Intestinal | SD/PD |
| 285 | Male | ≥65 | 60 | No | IV | Moderate | Intestinal | SD/PD |
| 286 | Female | <65 | 60 | Yes | IV | Low | Diffuse | SD/PD |
| 287 | Male | <65 | 61 | No | IV | Moderate | Intestinal | SD/PD |
| 288 | Male | ≥65 | 62 | No | IV | Low | Intestinal | SD/PD |
| 289 | Female | ≥65 | 62 | No | IV | Low | Mixed | SD/PD |
| 290 | Female | <65 | 63 | No | IV | Low | Mixed | SD/PD |
| 291 | Male | <65 | 63 | No | IV | Moderate | Intestinal | SD/PD |
| 292 | Male | <65 | 64 | No | IV | Moderate | Intestinal | SD/PD |
| 293 | Male | <65 | 64 | No | IV | Low | Diffuse | SD/PD |
| 294 | Male | <65 | 65 | No | III | NA | Diffuse | SD/PD |
| 295 | Male | <65 | 66 | Yes | IV | Low | Diffuse | SD/PD |
| 296 | Female | <65 | 67 | Yes | IV | Low | Mixed | SD/PD |
| 297 | Female | <65 | 67 | No | IV | Moderate | Intestinal | SD/PD |
| 298 | Male | <65 | 69 | Yes | IV | Low | Mixed | SD/PD |
| 299 | Male | <65 | 70 | Yes | IV | Moderate | Intestinal | SD/PD |
| 300 | Male | ≥65 | 72 | No | III | Low | Intestinal | SD/PD |
| 301 | Female | <65 | 73 | Yes | IV | Low | Mixed | SD/PD |
| 302 | Female | <65 | 75 | Yes | IV | Low | Mixed | SD/PD |
| 303 | Male | ≥65 | 75 | No | IV | Moderate | Intestinal | SD/PD |
| 304 | Male | ≥65 | 76 | Yes | III | Low | Intestinal | SD/PD |
| 305 | Male | <65 | 76 | No | IV | Low | Intestinal | SD/PD |
| 306 | Female | ≥65 | 76 | No | IV | Low | Mixed | SD/PD |
| 307 | Male | <65 | 77 | Yes | IV | NA | Diffuse | SD/PD |
| 308 | Male | ≥65 | 77 | No | IV | Moderate | Intestinal | SD/PD |
| 309 | Male | <65 | 78 | No | IV | Moderate | Intestinal | SD/PD |
| 310 | Female | <65 | 78 | No | IV | Low | Diffuse | SD/PD |
| 311 | Female | <65 | 79 | No | IV | Low | Mixed | SD/PD |
| 312 | Female | ≥65 | 79 | No | IV | Low | Diffuse | SD/PD |
| 313 | Female | <65 | 80 | Yes | IV | Low | NA | SD/PD |
| 314 | Male | ≥65 | 82 | No | IV | Low | Intestinal | SD/PD |
| 315 | Male | <65 | 83 | No | IV | Low | Mixed | SD/PD |
| 316 | Male | <65 | 83 | No | IV | NA | Diffuse | SD/PD |
| 317 | Male | ≥65 | 83 | No | NA | Moderate | Intestinal | SD/PD |
| 318 | Male | <65 | 83 | No | IV | Low | Intestinal | SD/PD |
| 319 | Male | <65 | 84 | Yes | IV | NA | Diffuse | SD/PD |
| 320 | Female | ≥65 | 84 | No | IV | Moderate | Intestinal | SD/PD |
| 321 | Male | ≥65 | 84 | No | IV | Low | Diffuse | SD/PD |
| 322 | Male | <65 | 85 | Yes | IV | Low | Diffuse | SD/PD |
| 323 | Female | <65 | 85 | No | IV | Low | Mixed | SD/PD |
| 324 | Female | <65 | 85 | No | IV | Low | Diffuse | SD/PD |
| 325 | Female | <65 | 85 | No | IV | Low | Diffuse | SD/PD |
| 326 | Female | <65 | 86 | Yes | IV | Low | Diffuse | SD/PD |
| 327 | Male | ≥65 | 86 | Yes | IV | NA | Diffuse | SD/PD |
| 328 | Male | ≥65 | 86 | Yes | IV | Low | Mixed | SD/PD |
| 329 | Male | ≥65 | 86 | Yes | IV | Low | Mixed | SD/PD |
| 330 | Male | <65 | 87 | Yes | IV | Moderate | Intestinal | SD/PD |
| 331 | Male | ≥65 | 87 | Yes | IV | Low | Diffuse | SD/PD |
| 332 | Male | ≥65 | 88 | No | IV | Low | Intestinal | SD/PD |
| 333 | Male | ≥65 | 89 | Yes | IV | Low | Diffuse | SD/PD |
| 334 | Male | <65 | 89 | Yes | IV | Moderate | Intestinal | SD/PD |
| 335 | Female | <65 | 89 | Yes | IV | Low | Diffuse | SD/PD |
| 336 | Male | <65 | 90 | No | IV | Low | Mixed | SD/PD |
| 337 | Male | ≥65 | 90 | No | IV | Low | NA | SD/PD |
| 338 | Female | <65 | 90 | No | IV | Low | Diffuse | SD/PD |
| 339 | Female | <65 | 90 | Yes | IV | Moderate | Intestinal | SD/PD |
| 340 | Male | ≥65 | 90 | Yes | III | Low | Mixed | SD/PD |
| 341 | Female | <65 | 90 | No | IV | Low | Diffuse | SD/PD |
| 342 | Male | ≥65 | 90 | No | IV | Moderate | Intestinal | SD/PD |
| 343 | Male | ≥65 | 92 | Yes | III | Low | Mixed | SD/PD |
| 344 | Male | <65 | 92 | Yes | IV | Moderate | Intestinal | SD/PD |
| 345 | Male | ≥65 | 92 | No | IV | Low | Diffuse | SD/PD |
| 346 | Male | <65 | 94 | No | IV | Low | NA | SD/PD |
| 347 | Male | <65 | 94 | Yes | IV | Low | Diffuse | SD/PD |
| 348 | Female | <65 | 95 | Yes | IV | Low | Mixed | SD/PD |
| 349 | Male | ≥65 | 95 | No | IV | Low | Mixed | SD/PD |
| 350 | Male | ≥65 | 97 | Yes | IV | Low | Mixed | SD/PD |
| 351 | Female | <65 | 98 | No | IV | Low | Mixed | SD/PD |
| 352 | Male | <65 | 100 | No | IV | Low | Intestinal | SD/PD |
| 353 | Male | ≥65 | 102 | No | IV | Moderate | Intestinal | SD/PD |
| 354 | Male | ≥65 | 103 | No | IV | Low | Mixed | SD/PD |
| 355 | Female | <65 | 103 | No | NA | Low | Diffuse | SD/PD |
| 356 | Female | <65 | 103 | Yes | IV | Low | Diffuse | SD/PD |
| 357 | Female | ≥65 | 104 | No | IV | Low | Intestinal | SD/PD |
| 358 | Male | ≥65 | 104 | No | IV | Low | Intestinal | SD/PD |
| 359 | Female | <65 | 104 | Yes | IV | Low | Diffuse | SD/PD |
| 360 | Male | <65 | 105 | No | IV | High | Intestinal | SD/PD |
| 361 | Female | <65 | 105 | No | III | Moderate | Intestinal | SD/PD |
| 362 | Male | <65 | 106 | Yes | IV | Low | Mixed | SD/PD |
| 363 | Male | ≥65 | 106 | No | IV | Low | Diffuse | SD/PD |
| 364 | Female | ≥65 | 109 | No | IV | Moderate | Intestinal | SD/PD |
| 365 | Male | <65 | 111 | No | IV | Low | Intestinal | SD/PD |
| 366 | Female | <65 | 112 | Yes | IV | Low | Mixed | SD/PD |
| 367 | Male | <65 | 112 | No | IV | Low | Diffuse | SD/PD |
| 368 | Male | ≥65 | 115 | No | IV | Low | Intestinal | SD/PD |
| 369 | Male | ≥65 | 115 | No | IV | Moderate | Intestinal | SD/PD |
| 370 | Female | <65 | 115 | Yes | IV | Low | Mixed | SD/PD |
| 371 | Male | ≥65 | 118 | Yes | NA | Low | Intestinal | SD/PD |
| 372 | Male | ≥65 | 120 | Yes | IV | Moderate | Intestinal | SD/PD |
| 373 | Male | <65 | 120 | No | IV | Low | Intestinal | SD/PD |
| 374 | Female | ≥65 | 120 | Yes | IV | Low | Intestinal | SD/PD |
| 375 | Male | ≥65 | 120 | Yes | IV | High | NA | SD/PD |
| 376 | Male | ≥65 | 121 | No | IV | Low | Intestinal | SD/PD |
| 377 | Male | <65 | 121 | No | IV | Low | Intestinal | SD/PD |
| 378 | Female | <65 | 121 | Yes | IV | Low | Diffuse | SD/PD |
| 379 | Male | <65 | 122 | No | IV | Moderate | Intestinal | SD/PD |
| 380 | Male | <65 | 124 | No | III | Low | Diffuse | SD/PD |
| 381 | Male | <65 | 125 | No | IV | Moderate | Intestinal | SD/PD |
| 382 | Male | <65 | 126 | No | III | Low | Mixed | SD/PD |
| 383 | Male | ≥65 | 127 | Yes | IV | Low | Mixed | SD/PD |
| 384 | Male | ≥65 | 127 | No | IV | Low | Intestinal | SD/PD |
| 385 | Male | ≥65 | 127 | No | IV | Moderate | Intestinal | SD/PD |
| 386 | Female | <65 | 127 | No | IV | Low | Mixed | SD/PD |
| 387 | Male | ≥65 | 127 | No | IV | Low | Mixed | SD/PD |
| 388 | Female | ≥65 | 127 | No | IV | Low | Intestinal | SD/PD |
| 389 | Male | ≥65 | 129 | No | IV | NA | Diffuse | SD/PD |
| 390 | Male | <65 | 129 | No | IV | Low | Diffuse | SD/PD |
| 391 | Male | <65 | 132 | No | IV | Moderate | Intestinal | SD/PD |
| 392 | Male | ≥65 | 132 | No | IV | Low | Intestinal | SD/PD |
| 393 | Male | ≥65 | 132 | No | IV | Moderate | Intestinal | SD/PD |
| 394 | Female | <65 | 133 | No | IV | Low | Diffuse | SD/PD |
| 395 | Male | <65 | 133 | No | III | Moderate | Intestinal | SD/PD |
| 396 | Male | ≥65 | 134 | No | IV | Moderate | Intestinal | SD/PD |
| 397 | Male | ≥65 | 134 | No | IV | Low | Intestinal | SD/PD |
| 398 | Female | ≥65 | 135 | No | III | Moderate | Intestinal | SD/PD |
| 399 | Male | ≥65 | 135 | Yes | IV | Low | Mixed | SD/PD |
| 400 | Male | ≥65 | 135 | Yes | IV | Low | Diffuse | SD/PD |
| 401 | Female | ≥65 | 137 | Yes | IV | NA | Diffuse | SD/PD |
| 402 | Female | <65 | 140 | No | IV | Moderate | Intestinal | SD/PD |
| 403 | Male | ≥65 | 140 | No | IV | NA | Intestinal | SD/PD |
| 404 | Male | <65 | 140 | No | IV | Moderate | Intestinal | SD/PD |
| 405 | Male | <65 | 140 | No | IV | Low | Mixed | SD/PD |
| 406 | Male | ≥65 | 141 | Yes | IV | Low | Diffuse | SD/PD |
| 407 | Female | <65 | 143 | Yes | IV | Low | Diffuse | SD/PD |
| 408 | Male | ≥65 | 144 | No | IV | NA | Diffuse | SD/PD |
| 409 | Female | <65 | 146 | Yes | IV | Low | Diffuse | SD/PD |
| 410 | Male | <65 | 147 | No | IV | Low | Intestinal | SD/PD |
| 411 | Male | <65 | 147 | Yes | IV | Low | Intestinal | SD/PD |
| 412 | Male | ≥65 | 148 | No | IV | Moderate | Intestinal | SD/PD |
| 413 | Female | ≥65 | 149 | No | IV | Low | Intestinal | SD/PD |
| 414 | Male | <65 | 150 | No | IV | Low | Intestinal | SD/PD |
| 415 | Female | ≥65 | 151 | No | III | NA | NA | SD/PD |
| 416 | Male | <65 | 151 | Yes | IV | Low | Mixed | SD/PD |
| 417 | Female | <65 | 151 | Yes | IV | Low | Diffuse | SD/PD |
| 418 | Female | <65 | 151 | Yes | IV | NA | Diffuse | SD/PD |
| 419 | Male | ≥65 | 152 | Yes | IV | Low | Diffuse | SD/PD |
| 420 | Male | ≥65 | 152 | Yes | IV | Low | Diffuse | SD/PD |
| 421 | Female | ≥65 | 154 | No | NA | Low | Diffuse | SD/PD |
| 422 | Female | <65 | 156 | No | IV | Low | Diffuse | SD/PD |
| 423 | Male | ≥65 | 156 | Yes | IV | Moderate | Intestinal | SD/PD |
| 424 | Female | ≥65 | 158 | No | IV | Low | Diffuse | SD/PD |
| 425 | Female | ≥65 | 158 | Yes | IV | Low | Intestinal | SD/PD |
| 426 | Male | ≥65 | 159 | No | IV | Moderate | Intestinal | SD/PD |
| 427 | Male | <65 | 160 | No | III | Low | Intestinal | SD/PD |
| 428 | Male | <65 | 164 | Yes | IV | Low | Intestinal | SD/PD |
| 429 | Female | ≥65 | 165 | Yes | IV | Low | Mixed | SD/PD |
| 430 | Male | ≥65 | 166 | No | IV | Moderate | Intestinal | SD/PD |
| 431 | Female | <65 | 168 | Yes | IV | Low | Mixed | SD/PD |
| 432 | Female | <65 | 169 | Yes | IV | Low | Diffuse | SD/PD |
| 433 | Female | <65 | 170 | Yes | IV | NA | Diffuse | SD/PD |
| 434 | Female | <65 | 170 | Yes | IV | Low | Diffuse | SD/PD |
| 435 | Male | ≥65 | 171 | No | III | Low | Intestinal | SD/PD |
| 436 | Male | <65 | 171 | No | IV | Moderate | Intestinal | SD/PD |
| 437 | Male | <65 | 172 | No | IV | Low | Diffuse | SD/PD |
| 438 | Male | ≥65 | 173 | Yes | IV | Low | Diffuse | SD/PD |
| 439 | Male | ≥65 | 174 | No | IV | Low | Mixed | SD/PD |
| 440 | Female | <65 | 178 | No | III | Low | Diffuse | SD/PD |
| 441 | Male | <65 | 178 | Yes | IV | Low | Diffuse | SD/PD |
| 442 | Male | <65 | 180 | No | IV | Low | Diffuse | SD/PD |
| 443 | Male | <65 | 181 | No | IV | Low | Intestinal | SD/PD |
| 444 | Female | <65 | 181 | Yes | IV | Low | Diffuse | SD/PD |
| 445 | Male | ≥65 | 184 | Yes | IV | Moderate | Intestinal | SD/PD |
| 446 | Male | ≥65 | 188 | Yes | IV | NA | Diffuse | SD/PD |
| 447 | Male | <65 | 189 | No | IV | Low | Mixed | SD/PD |
| 448 | Female | <65 | 189 | Yes | IV | Low | Mixed | SD/PD |
| 449 | Female | <65 | 190 | No | IV | Low | Diffuse | SD/PD |
| 450 | Male | <65 | 190 | No | IV | Low | Diffuse | SD/PD |
| 451 | Male | ≥65 | 192 | No | IV | Low | Mixed | SD/PD |
| 452 | Male | ≥65 | 192 | Yes | IV | Moderate | Mixed | SD/PD |
| 453 | Female | <65 | 193 | Yes | III | Low | Diffuse | SD/PD |
| 454 | Male | ≥65 | 200 | No | IV | Moderate | Intestinal | SD/PD |
| 455 | Male | ≥65 | 206 | Yes | IV | Moderate | Intestinal | SD/PD |
| 456 | Male | ≥65 | 209 | No | IV | Low | Mixed | SD/PD |
| 457 | Female | <65 | 209 | Yes | IV | Low | Mixed | SD/PD |
| 458 | Male | <65 | 209 | No | IV | Low | Mixed | SD/PD |
| 459 | Male | ≥65 | 211 | No | IV | Moderate | Intestinal | SD/PD |
| 460 | Male | ≥65 | 212 | No | IV | Low | Mixed | SD/PD |
| 461 | Female | <65 | 215 | No | IV | Moderate | Intestinal | SD/PD |
| 462 | Male | <65 | 217 | No | IV | NA | Intestinal | SD/PD |
| 463 | Male | <65 | 221 | Yes | IV | Low | Diffuse | SD/PD |
| 464 | Female | <65 | 227 | Yes | IV | Low | NA | SD/PD |
| 465 | Male | <65 | 233 | No | IV | Low | Mixed | SD/PD |
| 466 | Male | <65 | 233 | No | IV | Low | Mixed | SD/PD |
| 467 | Male | <65 | 240 | No | IV | Low | Diffuse | SD/PD |
| 468 | Female | <65 | 246 | Yes | IV | Low | Diffuse | SD/PD |
| 469 | Male | <65 | 247 | No | IV | Low | Diffuse | SD/PD |
| 470 | Male | <65 | 248 | No | IV | Low | NA | SD/PD |
| 471 | Male | <65 | 251 | No | IV | Low | Mixed | SD/PD |
| 472 | Female | <65 | 255 | No | IV | Low | Diffuse | SD/PD |
| 473 | Male | <65 | 255 | Yes | IV | Low | Diffuse | SD/PD |
| 474 | Male | <65 | 261 | Yes | IV | Moderate | Intestinal | SD/PD |
| 475 | Female | ≥65 | 294 | Yes | IV | Low | Diffuse | SD/PD |
| 476 | Male | ≥65 | 303 | No | IV | Moderate | Intestinal | SD/PD |
| 477 | Male | ≥65 | 322 | No | III | Low | Diffuse | SD/PD |
| 478 | Female | <65 | 324 | No | IV | Low | Diffuse | SD/PD |
| 479 | Female | <65 | 347 | Yes | IV | Low | Diffuse | SD/PD |
| 480 | Male | ≥65 | 351 | No | IV | Moderate | Intestinal | SD/PD |
| 481 | Male | ≥65 | 358 | No | IV | NA | Diffuse | SD/PD |
| 482 | Female | ≥65 | 361 | Yes | IV | Moderate | Intestinal | SD/PD |
| 483 | Male | ≥65 | 385 | No | IV | NA | Diffuse | SD/PD |
| 484 | Male | ≥65 | 386 | No | IV | Low | Mixed | SD/PD |
| 485 | Male | ≥65 | 393 | Yes | IV | Moderate | Intestinal | SD/PD |
| 486 | Male | ≥65 | 399 | No | IV | Moderate | Intestinal | SD/PD |
| 487 | Male | ≥65 | 409 | Yes | IV | Low | Mixed | SD/PD |
| 488 | Male | ≥65 | 428 | No | IV | Low | Intestinal | SD/PD |
| 489 | Male | ≥65 | 466 | Yes | IV | Low | Diffuse | SD/PD |
| 490 | Female | <65 | 504 | No | III | Low | Diffuse | SD/PD |
| 491 | Male | ≥65 | 540 | Yes | III | Low | Mixed | SD/PD |
| 492 | Male | ≥65 | 543 | No | IV | Low | Intestinal | SD/PD |
| 493 | Male | ≥65 | 551 | No | IV | Low | Intestinal | SD/PD |
| 494 | Male | ≥65 | 571 | No | IV | Low | Mixed | SD/PD |
| 495 | Male | ≥65 | 572 | No | IV | Moderate | Intestinal | SD/PD |
| 496 | Male | ≥65 | 926 | No | IV | Low | Intestinal | SD/PD |
| 497 | Male | <65 | 993 | Yes | IV | Low | Diffuse | SD/PD |
| 498 | Male | ≥65 | 1036 | No | IV | Low | Intestinal | SD/PD |
| 499 | Male | <65 | 1828 | No | IV | NA | Diffuse | SD/PD |
