# Supplementary material for: Single‐cell characterization of differentiation trajectories and drug resistance features in gastric cancer with peritoneal metastasis
Source: Clin Transl Med. 2024 Oct 18;14(10):e70054. doi: 10.1002/ctm2.70054 (PMC11488346; doi:10.1002/ctm2.70054)
Supplement: Supplementary file 9 — Supporting Information [file CTM2-14-e70054-s001.docx]

**Supplementary table 9.** Single-cell RNA sequencing dataset used for mapping diverse cell types to the spatial transcriptomic slides.

| **Sample ID** | **HER2 status** | **MMR status** | **Age** | **Gender** | **cTNM stage** | **Lauren type** |
| --- | --- | --- | --- | --- | --- | --- |
| 1 | 3+ | pMMR | 66 | Male | cT4aN3M1 | Mixed |
| 2 | 2+ | pMMR | 78 | Male | cT4bN3M1 | Mixed |
| 3 | 0 | dMMR | 80 | Male | cT4bN2M0 | Intestinal |
| 4 | 0 | pMMR | 74 | Female | cT4N1M1 | Mixed |

MMR, mismatch repair; dMMR, mismatch repair deficiency; pMMR, mismatch repair proficient.
