# Supplementary material for: Single‐cell characterization of differentiation trajectories and drug resistance features in gastric cancer with peritoneal metastasis
Source: Clin Transl Med. 2024 Oct 18;14(10):e70054. doi: 10.1002/ctm2.70054 (PMC11488346; doi:10.1002/ctm2.70054)
Supplement: Supplementary file 10 — Supporting Information [file CTM2-14-e70054-s004.docx]

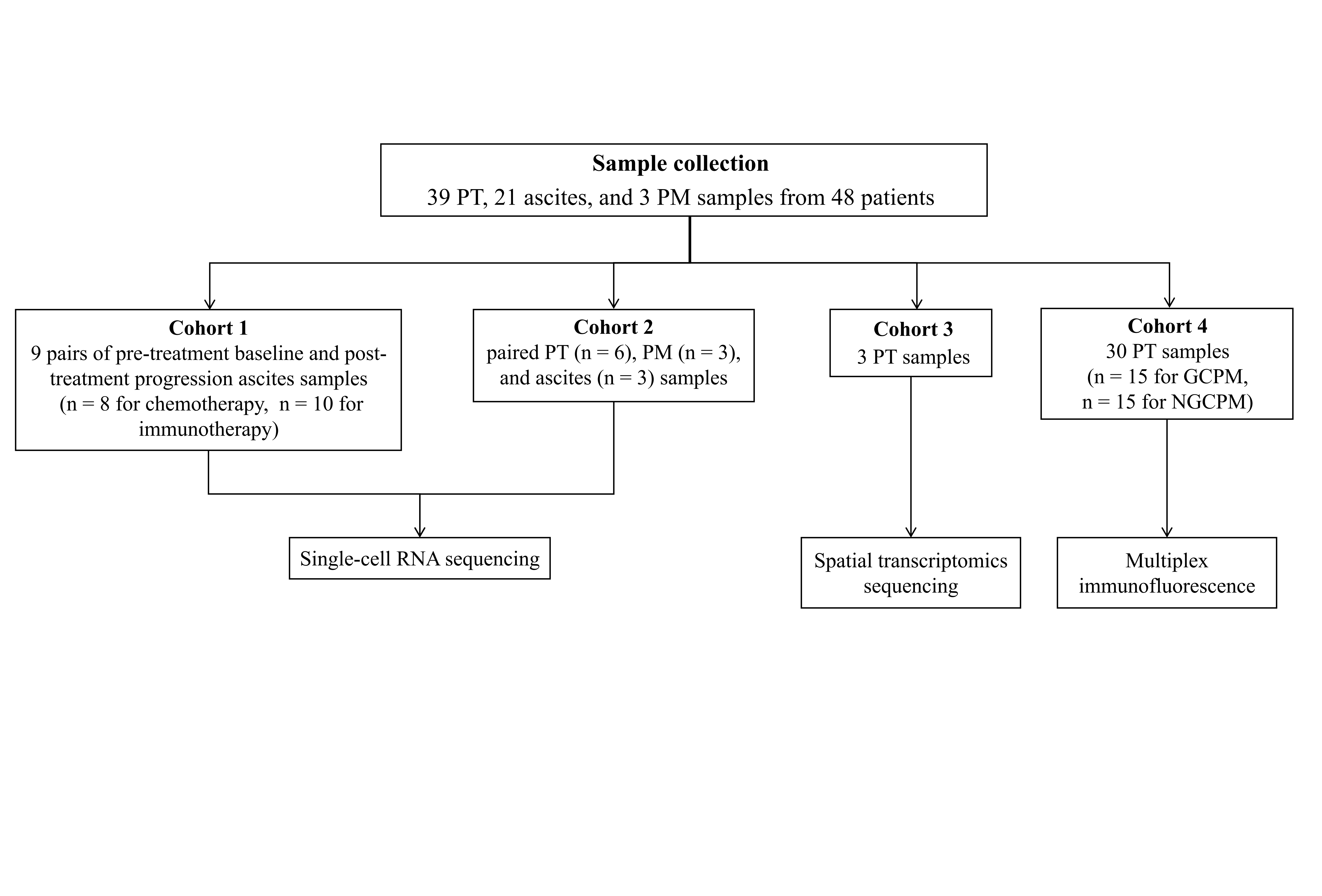


**Supplementary figure 1.** Schematic of the sample collection process of the three independent cohorts presented in this study. GCPM, gastric cancer peritoneal metastasis; PT, primary tumor; PM, peritoneal metastasis.


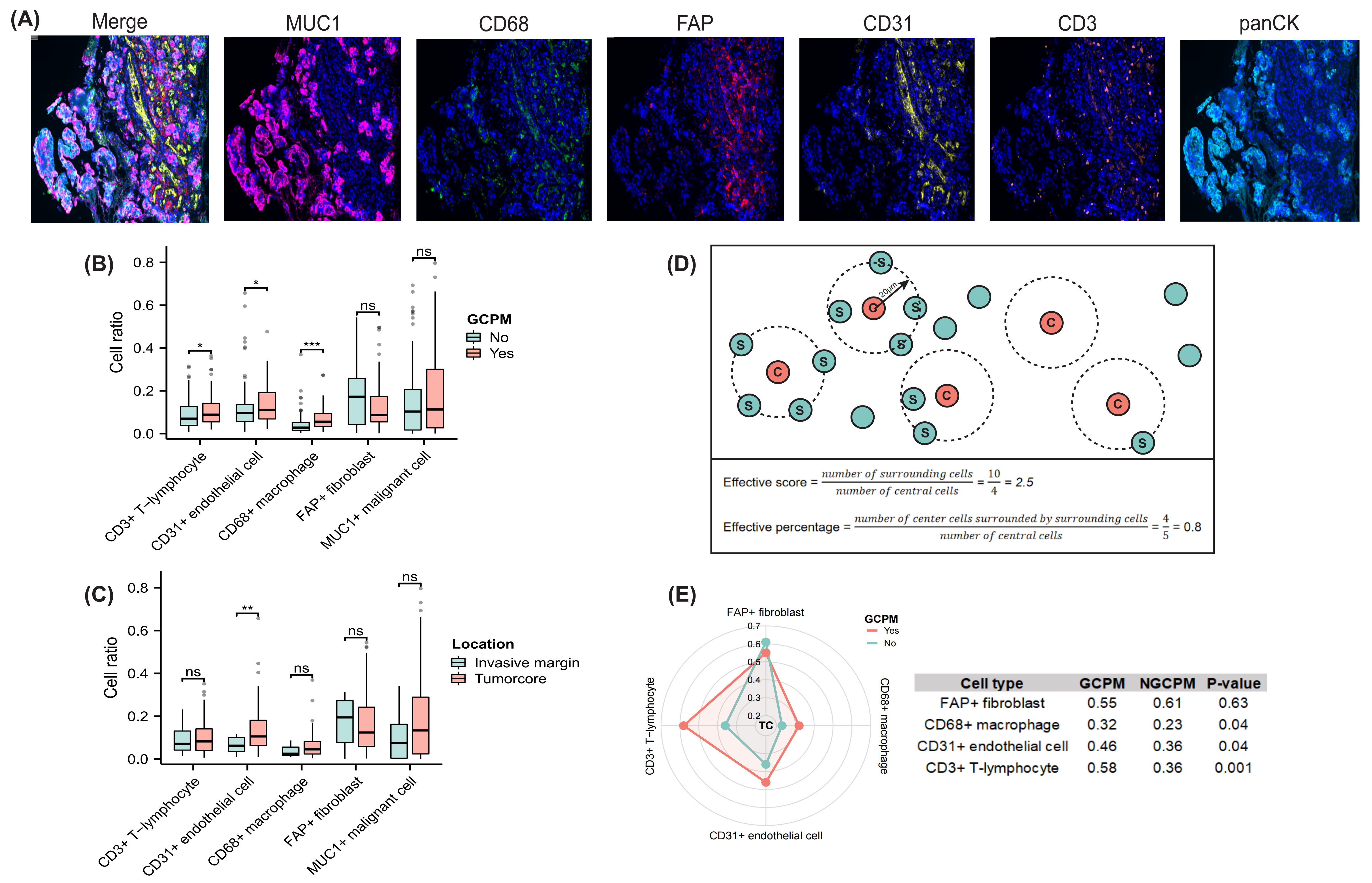


**Supplementary figure 2.** Characterization of the single-cell atlas of gastric cancer peritoneal metastasis. Dot plots showing expression levels of marker genes for specific cell clusters in cohort 1 (A) and cohort 2 (D), respectively. Dot size depicting the percentage of expressing cells, colored by standardized expression levels. UMAP plots and donut plots show the distinct cell composition of the included samples in cohort 1 (B, C) and cohort 2 (E, F), respectively.


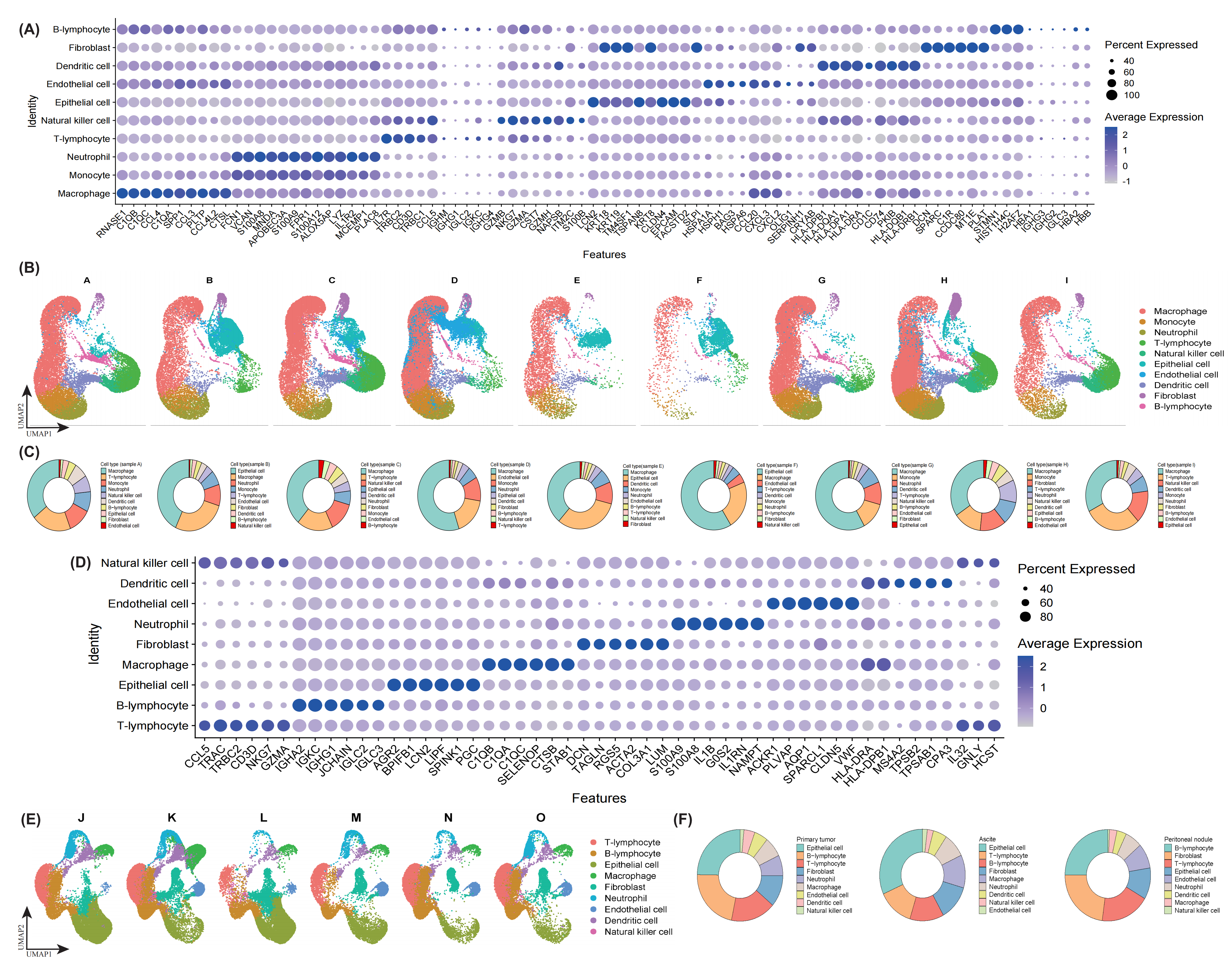


**Supplementary figure 3.** Multiplex immunofluorescence detection profiling the tumor microenvironment (TME) landscape of gastric cancer with peritoneal metastasis (GCPM). The expression levels and spatial distribution of six markers, including MUC1, CD68, FAP, CD31, CD3, and panCK in TME, were evaluated (A). Differences in infiltrating levels of various cell types between different GCPM status (B) and spatial location (C). Illustration of the spatial analysis calculating the effective score (ES) and effective percentage. Red dots represent the central cells, while blue dots represent the surrounding cells. The dashed circular outline represents the radius (20μm). Differences of spatial distances, as calculated by the ES indicator, between MUC1+ tumor cells and various cell types between cases with and without GCPM (E). *P < 0.05; ** P < 0.01; *** P < 0.001; ns, non-significant.


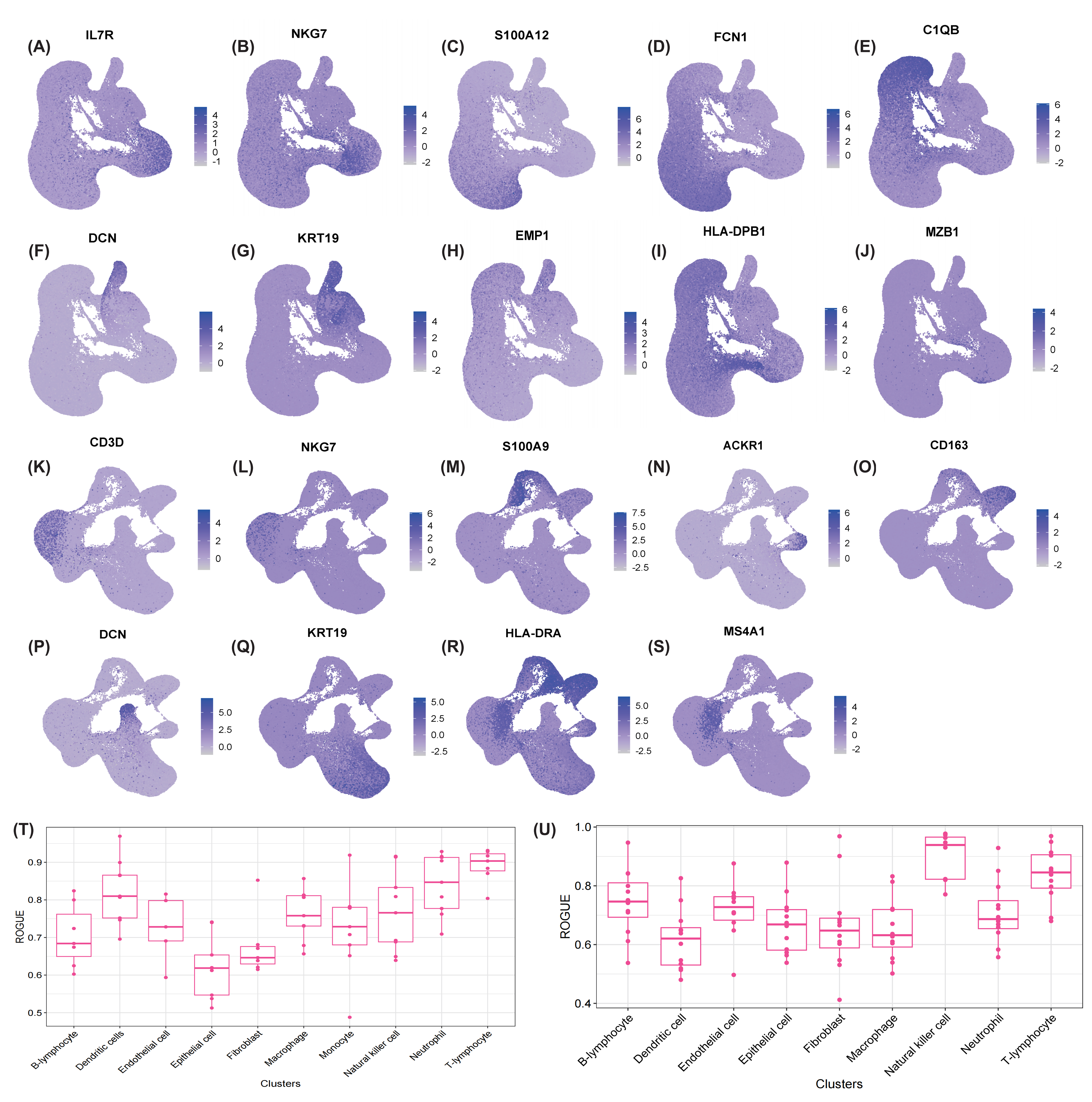


**Supplementary figure 4.** Canonical marker genes for identification and annotation of cell types in the single-cell RNA sequencing method. UMAP plots depicting the integrated cell atlas, colored according to the expression levels of canonical gene markers, including IL7R (A) and CCL5 (K) for T-lymphocytes, NKG7 (B) for natural killer cells, S100A12 (C) and S100A9 (L) for neutrophils, FCN1 (D) and TUBA1B (M) for monocytes, C1QB (E, N) for macrophage, DCN (F, O) for fibroblasts, KRT19 (G) and EPCAM (P) for epithelial cells, EMP1 (H) and MGP (Q) for endothelial cells, HLA-DPB1 (I) and HLA-DRA (R) for dendritic cells, and MZB1 (J, S) for B-lymphocytes. Evaluation of the purity of identified single-cell clusters by the ROGUE values in cohort 1 (T) and cohort 2 (U).


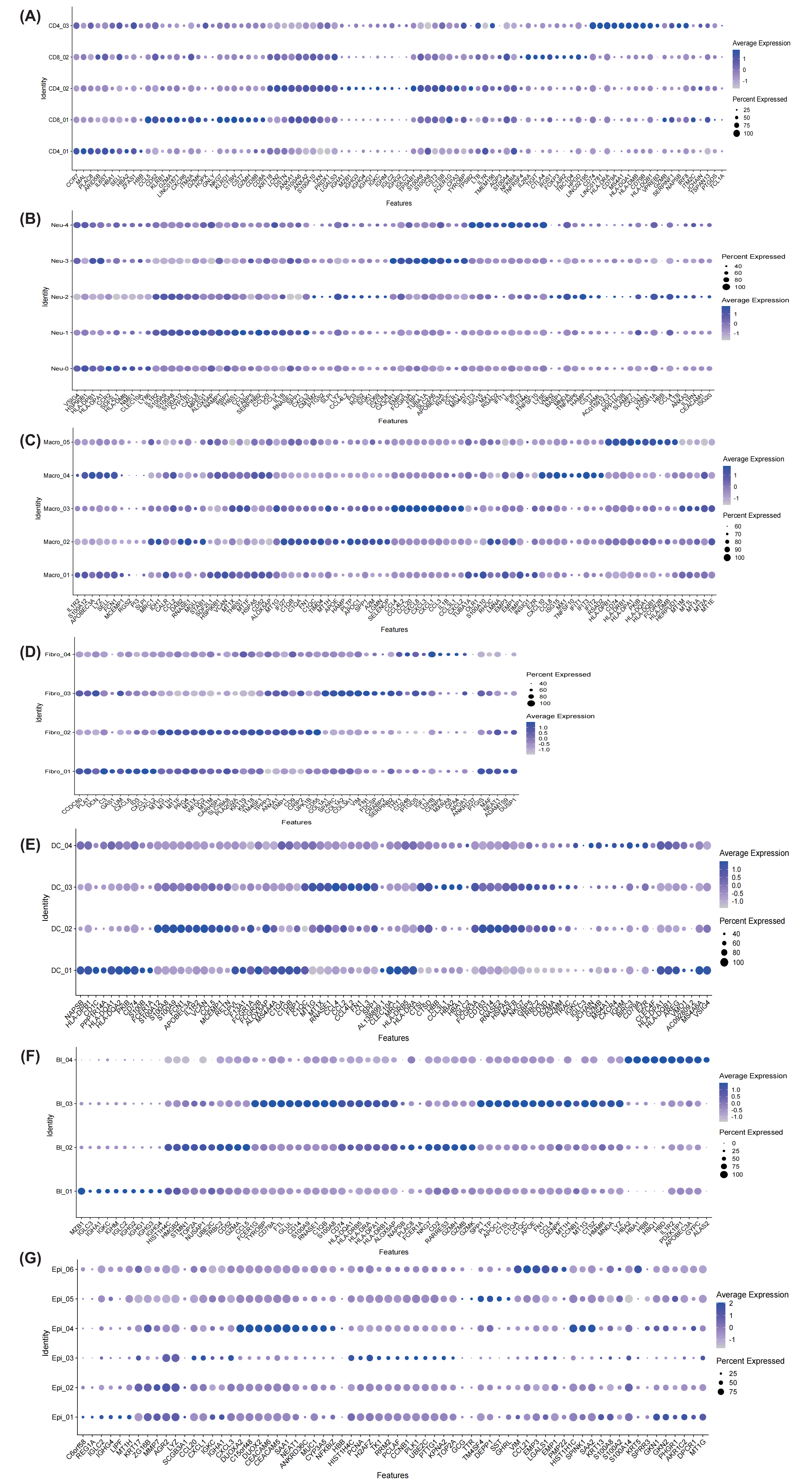


**Supplementary figure 5.** Characterization of major cell subclusters in the malignant ascites microenvironment by single-cell RNA sequencing. Dot plots showing expression levels of marker genes for cell subclusters of T-lymphocytes (A), neutrophils (B), macrophages (C), fibroblasts (D), dendritic cells (E), B-lymphocytes (F), and epithelial cells (G). Dot size depicting the percentage of expressing cells, colored by standardized expression levels.


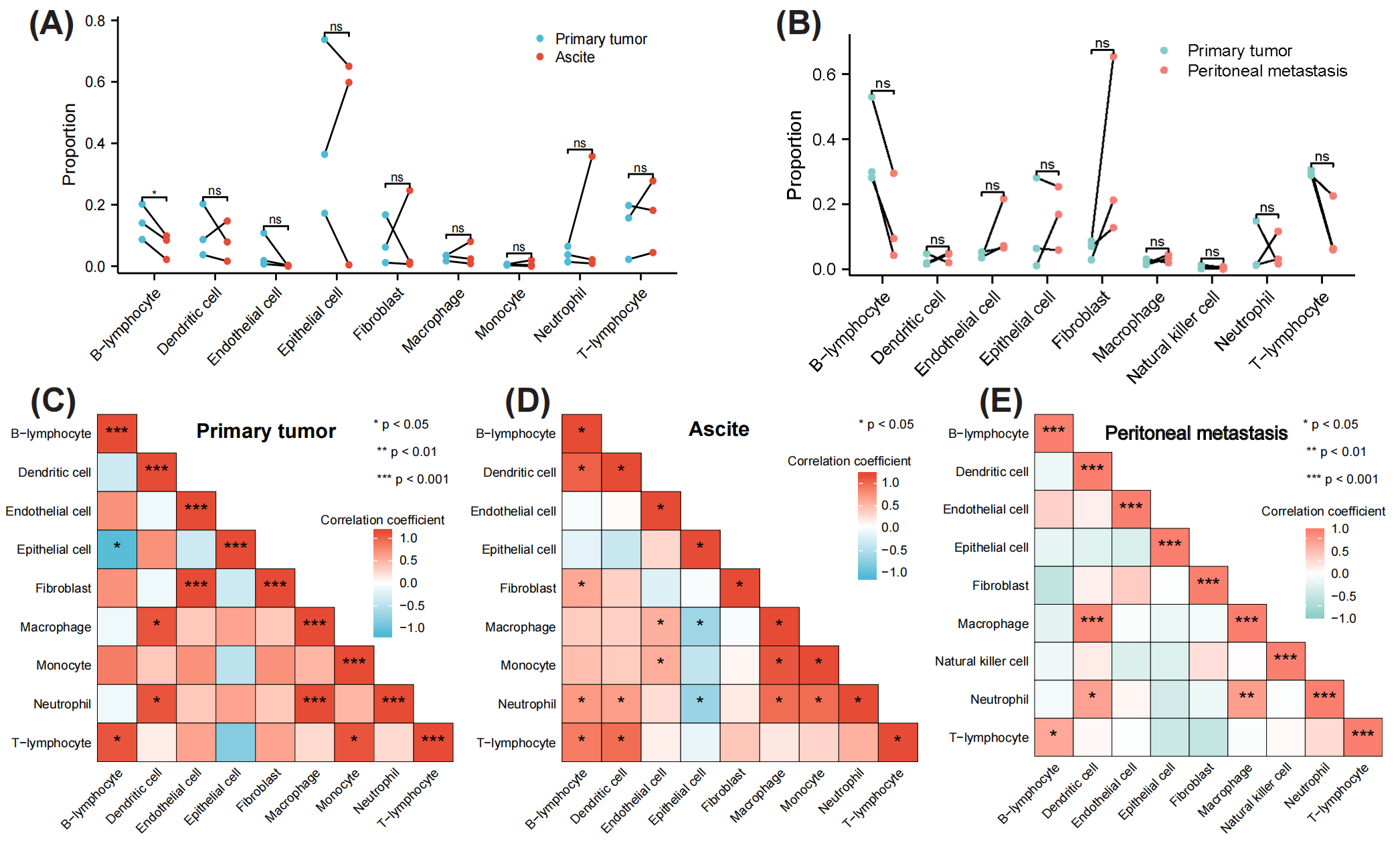


**Supplementary figure 6.** Differences in numerical associations between different cell types in the tumor microenvironment. Discrepancies in cellular composition between primary tumor and ascites microenvironment (A). Heatmaps display the differential correlations across single-cell clusters between the primary tumor microenvironment (B) and malignant ascites microenvironment (C), as evaluated by the Spearman rank correlation test. *P < 0.05; ** P < 0.01; *** P < 0.001.


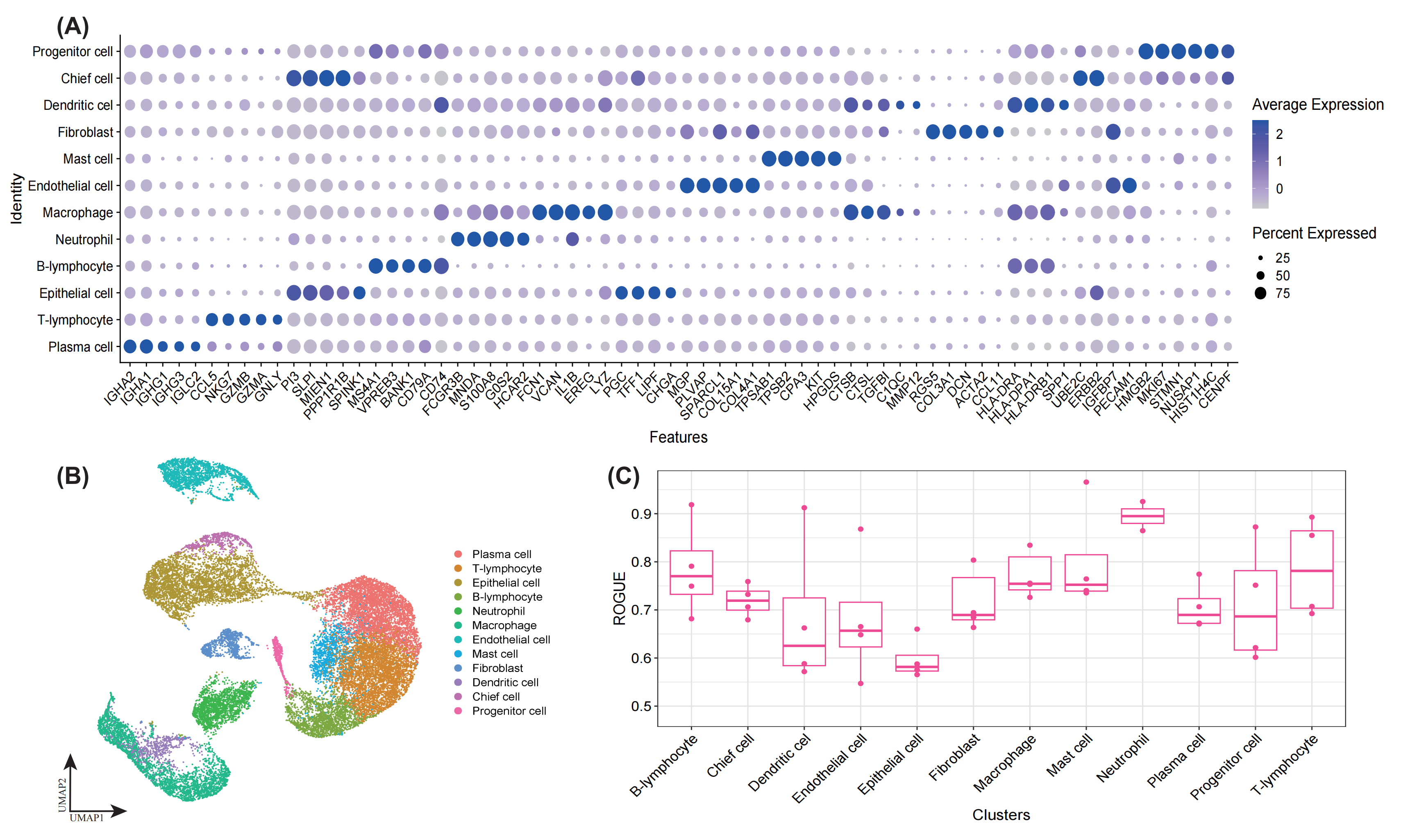


**Supplementary figure 7.** Single-cell RNA sequencing dataset used for mapping diverse cell types to the spatial transcriptomic slides. Dot plots showing expression levels of marker genes for each cell type (A). UMAP plots presenting the major cell populations (B). Evaluation of the purity of identified single-cell clusters by the ROGUE values (C).


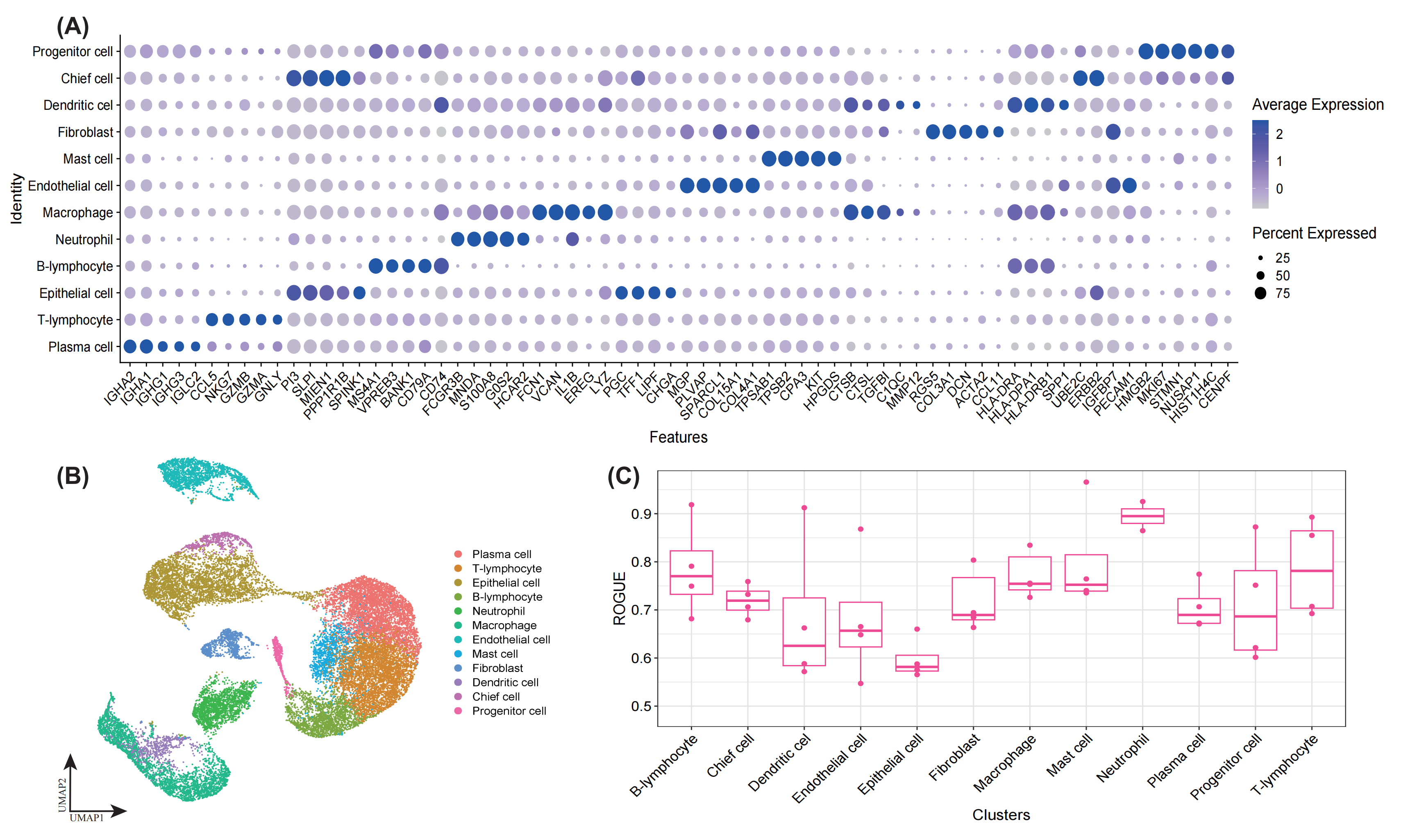


**Supplementary figure 8.** Spatial transcriptomic (ST) analysis on primary tumor samples from three patients with gastric cancer peritoneal metastasis. Dimensionality reduction and clustering of ST data of patient 1 (A), patient 2 (B), and patient 3 (C), respectively. Macrophages and T-lymphocytes tend to colocalize in the area of TC (D, I), and the potential ligand-receptor pair (E, J) and corresponding signaling pathway (F, K) that mediate their communication. Differentially expressed genes (G, L) and relevant enriched biological pathways (H, M) in the TC than the lymphoid follicle regions.


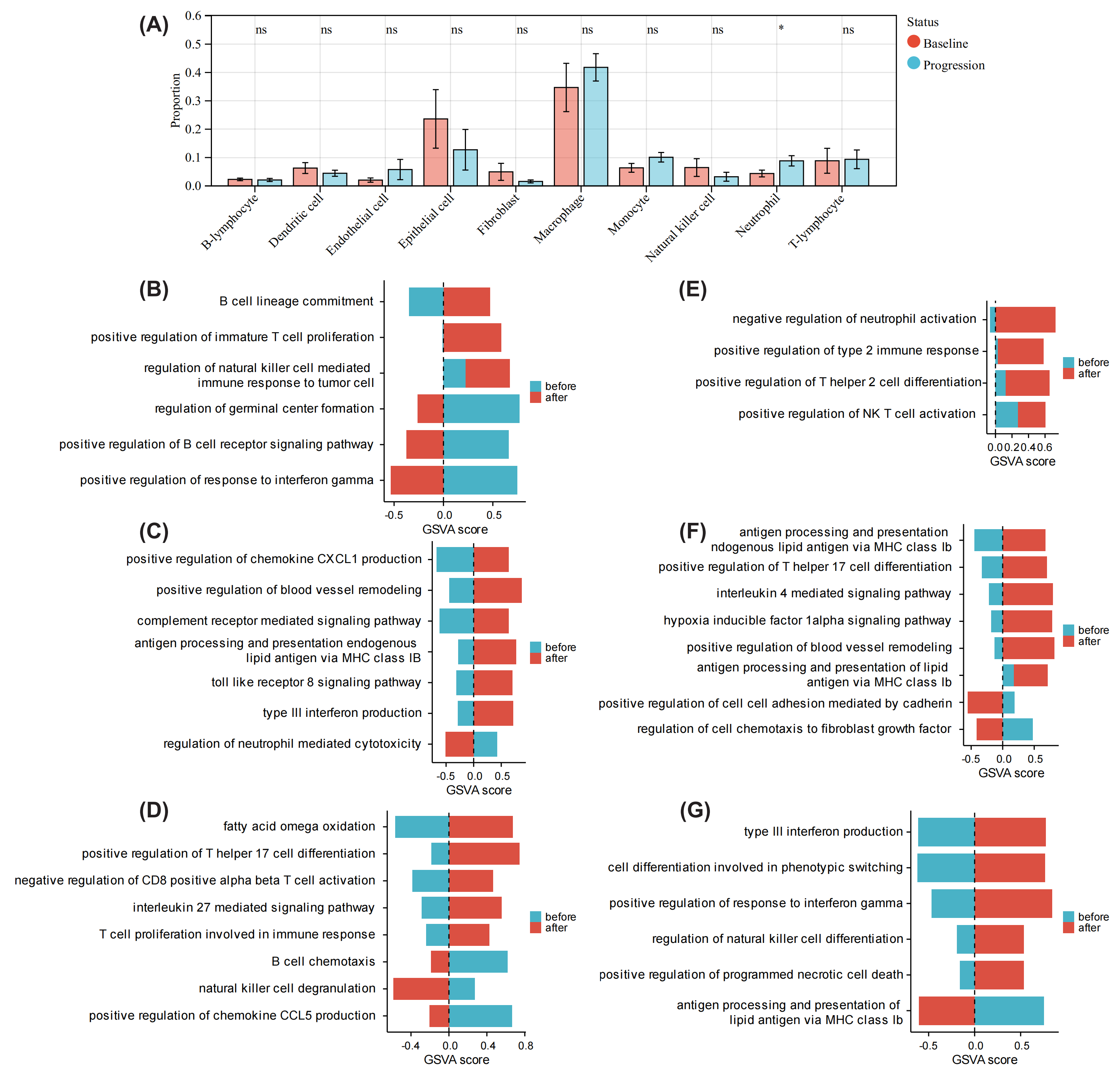


**Supplementary figure 9.** Therapy-induced functional reprogramming of major immune cell types in the malignant ascites microenvironment. Cellular composition changes between pre-treatment baseline and post-treatment progression samples (A). Chemotherapy and immunotherapy-induced cellular functional reprogramming of B-lymphocytes (B, E), macrophages (C, F), and T-lymphocytes (D, G), respectively. GSVA, gene set variation analysis.


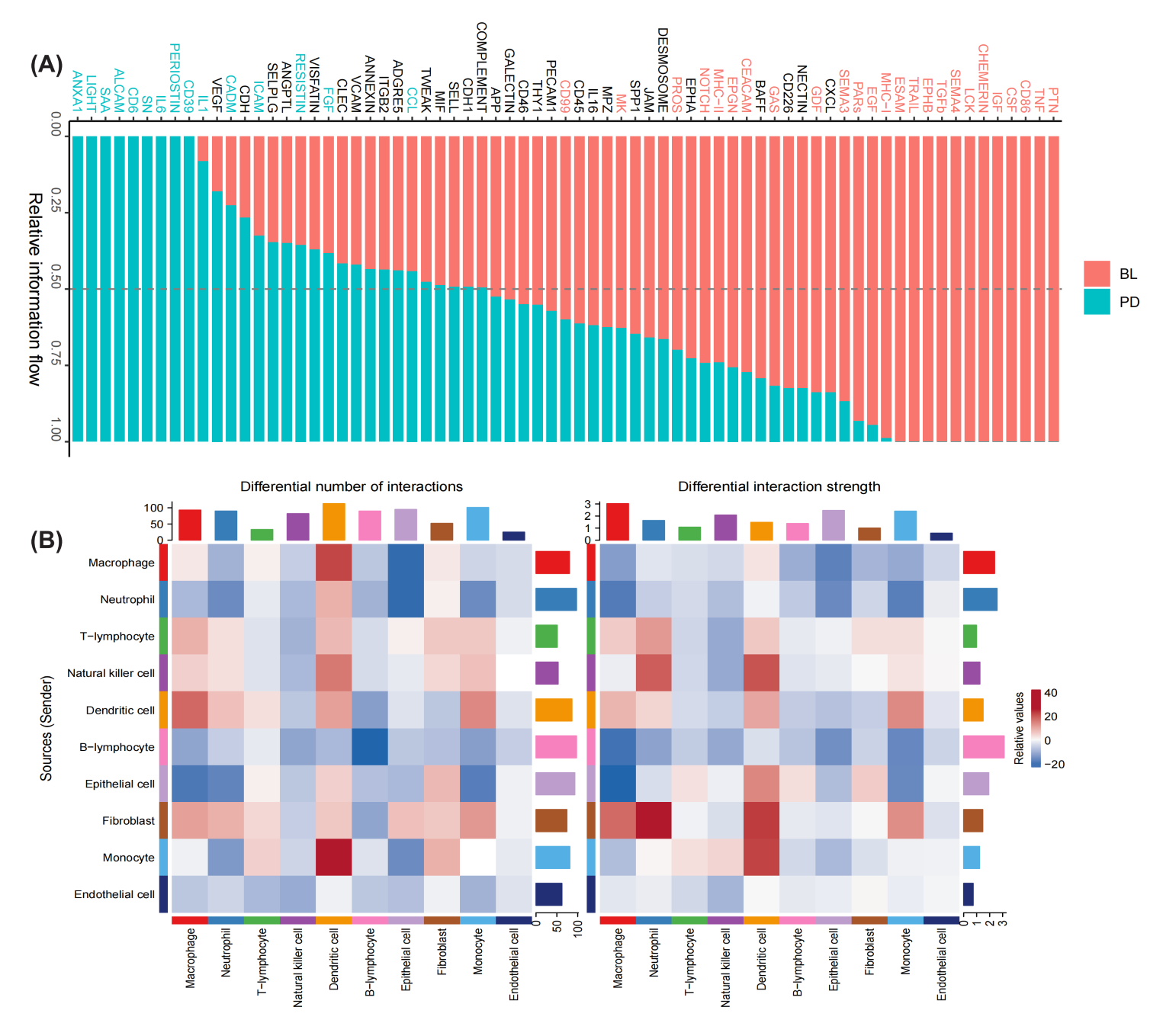


**Supplementary figure 10.** Discrepancies of intercellular interaction networks in malignant ascites microenvironment between progression and baseline samples. Comparison of the overall information flow of each signaling pathway (A) and differential interaction levels among different cell types (B) between progression and baseline samples.


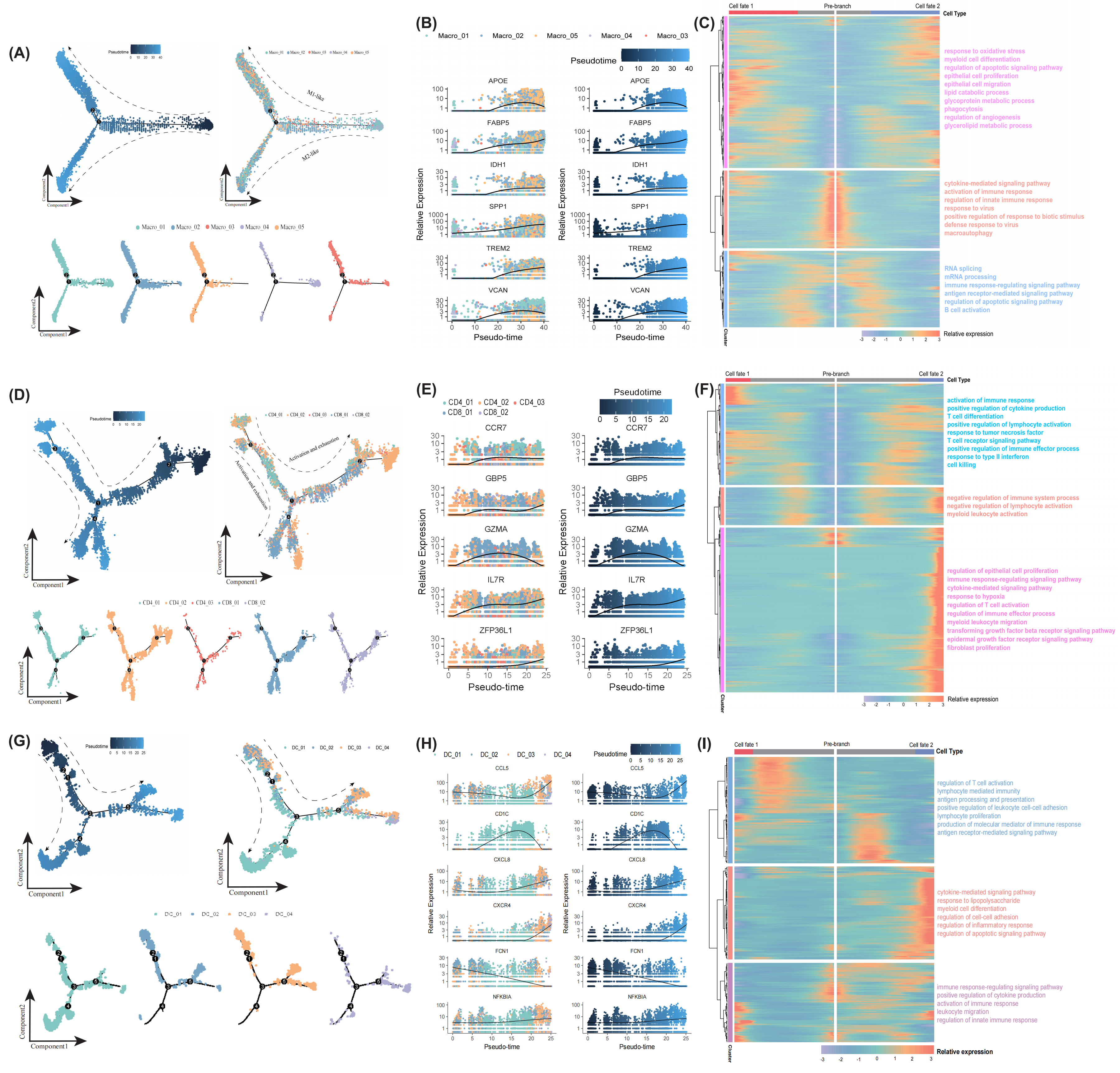


**Supplementary figure 11.** Differentiation trajectories of major immune cell types within peritoneal microenvironment. Lineage progression of macrophages (A), T-lymphocytes (D), and dendritic cells (G) along the pseudotime track, with each point representing a single cell. Dynamic changes in function-related genes during the transitional trajectory and relevant enriched pathways of macrophages (B, C), T-lymphocytes (E, F), and dendritic cells (H, I), respectively.


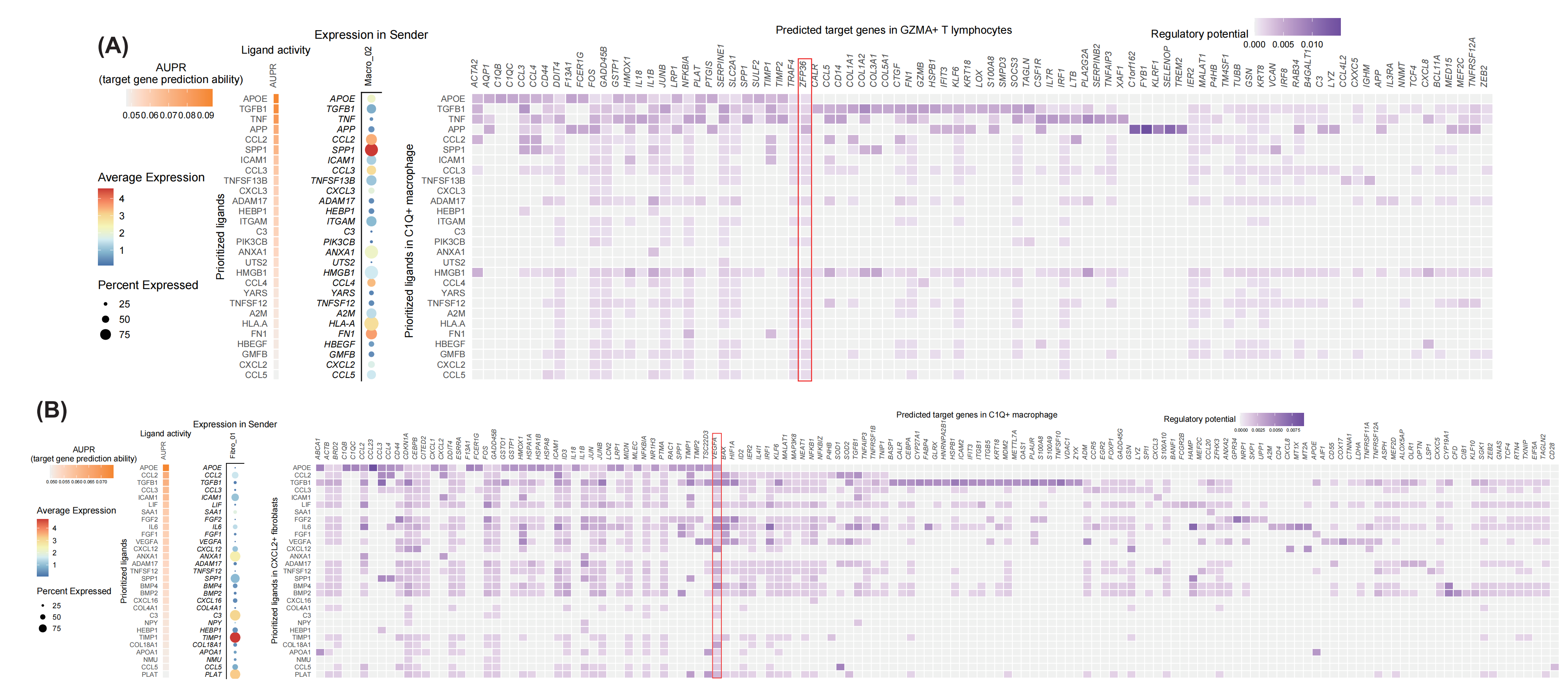


**Supplementary figure 12.** NicheNet algorithm profiles the intracellular gene regulation effects and signal transductions in treatment failure cases. The ligand activity, expression spectrums, and regulatory potential of the prioritized ligands in C1Q+ macrophages that drive GZMA+ T lymphocyte dysfunction in immunoresistant cases (A). The ligand activity, expression profiles, and regulatory potential of the prioritized ligands in CXCL2+ fibroblasts that drive the pro-angiogenesis function of C1Q+ macrophages in chemoresistant cases (B).


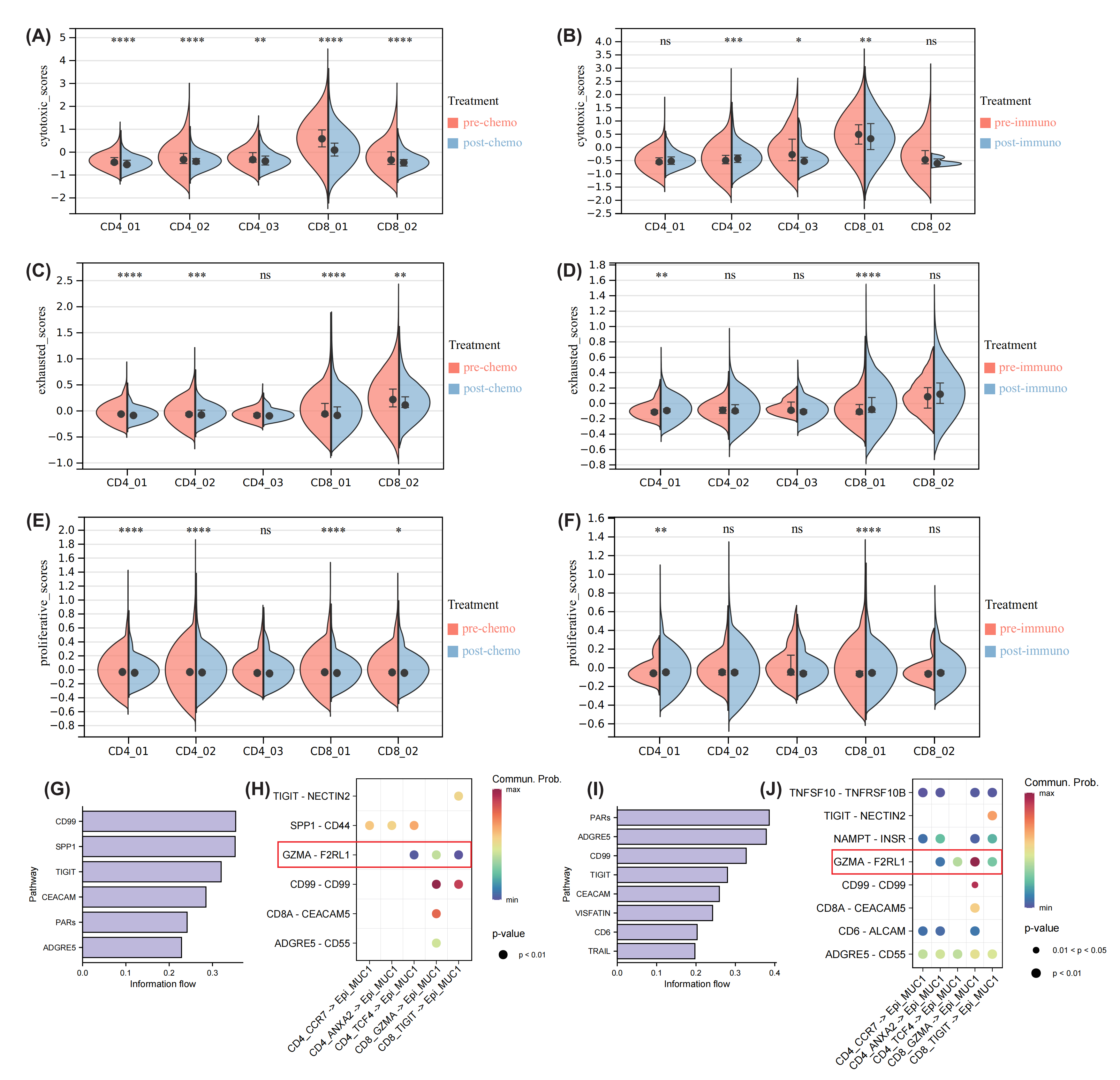


**Supplementary figure 13.** Characterization of therapy-induced functional remodeling of T-lymphocytes in the malignant ascites microenvironment. Single-sample Gene Set Enrichment Analysis evaluates the alterations of cytotoxic (A, B), exhausted (C, D), and proliferative (E, F) scores of different T-lymphocyte subsets before and after chemotherapy/immunotherapy. Differences in the overall signaling pathway with MUC1+ malignant cells as signal receivers and their interaction probabilities with T-lymphocytes between chemotherapy (G, H) and immunotherapy (I, J) cohorts.


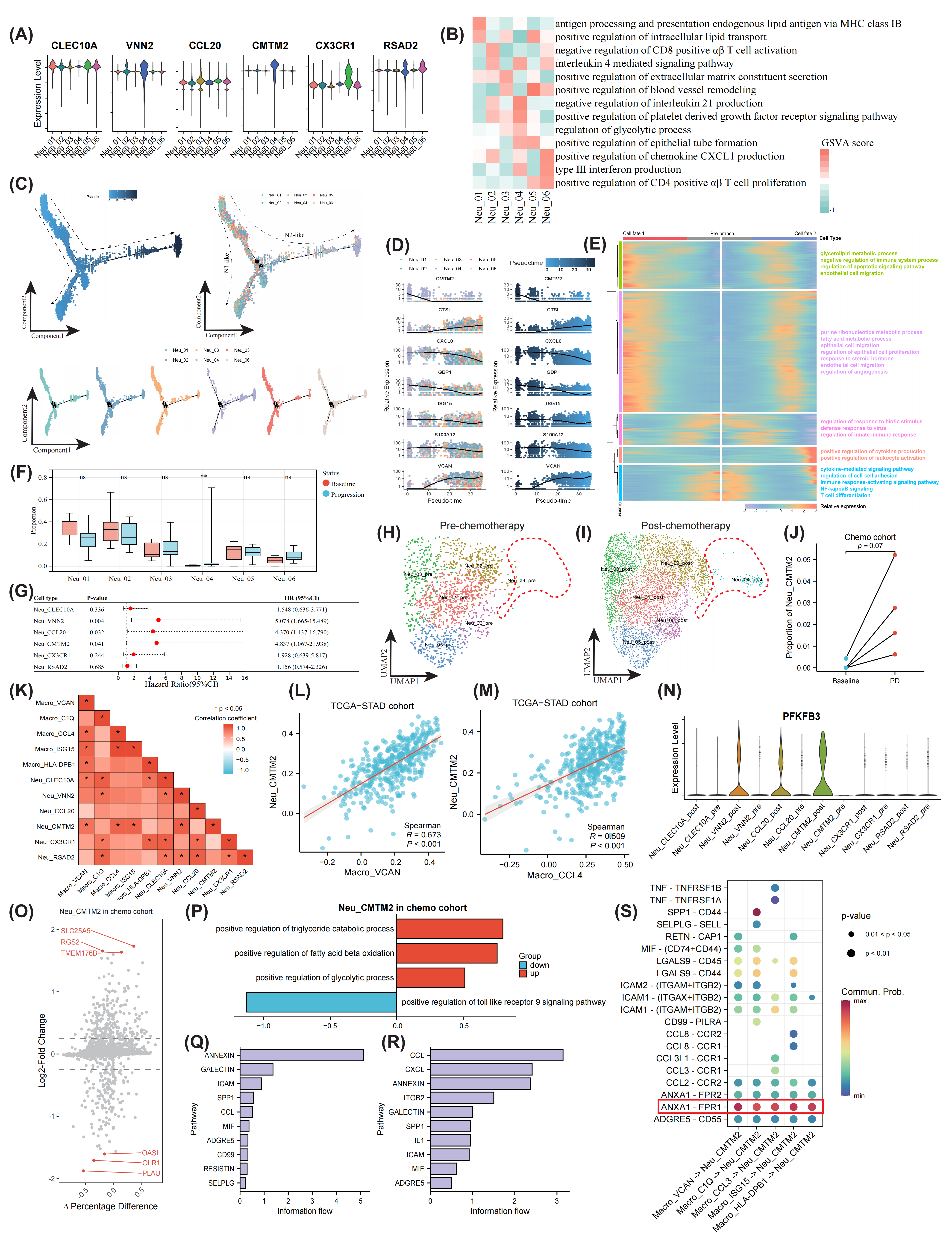


**Supplementary figure 14.** Single-cell level characterization of the neutrophils within ascites microenvironment. Expression profiles of selected marker genes (A) and functional disparities (B) among different neutrophil subsets. The inferred development dynamics of neutrophil subtypes (C). Dynamical expression levels of function-related genes (D), encompassing CMTM2, CTSL, CXCL8, GBP1, ISG15, S100A12, and VCAN, and relevant hierarchically clustered groups with distinct pathway activities (E) along the neutrophil trajectory. Cellular proportion changes of neutrophils in progression than baseline samples (F). Forest plot depicting the prognostic values of each neutrophil subcluster in the TCGA-STAD cohort, as evaluated by the univariate Cox regression analysis (G). UMAP plots indicate the distinct cellular composition of neutrophil subpopulations in post- (I) versus pre- (H) samples. The proportion of CMTM2+ neutrophils significantly increased in chemotherapy-resistant cases (J). Heatmap reveals strong correlations between different neutrophil and macrophage subtypes in the chemotherapy cohort (K), which are further validated in the TCGA-STAD dataset (L, M). The expression profiles of PFKFB3 across different neutrophil subtypes (N). Volcano plot displays the differentially expressed genes between the progression and baseline samples undergoing chemotherapy (O). Gene set variation analysis underscores the prominent metabolic reprogramming of CMTM2+ neutrophils in chemoresistant cases (P). Differences in the overall information flow of each signal pathway with CMTM2+ neutrophils as signal receivers and their interaction probabilities with macrophages mediated by ligand-receptor pairs between chemotherapy (Q, S) and immunotherapy cohorts (R). *P < 0.05; ** P < 0.01; ns, non-significant.


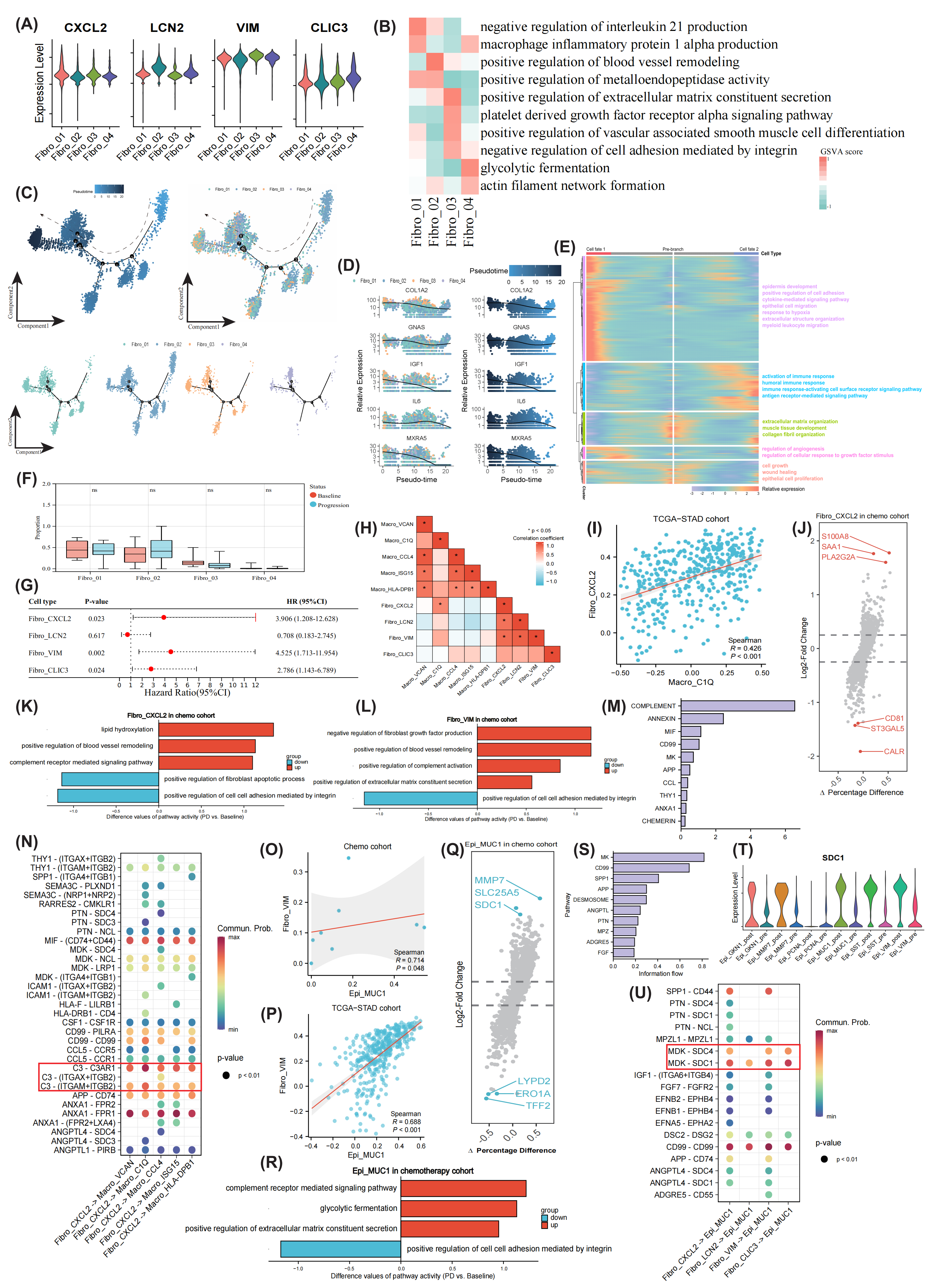


**Supplementary figure 15.** Characterization of the fibroblasts in ascites microenvironment at single-cell level. Violin plot showing the highly expressed marker genes of each fibroblast subset (A). Heatmap indicating functional differences among different fibroblast subtypes, with intensity of color showing gene set variation analysis scores (B). Monocle analysis for trajectory inference of the fibroblast subclusters (C). Expression alterations of representative genes along the pseudotime trajectory, including COL1A3, GNAS, IGF1, IL6, and MXRA5 (D). Pseudotime-related differentially expressed genes (DEGs) and corresponding enriched pathways (E). Box plot showing the proportion of changes in different fibroblast subtypes in the progression than the baseline samples (F). Forest plot demonstrating the prognostic significance of different fibroblast gene signatures in the TCGA-STAD dataset (G). Heatmap underscored the positive association between CXCL2+ fibroblast and C1Q+ macrophage infiltrates in the immunotherapy cohort (H), validated in the TCGA-STAD cohort (I). Volcano plot indicates the DEGs in CXCL2+ fibroblasts between pre- and post-chemotherapy (J). Functional changes of CXCL2+ fibroblasts (K) and VIM+ fibroblasts (L) in chemoresistant cases. Summary of putative communications from CXCL2+ fibroblasts to different macrophage subsets by the overall information flow (M) and the ligand-receptor pairs (N) in the chemotherapy cohort. Scatter plots indicating positive associations between MUC1+ malignant cells and VIM+ fibroblasts infiltrate in both the immunotherapy (O) and TCGA-STAD (P) cohorts. Volcano plot highlights the DEGs in MUC1+ malignant cells between pre- and post-chemotherapy (Q), with relevant functional changes (R). Summary of putative communications from different fibroblast subsets to MUC1+ malignant cells by the overall information flow (S) and the ligand-receptor pairs (U) in the chemotherapy cohort. The expression spectrums of SDC1 among different epithelial subtypes. *P < 0.05; ns, non-significant.


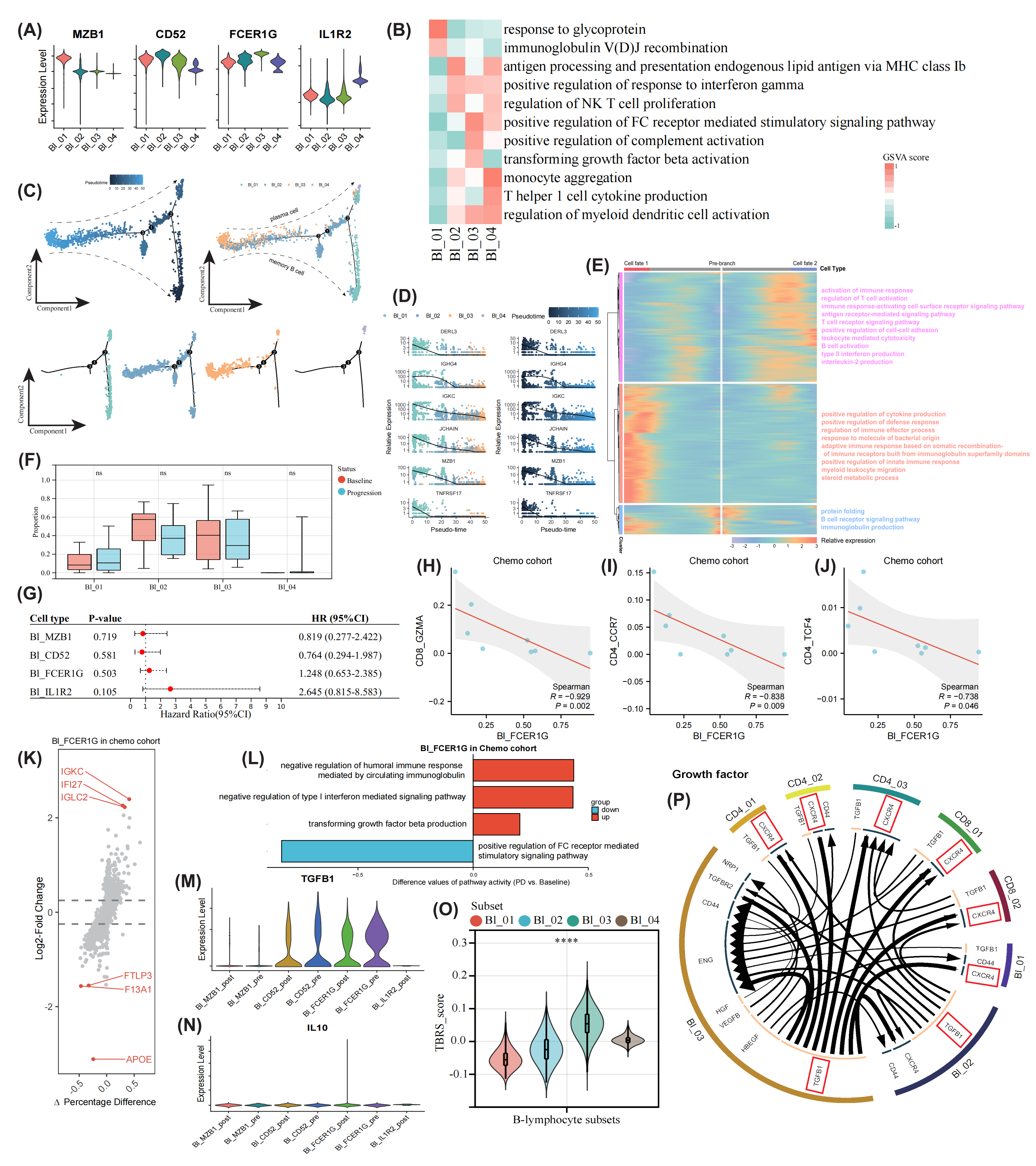


**Supplementary figure 16.** Delineation of the single-cell transcriptomic landscape of B-lymphocytes in ascites microenvironment. Violin plot depicts the highly expressed marker genes of each B-lymphocyte subset (A). Heatmap indicates functional discrepancies among different subtypes, with the intensity of color showing gene set variation analysis scores (B). Monocle analysis for trajectory inference of the four subclusters (C). Expression changes of representative genes along the pseudotime trajectory, including DERL3, IGHG4, IGKC, JCHAIN, MZB1, and TNFTSF17 (D). Pseudotime-related differentially expressed genes (DEGs) and corresponding enriched pathways (E). Box plot shows the proportion changes of different subtypes in the progression than baseline samples (F). Forest plot demonstrating the prognostic significance of different B-lymphocyte gene signatures in the TCGA-STAD dataset (G). Robust and negative associations between FCER1G+ B-lymphocytes and GZMA+ (H), CCR7+ (I), and TCF4+ (J) T-lymphocytes. Volcano plot highlights the DEGs in FCER1G+ B-lymphocytes after chemotherapy (K). Functional changes of FCER1G+ B-lymphocytes in chemoresistant cases (L). The expression profiles of TGF-β (M) and IL10 (N) among distinct B-lymphocyte subsets. The TGF-β response signature scores of different subsets (O). iTALK algorithm inferred the cellular interaction networks between various T and B lymphocyte subtypes (P).


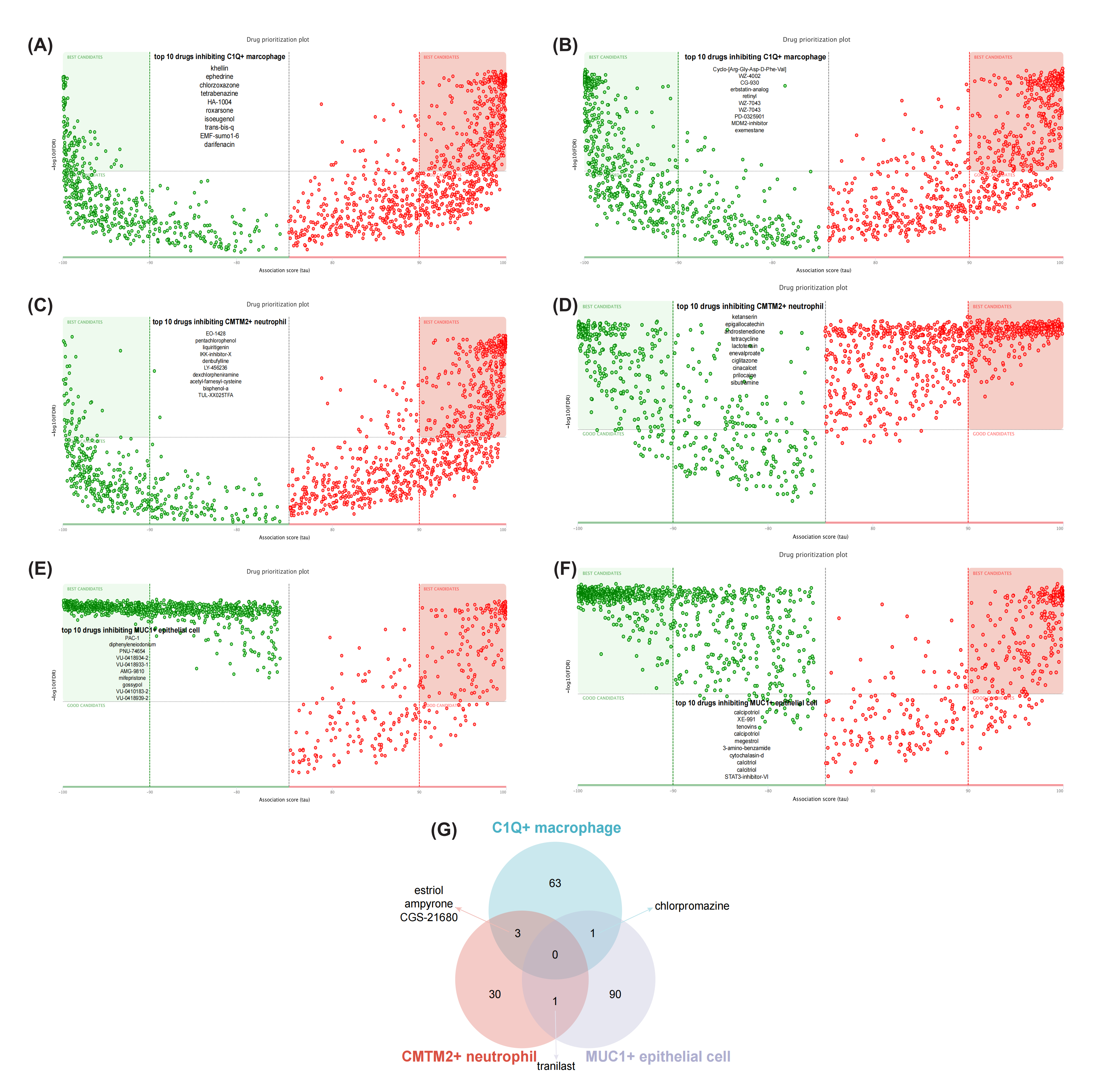


**Supplementary figure 17.** Potential immunomodulatory drugs targeting key cell subpopulations that drive chemotherapy and immunotherapy resistance. Drug prioritization analyses investigating potential immunomodulatory drugs targeting C1Q+ macrophages (A, B), CMTM2+ neutrophils (C, D), and MUC1+ epithelial cells (E, F) in chemotherapy and immunotherapy failure cases, respectively. The intersection of potential immunomodulatory drugs targeting key cell subpopulations that drive chemotherapy and immunotherapy resistance (G).
